# Sex-dimorphic genetic effects and novel loci for fasting glucose and insulin variability

**DOI:** 10.1038/s41467-020-19366-9

**Published:** 2021-01-05

**Authors:** Vasiliki Lagou, Reedik Mägi, Jouke- Jan Hottenga, Harald Grallert, John R. B. Perry, Nabila Bouatia-Naji, Letizia Marullo, Denis Rybin, Rick Jansen, Josine L. Min, Antigone S. Dimas, Anna Ulrich, Liudmila Zudina, Jesper R. Gådin, Longda Jiang, Alessia Faggian, Amélie Bonnefond, Joao Fadista, Maria G. Stathopoulou, Aaron Isaacs, Sara M. Willems, Pau Navarro, Toshiko Tanaka, Anne U. Jackson, May E. Montasser, Jeff R. O’Connell, Lawrence F. Bielak, Rebecca J. Webster, Richa Saxena, Jeanette M. Stafford, Beate St Pourcain, Nicholas J. Timpson, Perttu Salo, So-Youn Shin, Najaf Amin, Albert V. Smith, Guo Li, Niek Verweij, Anuj Goel, Ian Ford, Paul C. D. Johnson, Toby Johnson, Karen Kapur, Gudmar Thorleifsson, Rona J. Strawbridge, Laura J. Rasmussen-Torvik, Tõnu Esko, Evelin Mihailov, Tove Fall, Ross M. Fraser, Anubha Mahajan, Stavroula Kanoni, Vilmantas Giedraitis, Marcus E. Kleber, Günther Silbernagel, Julia Meyer, Martina Müller-Nurasyid, Andrea Ganna, Antti-Pekka Sarin, Loic Yengo, Dmitry Shungin, Jian’an Luan, Momoko Horikoshi, Ping An, Serena Sanna, Yvonne Boettcher, N. William Rayner, Ilja M. Nolte, Tatijana Zemunik, Erik van Iperen, Peter Kovacs, Nicholas D. Hastie, Sarah H. Wild, Stela McLachlan, Susan Campbell, Ozren Polasek, Olga Carlson, Josephine Egan, Wieland Kiess, Gonneke Willemsen, Johanna Kuusisto, Markku Laakso, Maria Dimitriou, Andrew A. Hicks, Rainer Rauramaa, Stefania Bandinelli, Barbara Thorand, Yongmei Liu, Iva Miljkovic, Lars Lind, Alex Doney, Markus Perola, Aroon Hingorani, Mika Kivimaki, Meena Kumari, Amanda J. Bennett, Christopher J. Groves, Christian Herder, Heikki A. Koistinen, Leena Kinnunen, Ulf de Faire, Stephan J. L. Bakker, Matti Uusitupa, Colin N. A. Palmer, J. Wouter Jukema, Naveed Sattar, Anneli Pouta, Harold Snieder, Eric Boerwinkle, James S. Pankow, Patrik K. Magnusson, Ulrika Krus, Chiara Scapoli, Eco J. C. N. de Geus, Matthias Blüher, Bruce H. R. Wolffenbuttel, Michael A. Province, Goncalo R. Abecasis, James B. Meigs, G. Kees Hovingh, Jaana Lindström, James F. Wilson, Alan F. Wright, George V. Dedoussis, Stefan R. Bornstein, Peter E. H. Schwarz, Anke Tönjes, Bernhard R. Winkelmann, Bernhard O. Boehm, Winfried März, Andres Metspalu, Jackie F. Price, Panos Deloukas, Antje Körner, Timo A. Lakka, Sirkka M. Keinanen-Kiukaanniemi, Timo E. Saaristo, Richard N. Bergman, Jaakko Tuomilehto, Nicholas J. Wareham, Claudia Langenberg, Satu Männistö, Paul W. Franks, Caroline Hayward, Veronique Vitart, Jaakko Kaprio, Sophie Visvikis-Siest, Beverley Balkau, David Altshuler, Igor Rudan, Michael Stumvoll, Harry Campbell, Cornelia M. van Duijn, Christian Gieger, Thomas Illig, Luigi Ferrucci, Nancy L. Pedersen, Peter P. Pramstaller, Michael Boehnke, Timothy M. Frayling, Alan R. Shuldiner, Patricia A. Peyser, Sharon L. R. Kardia, Lyle J. Palmer, Brenda W. Penninx, Pierre Meneton, Tamara B. Harris, Gerjan Navis, Pim van der Harst, George Davey Smith, Nita G. Forouhi, Ruth J. F. Loos, Veikko Salomaa, Nicole Soranzo, Dorret I. Boomsma, Leif Groop, Tiinamaija Tuomi, Albert Hofman, Patricia B. Munroe, Vilmundur Gudnason, David S. Siscovick, Hugh Watkins, Cecile Lecoeur, Peter Vollenweider, Anders Franco-Cereceda, Per Eriksson, Marjo-Riitta Jarvelin, Kari Stefansson, Anders Hamsten, George Nicholson, Fredrik Karpe, Emmanouil T. Dermitzakis, Cecilia M. Lindgren, Mark I. McCarthy, Philippe Froguel, Marika A. Kaakinen, Valeriya Lyssenko, Richard M. Watanabe, Erik Ingelsson, Jose C. Florez, Josée Dupuis, Inês Barroso, Andrew P. Morris, Inga Prokopenko, Meta-Analyses of Glucose and Insulin-related traits Consortium (MAGIC)

**Affiliations:** 1 Wellcome Centre for Human Genetics, University of Oxford, Oxford, United Kingdom; 2 Department of Microbiology and Immunology, Laboratory of Adaptive Immunity, KU Leuven Leuven, Belgium; 3 VIB-KU Leuven Center for Brain and Disease Research, Leuven, Belgium; 4 Estonian Genome Centre, Institute of Genomics, University of Tartu, Tartu, Estonia; 5 Department of Biological Psychology, Vrije Universiteit, Amsterdam, the Netherlands; 6 Amsterdam Public Health Research Institute, VU University medical center, Amsterdam, the Netherlands; 7 Research Unit of Molecular Epidemiology, Institute of Epidemiology, Helmholtz Zentrum München Research Center for Environmental Health, Neuherberg, Germany; 8 German Center for Diabetes Research (DZD, München-Neuherberg, Germany; 9 MRC Epidemiology Unit, University of Cambridge School of Clinical Medicine, Cambridge, United Kingdom; 10 University of Lille Nord de France, Lille, France; 11 CNRS UMR8199, Institut Pasteur de Lille, Lille, France; 12 INSERM U970, Paris Cardiovascular Research Center PARCC, 75006 Paris, France; 13 Department of Life Sciences and Biotechnology, University of Ferrara, Ferrara, Italy; 14 Boston University Data Coordinating Center, Boston, MA USA; 15 Department of Psychiatry, VU University Medical Center, Amsterdam, the Netherlands; 16 MRC Integrative Epidemiology Unit, University of Bristol, Bristol, UK; 17 Bristol Medical School, University of Bristol, Bristol, United Kingdom; 18 Institute for Bioinnovation, Biomedical Sciences Research Center Al. Fleming, Vari, Greece; 19 Department of Medicine, Imperial College London, London, UK; 20 Cardiovascular Medicine Unit, Center for Molecular Medicine, Department of Medicine, Karolinska Institutet, Stockholm, Karolinska University Hospital, Solna, Sweden; 21 Institute for Molecular Bioscience, The University of Queensland, Brisbane, QLD 4072 Australia; 22 Department of Epidemiology Research, Statens Serum Institut, Copenhagen, Denmark; 23 Université de Lorraine, IGE-PCV, F-54000 Nancy, France; 24 Genetic Epidemiology Unit, Department of Epidemiology, Erasmus Medical Center, Rotterdam, the Netherlands; 25 CARIM School for Cardiovascular Diseases and Maastricht Centre for Systems Biology (MaCSBio, Maastricht University, Maastricht, the Netherlands; 26 Department of Physiology, Maastricht University, Maastricht, the Netherlands; 27 MRC Human Genetics Unit, MRC Institute of Genetics and Molecular Medicine, University of Edinburgh, Western General Hospital, Edinburgh, UK; 28 Translational Gerontology Branch, Longitudinal Study Section, National Institute on Aging, Baltimore, MD USA; 29 Department of Biostatistics and Center for Statistical Genetics, University of Michigan, Ann Arbor, MI USA; 30 Division of Endocrinology, Diabetes, and Nutrition, Department of Medicine, University of Maryland, School of Medicine, Baltimore, MD USA; 31 Department of Epidemiology, School of Public Health, University of Michigan, Ann Arbor, MI USA; 32 Laboratory for Cancer Medicine, Harry Perkins Institute of Medical Research, University of Western Australia Centre for Medical Research, Nedlands, WA Australia; 33 Broad Institute of Harvard and Massachusetts Institute of Technology (MIT), Cambridge, MA USA; 34 Center for Human Genetic Research, Massachusetts General Hospital, Boston, MA USA; 35 Department of Genetics, Harvard Medical School, Boston, MA USA; 36 Departmentartment of Anesthesia, Critical Care and Pain Medicine, MGH, Boston, MA USA; 37 Department of Biostatistics and Data Science, Division of Public Health Sciences, Wake Forest University School of Medicine, Winston-Salem, NC USA; 38 Public Health Genomics Unit, Department of Chronic Disease Prevention, Finnish Institute for Health and Welfare, Helsinki, Finland; 39 Wellcome Trust Sanger Institute, Wellcome Trust Genome Campus, Hinxton, UK; 40 Department of Epidemiology Erasmus MC, Rotterdam, the Netherlands; 41 Icelandic Heart Association, Kopavogur, Iceland; 42 Faculty of Medicine, University of Iceland, Reykjavik, Iceland; 43 Cardiovascular Health Research Unit, University of Washington, Seattle, WA USA; 44 Department of Medicine, University of Washington, Seattle, WA USA; 45 Department of Cardiology, University of Groningen, University Medical Center Groningen, Groningen, The Netherlands; 46 Cardiovascular Medicine, Radcliffe Department of Medicine, University of Oxford, Oxford, UK; 47 Robertson Centre for Biostatistics, University of Glasgow, Glasgow, UK; 48 Institute of Biodiversity, Animal Health & Comparative Medicine, University of Glasgow, Glasgow, UK; 49 William Harvey Research Institute, Barts and The London School of Medicine and Dentistry, Queen Mary University of London, London, UK; 50 NIHR Barts Cardiovascular Biomedical Research Unit, Barts and The London School of Medicine and Dentistry, Queen Mary University of London, London, UK; 51 Department of Medical Genetics, University of Lausanne, Lausanne, Switzerland; 52 deCODE Genetics, Reykjavik, Iceland; 53 Cardiovascular Medicine Unit, Department of Medicine, Solna, Karolinska Institutet, Stockholm, Sweden; 54 Center for Molecular Medicine, Karolinska University Hospital Solna, Stockholm, Sweden; 55 Institute of Health and Wellbeing, University of Glasgow, Glasgow, UK; 56 Department of Preventive Medicine, Northwestern University Feinberg School of Medicine, Chicago, IL USA; 57 Department of Medical Sciences, Molecular Epidemiology and Science for Life Laboratory, Uppsala University, Uppsala, Sweden; 58 Usher Institute, University of Edinburgh, Edinburgh, UK; 59 Synpromics Ltd, Roslin Innovation Centre, Easter Bush Campus, Edinburgh, EH25 9RG UK; 60 Wellcome Trust Sanger Institute, Hinxton, UK; 61 Department of Public Health and Caring Sciences, Uppsala Universitet, Uppsala, Sweden; 62 Vth Department of Medicine, Medical Faculty Mannheim, Heidelberg University, Mannheim, Germany; 63 Division of Angiology, Department of Internal Medicine, Medical University of Graz, Graz, Austria; 64 Institute of Genetic Epidemiology,Helmholtz Zentrum München, German Research Center for Environmental Health, Neuherberg, Germany; 65 Institute of Medical Informatics, Biometry and Epidemiology, Chair of Epidemiology and Chair of Genetic Epidemiology, Ludwig-Maximilians-Universität, Munich, Germany; 66 Department of Medicine I, University Hospital Grosshadern, Ludwig-Maximilians-University, Munich, Germany; 67 Institute of Medical Biostatistics, Epidemiology and Informatics (IMBEI, University Medical Center, Johannes Gutenberg University, 55101 Mainz, Germany; 68 Analytic and Translational Genetics Unit, Massachusetts General Hospital, Boston, MA USA; 69 Program in Medical and Population Genetics, Broad Institute of MIT and Harvard, Cambridge, MA USA; 70 Stanley Center for Psychiatric Research, Broad Institute of MIT and Harvard, Cambridge, MA USA; 71 Institute for Molecular Medicine Finland, FIMM, University of Helsinki, Helsinki, Finland; 72 Public Health Genomics Unit, Finnish Institute for Health and Welfare, Helsinki, Finland; 73 Department of Public Health & Clinical Medicine, Umeå University, Umeå, Sweden; 74 Department of Clinical Sciences, Genetic and Molecular Epidemiology Unit, Skåne University Hospital Malmö, Malmö, Sweden; 75 Department of Odontology, Umeå University, Umeå, Sweden; 76 Oxford Centre for Diabetes, Endocrinology and Metabolism, University of Oxford, Oxford, UK; 77 RIKEN, Center for Integrative Medical Sciences, Laboratory for Endocrinology, Metabolism and Kidney Disease, Yokohama, Japan; 78 Division of Statistical Genomics, Washington University School of Medicine, St. Louis, MO USA; 79 Istituto di Ricerca Genetica e Biomedica, CNR, Monserrato, Italy; 80 Department of Genetics, University Medical Center Groningen, University of Groningen, Groningen, the Netherlands; 81 Department of Medicine, University of Leipzig, Leipzig, Germany; 82 IFB AdiposityDiseases, University of Leipzig, Leipzig, Germany; 83 Department of Epidemiology, University of Groningen, University Medical Center Groningen, Groningen, the Netherlands; 84 Faculty of Medicine, University of Split, Split, Croatia; 85 Department of Clinical Epidemiology and Biostatistics, Academic Medical Center, University of Amsterdam, Amsterdam, the Netherlands; 86 Laboratory of Clinical Investigation, National Institute of Aging, Baltimore, MD USA; 87 Pediatric Research Center, Department of Women’s & Child Health, University of Leipzig, Leipzig, Germany; 88 Department of Medicine, University of Eastern Finland and Kuopio University Hospital, Kuopio, Finland; 89 Department of Dietetics-Nutrition, Harokopio University, Athens, Greece; 90 Center for Biomedicine, European Academy Bozen/Bolzano (EURAC) (Affiliated Institute of the University of LübeckLübeckGermany), Bolzano, Italy; 91 Kuopio Research Institute of Exercise Medicine, Kuopio, Finland; 92 Department of Clinical Physiology and Nuclear Medicine, Kuopio University Hospital, Kuopio, Finland; 93 Geriatric Unit, Azienda Sanitaria Firenze (ASF), Florence, Italy; 94 Institute of Epidemiology, Helmholtz Zentrum München, German Research Center for Environmental Health, Neuherberg, Germany; 95 Department of Epidemiology and Prevention, Division of Public Health Sciences, Wake Forest University School of Medicine, Winston-Salem, NC USA; 96 Department of Epidemiology, Center for Aging and Population Health, University of Pittsburgh, Pittsburgh, PA USA; 97 Department of Medical Sciences, Uppsala University, Akademiska sjukhuset, Uppsala, Sweden; 98 Pat McPherson Centre for Pharmacogenetics and Pharmacogenomics, Division of Molecular and Clinical Medicine, Ninewells Hospital and Medical School, University of Dundee, Dundee, UK; 99 Estonian Genome Center, University of Tartu, Tartu, Estonia; 100 Department of Epidemiology and Public Health, University College London, London, UK; 101 University of Essex, Wivenhoe Park, Colchester, Essex UK; 102 Institute of Clinical Diabetology, German Diabetes Center, Leibniz Center for Diabetes Research at Heinrich Heine University Düsseldorf, Düsseldorf, Germany; 103 Division of Endocrinology and Diabetology, Medical Faculty, Heinrich Heine University Düsseldorf, Düsseldorf, Germany; 104 Department of Public Health Solutions, Finnish Institute for Health and Welfare, P.O. Box 30, Helsinki, FI-00271 Finland; 105 Department of Medicine, University of Helsinki and Helsinki University Central Hospital, P.O. Box 340, Haartmaninkatu 4, Helsinki, FI-00029 Finland; 106 Minerva Foundation Institute for Medical Research, Biomedicum 2U, Tukholmankatu 8, Helsinki, FI-00290 Finland; 107 Division of Cardiovascular Epidemiology, Institute of Environmental Medicine, Karolinska Institutet, Stockholm, Sweden; 108 Department of Internal Medicine, University of Groningen, University Medical Center Groningen, Groningen, the Netherlands; 109 Institute of Public Health and Clinical Nutrition, University of Eastern Finland, Kuopio, Finland; 110 Dept of Cardiology, Leiden University Medical Center, Leiden, the Netherlands; 111 Netherlands Heart Institute, Utrecht, the Netherlands; 112 Institute of Cardiovascular and Medical Sciences, University of Glasgow, Glasgow, United Kingdom; 113 Department of Government Services, Finnish Institute for Health and Welfare, Helsinki, Finland; 114 PEDEGO Research Unit, Medical Research Center Oulu, Oulu University Hospital and University of Oulu, Oulu, Finland; 115 IMM Center for Human Genetics, University of Texas Health Science Center at Houston, Houston, TX USA; 116 Division of Epidemiology, School of Public Health, University of Texas Health Science Center at Houston, Houston, TX USA; 117 Division of Epidemiology and Community Health, School of Public Health, University of Minnesota, Minneapolis, MiI USA; 118 Department of Medical Epidemiology and Biostatistics, Karolinska Institutet, Stockholm, Sweden; 119 Department of Clinical Sciences, Diabetes and Endocrinology Research Unit, University Hospital Malmö, Lund University, Malmö, Sweden; 120 Department of Endocrinology, University of Groningen, University Medical Center Groningen, Groningen, the Netherlands; 121 General Medicine Division, Massachusetts General Hospital, Boston, MA USA; 122 Department of Medicine, Harvard Medical School, Boston, MA USA; 123 Department of Vascular Medicine, Amsterdam UMC, Amsterdam, the Netherlands; 124 Novo Nordisk A/S, Copenhagen, Denmark; 125 Finnish Institute for Health and Welfare, Diabetes Prevention Unit, Helsinki, Finland; 126 Department of Medicine, Division for Prevention and Care of Diabetes, Faculty of Medicine Carl Gustav Carus, Technische Universität Dresden, Dresden, Germany; 127 Department for Prevention and Care of Diabetes, Faculty of Medicine Carl Gustav Carus, Technische Universität Dresden, Dresden, Germany; 128 Paul Langerhans Institute Dresden of the Helmholtz Center Munich at University Hospital and Faculty of Medicine, TU Dresden, Dresden, Germany; 129 German Center for Diabetes Research (DZD e.V.), Neuherberg, Germany; 130 Cardiology Group, Frankfurt-Sachsenhausen, Germany; 131 Lee Kong Chian School of Medicine, Nanyang Technological University Singapore and Imperial College London, Singapore, Singapore; 132 Synlab Academy, Synlab Holding Deutschland GmbH, Mannheim, Germany; 133 Princess Al-Jawhara Al-Brahim Centre of Excellence in Research of Hereditary Disorders (PACER-HD, King Abdulaziz University, Jeddah, Saudi Arabia; 134 Institute of Biomedicine/Physiology, University of Eastern Finland, Kuopio Campus, Kuopio, Finland; 135 Faculty of Medicine, Center for Life Course Health Research, University of Oulu, Oulu, Finland; 136 Unit of General Practice, Oulu University Hospital, Oulu, Finland; 137 Finnish Diabetes Association, Tampere, Finland; 138 Pirkanmaa Hospital District, Tampere, Finland; 139 Diabetes and Obesity Research Institute, Cedars-Sinai Medical Center, Los Angeles, CA USA; 140 Department of Chronic Disease Prevention, Finnish Institute for Health and Welfare, Helsinki, Finland; 141 Department of Public Health, University of Helsinki, Helsinki, Finland; 142 Centre for Vascular Prevention, Danube-University Krems, Krems, Austria; 143 Diabetes Research Group, King Abdulaziz University, Jeddah, Saudi Arabia; 144 Department of Nutrition, Harvard School of Public Health, Boston, MA USA; 145 Department of Public Health & Clinical Medicine, Units of Medicine and Nutritional Research, Umeå University, Umeå, Sweden; 146 Inserm, CESP Center for Research in Epidemiology and Public Health, U1018 Villejuif, France; 147 Univ Paris-Saclay, Univ Paris Sud, UVSQ, UMRS 1018, UMRS 1018, Villejuif, France; 148 Centre for Medical Systems Biology, Leiden, the Netherlands; 149 Hannover Unified Biobank, Hannover Medical School, Hannover, Germany; 150 Institute of Human Genetics, Hannover Medical School, Hannover, Germany; 151 Clinical Research Branch, National Institute on Aging, Baltimore, Maryland USA; 152 Department of Neurology, General Central Hospital, Bolzano, Italy; 153 Department of Neurology, University of Lübeck, Lübeck, Germany; 154 Genetics of Complex Traits, Peninsula Medical School, University of Exeter, Exeter, UK; 155 The Regeneron Genetics Center, Regeneron Pharmaceuticals, Tarrytown, NY USA; 156 School of Public Health, University of Adelaide, Adelaide, Australia; 157 U872 Institut National de la Santé et de la Recherche Médicale, Centre de Recherche des Cordeliers, 75006 Paris, France; 158 Geriatric Epidemiology Section, Laboratory of Epidemiology, Demography, and Biometry, National Institute on Aging, Bethesda, MD USA; 159 Department of Genetics, University Medical Center Groningen, University of Groningen, Groningen, the Netherlands; 160 MRC Integrative Epidemiology Unit (IEU), University of Bristol, Bristol, UK; 161 The Charles Bronfman Institute for Personalized Medicine, Icahn School of Medicine at Mount Sinai, New York, NY USA; 162 Finnish Institute for Health and Welfare, Helsinki, Finland; 163 Endocrinology, Abdominal Centre, University of Helsinki and Helsinki University Hospital, Helsinki, Finland; 164 Research Program for Clinical and Molecular Metabolism, University of Helsinki and Folkhälsan Research Center, Helsinki, Finland; 165 Netherlands Consortium for healthy ageing, the Hague, the Netherlands; 166 Department of Epidemiology, Harvard T.H. Chan School of Public Health, Boston, USA; 167 Faculty of Medicine University of Iceland, Reykjavik, Iceland; 168 Department of Epidemiology, University of Washington, Seattle, WA USA; 169 Department of Medicine, University Hospital Lausanne, Lausanne, Switzerland; 170 Cardiothoracic Surgery Unit, Department of Molecular Medicine and Surgery, Karolinska Institutet, Stockholm, Sweden; 171 Department of Epidemiology and Biostatistics and HPA-MRC Center, School of Public Health, Imperial College London, London, UK; 172 Institue of Health Sciences, University of Oulu, Oulu, Finland; 173 Faculty of Medicine, University of Iceland, Reykjavík, Iceland; 174 Department of Cardiology, Karolinska University Hospital Solna, Stockholm, Sweden; 175 Department of Statistics, University of Oxford, Oxford, UK; 176 Oxford National Institute for Health Research Biomedical Research Centre, Churchill Hospital, Oxford, UK; 177 Department of Genetic Medicine and Development, University of Geneva Medical School, Geneva, Switzerland; 178 Big Data Institute, Li Ka Shing Centre for Health Information and Discovery, University of Oxford, Oxford, UK; 179 School of Biosciences and Medicine, Department of Clinical and Experimental Medicine, University of Surrey, Guildford, UK; 180 Department of Clinical Science, University of Bergen, Bergen, Norway; 181 Department of Preventive Medicine, Keck School of Medicine of USC, Los Angeles, CA USA; 182 Department of Physiology & Neuroscience, Keck School of Medicine of USC, Los Angeles, CA USA; 183 USC Diabetes and Obesity Research Institute, Los Angeles, CA USA; 184 Department of Medicine, Division of Cardiovascular Medicine, Stanford University School of Medicine, Stanford, CA USA; 185 Stanford Cardiovascular Institute, Stanford University, Stanford, CA 94305 USA; 186 Diabetes Research Center, Diabetes Unit, Massachusetts General Hospital, Boston, MA USA; 187 Department of Biostatistics, Boston University School of Public Health, Boston, MA USA; 188 University of Cambridge Metabolic Research Laboratories and NIHR Cambridge Biomedical Research Centre, Wellcome Trust-MRC Institute of Metabolic Science, Cambridge, UK; 189 Department of Biostatistics, University of Liverpool, Liverpool, UK; 190 Centre for Genetics and Genomics Versus Arthritis, Centre for Musculoskeletal Research, The University of Manchester, Manchester, UK; 191 Institute of Biochemistry and Genetics, Ufa Federal Research Centre Russian Academy of Sciences, Ufa, Russian Federation; 192 Present Address: Genentech, 340 Point San Bruno Boulevard, South San Francisco, CA 94080 USA; 193 Present Address: Exeter Centre of ExcEllence in Diabetes (ExCEED), University of Exeter Medical School, Exeter, UK

**Keywords:** Genome-wide association studies, Quantitative trait, Diagnostic markers, Pre-diabetes, Type 2 diabetes

## Abstract

Differences between sexes contribute to variation in the levels of fasting glucose and insulin. Epidemiological studies established a higher prevalence of impaired fasting glucose in men and impaired glucose tolerance in women, however, the genetic component underlying this phenomenon is not established. We assess sex-dimorphic (73,089/50,404 women and 67,506/47,806 men) and sex-combined (151,188/105,056 individuals) fasting glucose/fasting insulin genetic effects via genome-wide association study meta-analyses in individuals of European descent without diabetes. Here we report sex dimorphism in allelic effects on fasting insulin at *IRS1* and *ZNF12* loci, the latter showing higher RNA expression in whole blood in women compared to men. We also observe sex-homogeneous effects on fasting glucose at seven novel loci. Fasting insulin in women shows stronger genetic correlations than in men with waist-to-hip ratio and anorexia nervosa. Furthermore, waist-to-hip ratio is causally related to insulin resistance in women, but not in men. These results position dissection of metabolic and glycemic health sex dimorphism as a steppingstone for understanding differences in genetic effects between women and men in related phenotypes.

## Abstract (web summary)

Sex differences in fasting glucose and insulin have been identified, but the genetic loci underlying these differences have not. Here, the authors perform a meta-analysis of genome-wide association studies to detect sex-specific and sex-dimorphic loci associated with fasting glucose and insulin.

## Introduction

There are established differences between sexes in insulin resistance and blood glucose levels^1^. In general, men are more insulin resistant and have higher levels of fasting glucose (FG) as defined by impaired fasting glycaemia (FG concentration 5.6–6.9 mmol/l), whereas women are more likely than men to have elevated 2-h glucose concentrations (impaired glucose tolerance, IGT, i.e., 2-h post-challenge glucose concentration 7.8–11 mmol/l) with both measures defining categories of individuals at higher diabetes risk^1–3^. Diverse biological, cultural, lifestyle, and environmental factors contribute to the relationship between sex dimorphism of early changes in glucose homeostasis and type 2 diabetes (T2D) pathogenesis^4,5^. These observations raise hypotheses about a role for the genetic mechanisms underlying sex differences in the maintenance of glucose homeostasis as measured by FG and fasting insulin (FI).

Genome-wide association studies (GWAS) have thus far been instrumental in the identification of dozens of FG/FI loci through large-scale meta-analyses^6,7^. Despite the success of GWAS efforts, men and women have typically been analyzed together in sex-combined analyses, with sex used as a covariate in the model to account for marginal differences on traits between them. Sex-combined analyses assume homogeneity of the allelic effects in men and women, and therefore are sub-optimal in the presence of heterogeneity in genetic effects by sex, i.e., sex-dimorphic effects.

Recently, several large-scale GWAS meta-analyses in European descent individuals have identified genetically encoded sex dimorphism for metabolic traits and outcomes, including female-specific effects on central obesity^8–11^, T2D^12^, and diabetic kidney disease^13^. Only one female-specific association with FG has been reported at *COL26A1* (*EMID2*) in a relatively small study of European descent individuals^7^. The large population-based UK Biobank (www.ukbiobank.ac.uk), a potential natural target for exploring sex dimorphism in glycemic trait variability, did not collect fasting state samples and, therefore, could not be considered for such an analysis. Unraveling the heterogeneity in genetic effects on the regulation of glycemic trait variability and T2D risk may prove useful for personalized approaches for preventative and disease treatment measures tailored specifically to women or men. Moreover, the meta-analysis of female- and male-specific GWAS allowing for sex-heterogeneity in allelic effects, while requiring an additional degree of freedom (df), can lead to a substantial gain in power over the usual sex-combined test of association when effects are not homogeneous across men and women^14,15^.

Here we evaluate sex-specific, sex-dimorphic, and sex-homogeneous effects in FG/FI GWAS from individuals of European descent without diabetes within the Meta-Analyses of Glucose and Insulin-related traits Consortium (MAGIC). Our aims are threefold: (1) to explore sex-dimorphic effects on fasting glycemic traits at established FG/FI loci; (2) to discover FG/FI biology and loci based on modeling heterogeneity between sexes and through sex-combined analyses; and (3) to evaluate, through simulations, the power of sex-specific/-combined/-dimorphic analyses to detect variants associated with quantitative traits over a range of models of heterogeneity, given the current sample size in MAGIC. We show sex-dimorphism in allelic effects on FI at *IRS1* and *ZNF12* loci. In addition, we report sex-homogeneous effects on FG at seven novel loci. Our analyses show stronger genetic correlations in women than in men between FI and two traits, waist-to-hip ratio (WHR) and anorexia nervosa. Furthermore, we show that WHR is causally related to insulin resistance in women, but not in men. Finally, our simulation study highlights that, given the current sample size, the 2-df sex-dimorphic test is more powerful, compared to the sex-combined approach, when causal variants have allelic effects specific to one sex and in the presence of heterogeneous allelic effects in men and women. When the allelic effects of the causal variant are similar between men and women, the sex-combined test is only slightly more powerful than the sex-dimorphic approach, especially for causal variant effect allele frequency (CAF) ≤ 0.1. However, under the scenarios of effects that are larger in one sex than the other or specific to just one sex, the heterogeneity test is generally underpowered.

## Results

### Sex-dimorphic and sex-combined meta-analyses for FG/FI

We obtained FG/FI sex-specific results for up to 73,089/50,404 women and 67,506/47,806 men from population-based studies; sex-combined meta-analyses for these traits additionally included 13,613 individuals from four family-based studies. All studies were of European ancestry, and were based on GWAS imputed to the HapMap II CEU reference panel^16^ or Metabochip array data^17^ (Supplementary Data 1). We further improved the genetic variant genome-wide coverage by imputing the summary statistics of FG/FI sex-dimorphic and sex-combined meta-analyses to 1000 Genomes Project density using the SS-imp software (“Methods”)^18^. We investigated the sex-dimorphic and homogeneous effects of 8.7 million autosomal single-nucleotide polymorphisms (SNPs) on FG/FI under an additive genetic model. In the sex-dimorphic meta-analysis, we allowed for heterogeneity in allelic effects between women and men (2-df test) (“Methods”). We evaluated the evidence for heterogeneity of allelic effects between sexes using Cochran’s Q-statistic^14,15^ (Supplementary Data 2 and 3).

### Sex-dimorphic effects at established FG/FI loci

To define the extent of sex-dimorphic effects, we evaluated sex heterogeneity at 36/19 established FG/FI loci^6^ (Supplementary Data 2 and 3). Although not reaching the statistical significance after Bonferroni correction for multiple testing (*P*_heterogeneity_ ≤ 0.0014 for FG with 36 variants and *P*_heterogeneity_ ≤ 0.0026 for FI with 19 variants), we observed suggestive evidence for heterogeneity at *IRS1*, where variant rs2943645 was associated with FI in men only (*β*_male_ = 0.022, *P*_male_ = 1.0 × 10^−8^, *P*_sex-dimorphic_ = 1.0 × 10^−8^) with differences in allelic effects by sex (Δ*β*_(βmale–βfemale)_ = 0.015, *P*_heterogeneity_ = 0.0053) (Supplementary Data 3, Supplementary Fig. 1a, b). The male-specific effects on FI variability were consistent with previously reported effects specific to men on percentage of body fat and lipids at the *IRS1* locus^10^. In addition, we observed nominal evidence for heterogeneity at *COBLL1*/*GRB14* (rs10195252, *P*_heterogeneity_ = 0.039) with more pronounced effects on FI in women (*β*_female_ = 0.018, *P*_female_ = 1.2 × 10^−6^, *P*_sex-dimorphic_ = 1.5 × 10^−6^) than men (*β*_male_ = 0.007, *P*_male_ = 0.073) (Supplementary Data 3). Our observations were consistent with previous reports of effects at *COBLL1*/*GRB14* specific to women on WHR^8,9,11^ and triglycerides^19^. Four established FG loci, *PROX1*, *ADCY5*, *PCSK1*, and *SLC30A8*, showed larger effects in women with nominal evidence for sex heterogeneity (Supplementary Data 2). We did not observe association at the previously reported female-specific FG locus *COL26A1* (*EMID2*) (rs6961305, r^2^_EUR_ = 0.89 with reported SNP rs6947345, *P*_sex-combined_ = 0.199, *P*_sex-dimorphic_ = 0.035)^7^.

**Fig. 1:**
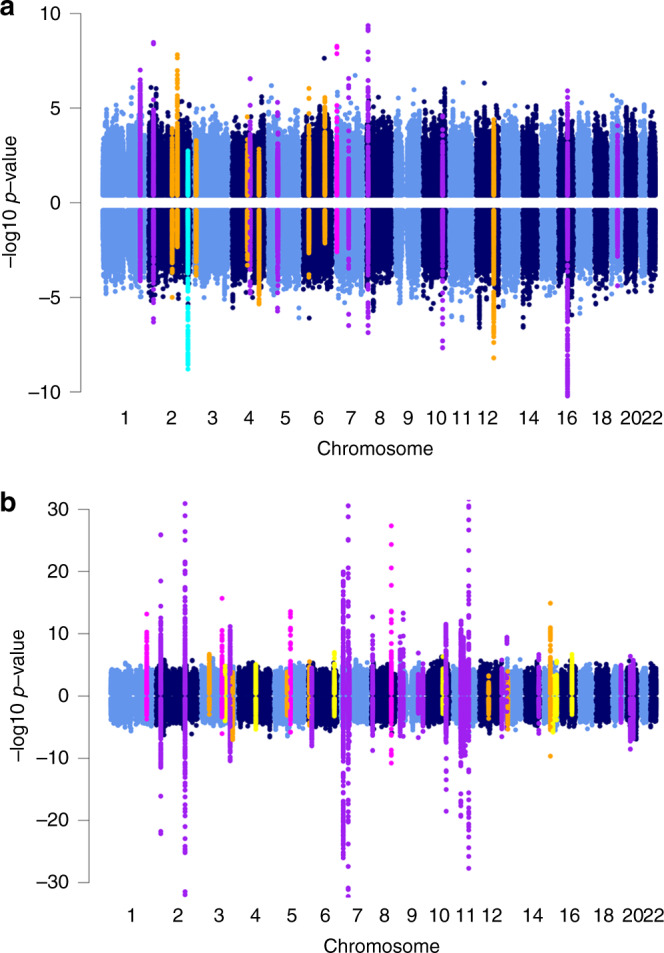
Miami plots of sex-specific associations. **a** FI sex-specific associations, **b** FG sex-specific associations showing women on upper panel (all *y* axis values are positive) and men on lower panel (all *x* axis values are negative). Established or novel loci with sex-dimorphic effects (*P*_sex-dimorphic_ ≤ 5.0 × 10^−8^) and nominal sex heterogeneity (*P*_heterogeneity_ < 0.05) are shown in magenta (larger effect in women) or cyan (larger effect in men). Novel genome-wide significant loci from sex-combined analyses with sex-homogeneous effects (*P*_sex-combined_ ≤ 5.0 × 10^−8^) are shown in yellow. Established loci reaching genome-wide significance in sex-combined analyses and showing no sex heterogeneity (*P*_heterogeneity_ > 0.05) are colored in purple. All remaining established loci (i.e. no significant sex-dimorphic or sex-homogeneous effects) are marked in orange.

### Novel loci with sex-dimorphic and -combined FG/FI effects

To discover FG/FI loci based on modeling heterogeneity and through sex-combined analyses, we required that the lead SNP was genome-wide significant in the 2df sex-dimorphic or in the 1df sex-combined test of association (*P* ≤ 5 × 10^−8^)^14^. We considered SNPs to be novel if they were not in linkage disequilibrium (LD, HapMap CEU/1000 genomes EUR: r^2^ < 0.01) with any variant already known to be associated with the trait and located more than 500 kb away from any previously reported lead SNP (Fig. 1).

**Fig. 2:**
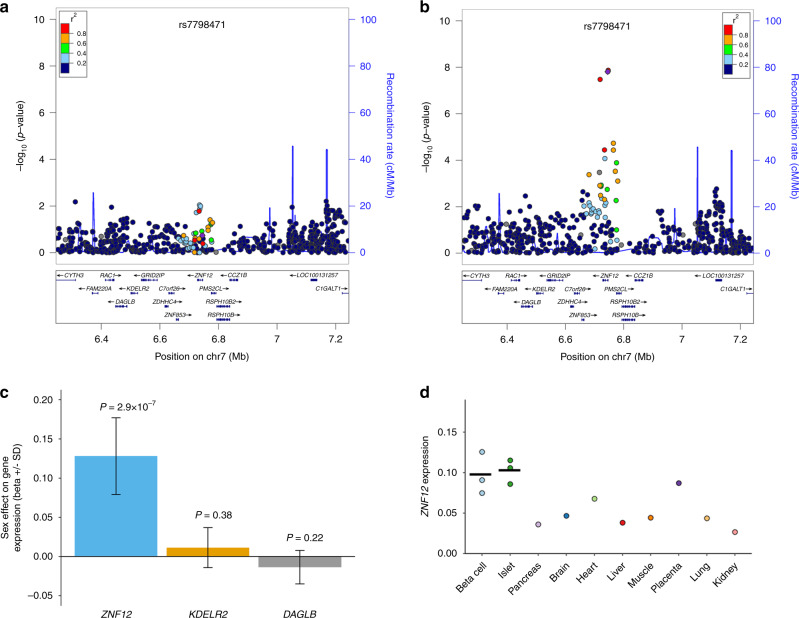
Plots for *ZNF12* locus with sex-dimorphic effects on FI. **a** female-specific regional plot, **b** male-specific regional plot, **c** *ZNF12* whole blood RNA expression data in *n* = 3,621 Netherlands Twin Register and Netherlands Study of Anxiety and Depression studies. Beta ± SD (error bars) represent the sex effect in the linear regression analysis where the average gene expression by all probes in the gene was predicted by sex, as well as the following covariates: age, smoking status, RNA quality, hemoglobin, study, time of blood sampling, month of blood sampling, time between blood sampling and RNA extraction, and the time between RNA extraction and RNA amplification. A positive value represents an upregulated expression in women and a negative value an upregulated expression in men. The *P* value represents the significance of sex effect from the linear models (*P* values are not corrected for multiple testing). **d** *ZNF12* tissue expression relative to three housekeeping genes (*PPIA*, *B2M*, and *HPRT*). For beta cell (*n* = 3) and islets (*n* = 3) data, lines are means. Quantitative RT-PCR was carried out using cDNAs from three human donors (beta-cells and islets). The other tissues were commercial cDNAs (one point observation).

We detected a sex-dimorphic effect on higher FI levels within the first intron of *ZNF12* at rs7798471-C (*P*_sex-dimorphic_ = 4.5 × 10^−8^), which has not been previously associated with any glycemic or other metabolic trait. We observed nominal evidence of sex heterogeneity (*P*_heterogeneity_ = 0.0046) with detectable effects only in women (β_female_ = 0.026, *P*_female_ = 1.5 × 10^−8^; β_male_ = 0.007, *P*_male_ = 0.18) (Table 1 and Fig. 2a, b). The sex-combined analysis at the same variant did not reach genome-wide significance (*P*_*sex-*combined_ = 2.4 × 10^−7^) (Supplementary Data 4). This signal was not associated with T2D (*P* > 0.05)^20^, but was previously nominally associated in the same direction with FI^21^. In addition, a proxy variant on Metabochip (rs3801033, *r*^2^_EUR_ = 0.87 with rs7798471) was nominally associated with FI^22^ in a previous sex-combined meta-analysis. Furthermore, the FI increasing allele (C) at rs7798471 was previously associated with higher body-mass index (BMI) in GIANT UK Biobank GWAS with stronger effects observed in women than men^23^. For FG, SNP rs1281962 located in the first intron of the *RGS17* gene revealed larger effects on FG in women (*β*_female_ = 0.014, *P*_female_ = 2.6 × 10^−7^) than in men at nominal significance (*P*_sex-dimorphic_ = 2.2 × 10^−7^, *P*_heterogeneity_ = 0.042) (Supplementary Data 4, Supplementary Fig. 1c–e). The FG-increasing allele at *RGS17* was associated with higher BMI in GIANT UK Biobank GWAS with larger effects in women than men^23^.

**Table 1:** Novel genetic loci exerting genome-wide significant sex-dimorphic or sex-homogeneous effects on FI/FG in individuals without diabetes.

Primary trait	SNP	Chr:Pos	Nearest gene	Alleles (effect/other)	EAF	Analysis	FG effect (SE)	FG *P*	FG N	FI effect (SE)	FI *P*	FI *N*
FI	rs7798471	7:6744957	*ZNF12*	C/T	0.273	Sex-specific (men)	0.0026 (0.0040)	0.517	43,868	0.0067 (0.0051)	0.182	29,394
						Sex-specific (women)	0.0063 (0.0036)	0.082	51,424	0.0262 (0.0046)	**1.55** **×** **10**^**−8**^	34,987
						Sex-dimorphic		0.178			**4.54** **×** **10**^**−8**^	
						Sex heterogeneity		0.493			4.6 × 10^−3^	
FG	rs11919595	3:142617816	*ZBTB38*	T/C	0.919	Sex-combined	0.0248 (0.0043)	**9.75** **×** **10**^**−9**^	138,567	−0.0022 (0.0053)	0.684	100,922
FG	rs223486	4:103684953	*MANBA*, *UBE2D3*	C/G	0.507	Sex-combined	0.0135 (0.0025)	**3.92** **×** **10**^**−8**^	91,405	−0.0002 (0.0029)	0.954	65,353
FG	rs1281962	6:153431376	*RGS17*	C/G	0.538	Sex-combined	0.0106 (0.0019)	**3.61** **×** **10**^**−8**^	151,151	0.0015 (0.0023)	0.525	104,730
FG	rs2785137	10:95386207	*PDE6C*	G/A	0.649	Sex-combined	0.0117 (0.0021)	**4.97** **×** **10**^**−8**^	136,750	−0.0027 (0.0025)	0.282	99,243
FG	rs7178572	15:77747190	*HMG20A*	G/A	0.688	Sex-combined	0.0119 (0.0021)	**2.70** **×** **10**^**−8**^	138,579	0.0020 (0.0025)	0.443	100,920
FG	rs6598541	15:99271135	*IGF1R*	A/G	0.362	Sex-combined	0.0121 (0.0021)	**1.04** **×** **10**^**−8**^	138,505	0.0063 (0.0025)	0.013	100,921
FG	rs8044995	16:68189340	*NFATC3*	G/A	0.836	Sex-combined	0.0162 (0.0028)	**5.76** **×** **10**^**−9**^	127,333	−0.0022 (0.0034)	0.521	90,483

EAF: allele frequency of the primary trait (FG or FI) raising allele from the sex-combined meta-analyses. Per allele effect (SE) for FI represents changes of natural-log transformed levels of this trait. Sex heterogeneity represents the differences in allelic effects between sexes. The Cochran’s *Q* test (for sex heterogeneity) *P* value is also shown. Significant *P* values (*P*_sex-dimorphic_ < 5 × 10^−8^, *P*_sex-combined_ < 5 × 10^−8^) are highlighted in bold.

*FG* fasting glucose, *FI* fasting insulin, *Chr* chromosome, *Pos* Position GRCh37.

In the sex-combined meta-analyses that included four additional family-based studies compared to the sex-dimorphic meta-analyses, we identified genome-wide significant associations for FG at six novel loci (*ZBTB38*, *MANBA*/*UBE2D3*, *RGS17*, *PDE6C, IGF1R,* and *NFATC3*) and one established T2D locus (*HMG20A*, same variant)^24^ (Table 1, Fig. 1, Supplementary Fig. 2). These loci have not been associated with FG in a previously published meta-analysis likely due to smaller sample sizes (Supplementary Data 5)^22^. We evaluated the effects of these loci on T2D in a large-scale European ancestry GWAS meta-analysis, and only the variant at *ZBTB38* was nominally associated with T2D (*P* = 0.0080), further supporting only partial overlap between genetic variation influencing glucose levels and T2D risk^6^.

The variant rs2785137 at *PDE6C*, although nearby the two previously reported T2D variants at the *HHEX* locus, is an independent signal (rs1111875, *r*^2^_EUR_ ≤ 0.01 and rs5015480, *r*^2^_EUR_ ≤ 0.01 with rs2785137)^24,25^. The two FG loci, at *IGF1R* (rs6598541) and *NFATC3* (rs8044995), have been previously suggested to contribute to the maintenance of glucose metabolism and/or to insulin response, with the former being also a well-described target in breast cancer^26–28^. The FG-increasing G allele of the *NFATC3* locus lead variant has been also associated with reduced risk of schizophrenia^29^ and lower levels of high-density lipoprotein cholesterol^30^. Interestingly, the lead SNP at the *MANBA/UBE2D3* locus, rs223486, is an intergenic variant located in a region (±500 kb) that harbors several other genes (*CISD2*, *NFKB1*, *SLC9B1/2*, *BDH2* and *CENPE*) (Supplementary Fig. 2b) with reported inflammatory and autoimmune disease associations^31,32^. Two missense variants within *MANBA* (mannosidase, beta A, lysosomal) are in LD (1000 Genomes Project, EUR populations) with our FG lead variant rs223486 [e.g. rs2866413 (p.Thr701Met, *r*^2^ = 0.36) and rs227368 (p.Val253Leu, *r*^2^ = 0.58)] and have suggestive effects on FG (*P*_sex-combined_rs2866413_ = 8.7 × 10^−5^, *P*_sex-combined_rs227368_ = 6.4 × 10^−4^) in the current dataset, but no nominal effect on T2D risk in European ancestry populations (*P*_rs2866413_ = 8.8 × 10^−4^, *P*_rs227368_ = 4.1 × 10^−5^). Approximate conditional analyses using GCTA^33,34^ showed that the rs223486 association with FG was only partially driven by rs228614 variant at the same locus for which previously a significant association with multiple sclerosis has been reported (rs223486, *P*_conditional_rs228614_ = 0.00035, *r*^2^_EUR_ = 0.46) (“Methods”)^31^. Conversely, the rs223486 association with FG was not explained by rs3774959 variant at *MANBA* previously associated with ulcerative colitis (rs223486, *P*_conditional_rs3774959_ = 9.6 × 10^−8^, *r*^2^_EUR_ = 0.12)^32^ (Supplementary Fig. 1f–h), suggesting a genetic relationship between glucose homeostasis and neurodegeneration.

### Sex dimorphism in genetic correlations with other traits

**Fig. 3:**
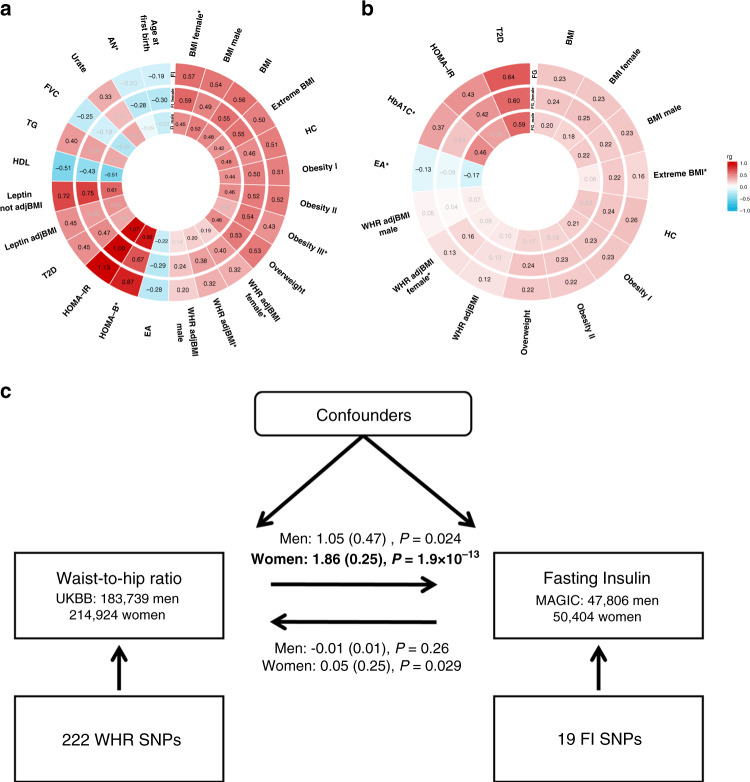
Genetic correlations and causality. **a** Genetic correlations for FI, **b** genetic correlations for FG. Phenotypes with statistically significant (*P* < 0.001) genetic correlations (calculated by LD score regression) with FI/FG in either women or men are plotted. The outer track shows estimates for all together, followed by those for women and men. Traits with *I*^2^ (sex heterogeneity) ≥50% are labeled with asterisks. Gray color indicates traits that do not show significant genetic correlation with the given glycemic trait. Estimates in black color indicate statistically significant associations. **c** bi-directional MR analysis between WHRadjBMI and FI with betas, standard errors of the estimates and *P* values from random-effect inverse-variance weighted regression given for men and women. AN anorexia nervosa, BMI body-mass index, EA educational attainment as of years of schooling 2016, FVC forced vital capacity, HbA1c glycated hemoglobin, HC hip circumference, HDL high-density lipoprotein cholesterol, HOMA-B homeostatic model assessment of beta cell function, HOMA-IR homeostatic model assessment of insulin resistance, leptin adjBMI leptin adjusted for BMI, Leptin not adjBMI leptin not adjusted for BMI, Obesity 1 obesity class 1, Obesity II obesity class II, Obesity III obesity class 3, T2D type 2 diabetes, TG triglycerides, WC waist circumference, WHR adjBMI waist-to-hip ratio adjusted for BMI, UKBB UK Biobank.

We estimated the genetic correlations between FG/FI and 201 traits with sex-combined and sex-specific GWAS summary statistics using LD score regression (“Methods”, Fig. 3a, b). We detected genetic correlations between FI and 22 other traits (*P* < 0.00012, corrected for multiple testing), including obesity-related phenotypes, leptin levels without adjustment for BMI, T2D, high-density lipoprotein cholesterol and triglycerides. Among those, we observed sex heterogeneity in the genetic correlations between FI and two traits: WHR adjusted for BMI (WHRadjBMI) (*r*_gwomen_ = 0.38, *r*_gmen_ = 0.20, *P*_Cochran’s*Q*test_ = 0.015, *I*^2^ = 83%) and WHRadjBMI determined in females only (*r*_gwomen_ = 0.40, *r*_gmen_ = 0.19, *P*_Cochran’s*Q*test_ = 0.0099, *I*^2^ = 85%) (Fig. 3a). Furthermore, estimates for two of these traits were just marginally over the significance threshold for sex heterogeneity in their genetic correlation with FI: anorexia nervosa (*r*_gwomen_ = −0.28, *r*_gmen_ = −0.09, *P*_Cochran’s*Q*test_ = 0.051, *I*^2^ = 74%) and HOMA-B levels (*r*_gwomen_ = 0.67, *r*_gmen_ = 0.92, *P*_Cochran’s*Q*test_ = 0.069, *I*^2^ = 70%) (Supplementary Data 6, Fig. 3a). Analysis of FG yielded statistically significant genetic correlations in both women and men with 13 traits including a number of obesity-related phenotypes, years of schooling, HbA1_c,_ and T2D (Supplementary Data 7, Fig. 3b).

### Sex dimorphism in causal relationship between obesity and FI

Previously, the dissection of causal effects of adiposity, measured through BMI, on FI did not detect sex dimorphism^35^. We applied a bidirectional two-sample Mendelian Randomization (MR) to investigate causality between central obesity, measured through WHRadjBMI, and FI, using WHRadjBMI-associated genetic variants as instrumental variables (“Methods”). Estimates of genetic instruments for WHRadjBMI from the general population were obtained from the UK Biobank (~215,000 women/~184,000 men), while for FI from the present study. We used 222 independent (*r*^2^ < 0.001) SNPs (Supplementary Data 8) that reached genome-wide significance in the sex-combined WHRadjBMI GWAS as instruments and extracted their sex-specific effect on FI, and vice versa for 19 FI SNPs. We observed a significant (*P*_Bonferroni_ < 0.0125, corrected for four tests) causal effect (β_IV-WHRadjBMI_exposure_women_ = 1.86, *P*_IV-WHRadjBMI_exposure_women_ = 1.9 × 10^−13^) of WHRadjBMI on FI in women, but detected no causal effect in the reverse direction (β_IV-FI_exposure_women_ = 0.55, *P*_IV-FI_exposure_women_ = 0.030) nor in men in either direction (*β*_IV-WHRadjBMI_exposure_men_ = 1.05, *P*_IV-WHRadjBMI_exposure_men_ = 0.024; β_IV-FI_exposure_men_ = −0.01, *P*_IV-FI_exposure_men_ = 0.27) (Fig. 3c, Supplementary Data 9) under a random-effect inverse variance weighted model. To further investigate the robustness of the WHRadjBMI-FI causal relationship in women, we assessed the causal effect estimate from the MR-Egger method, which is less sensitive to pleiotropy. The intercept from the MR-Egger regression was estimated to be non-zero (Intercept = −0.002, *P*_Intercept_ = 0.004) for the WHRadjBMI-FI relationship in women, to which a possible explanation is that pleiotropic effects of instrumental variables are not balanced or act randomly. If the non-zero MR-Egger intercept reflects unbalanced pleiotropy and therefore average pleiotropy over all instrumental variants, the slope of the MR-Egger regression provides an unbiased causal estimate. For the WHRadjBMI-FI causal relationship in women, we observed a significant MR-Egger causal estimate (*β*_IV-WHRadjBMI_exposure_women_ = 3.11, *P*_IV-WHRadjBMI_exposure_women_ = 2.4 × 10^−9^) robust to the presence of overall pleiotropy (Supplementary Data 9). We further observed that abdominal fat (defined through waist circumference with adjustment for BMI [WCadjBMI], 222 independent SNPs in women) is the driving factor (*β*_IV-WCadjBMI_exposure_women_ = 0.015, *P*_IV-WCadjBMI_exposure_women_ = 5.3 × 10^−8^) of the WHR causal effect on FI in women. Gluteofemoral fat (defined as hip circumference with adjustment for BMI [HCadjBMI], 274 independent SNPs in women) exerted a moderate inverse causal effect on FI in women (*β*_IV-HCadjBMI_exposure_women_ = −0.01, *P*_IV-HCadjBMI_exposure_women_ = 0.0035. There was no detectable causal effect of WCadjBMI or HCadjBMI on FI in men (*β*_IV-WCadjBMI_exposure_men_ = 0.001, *P*_IV-WCadjBMI_exposure_men_ = 0.81; *β*_IV-HCadjBMI_exposure_men_ = −0.001, *P*_IV-HCadjBMI_exposure_men_ = 072).

### Sex-dimorphic effects on gene expression

We sought to establish whether the sex-dimorphic effects at known FG/FI loci are related to gene expression in a range of tissues. Wherever possible, we evaluated sex-specific/-dimorphic associations using the expression levels in women and men separately. For all expression analyses, we used transcripts of all genes within associated loci with at least nominal evidence for sex heterogeneity (“Methods”). We evaluated sex-dimorphic RNA expression in whole blood from 3,621 individuals from the Netherlands Twin Register (NTR) and Netherlands Study of Anxiety and Depression (NESDA) using the Affymetrix U219 array^36^. We also undertook expression quantitative trait locus (eQTL) analyses in a range of tissues, including gluteal and abdominal fat from the MolOBB study^37^, lymphoblastoid cell lines (LCL) from HapMap 2 participants^38^, as well as liver, heart, aorta adventitia/intima media and mammary artery intima-media from the Advanced Study of Aortic Pathology (ASAP) (“Methods”)^39^. In addition, we investigated gene expression in islets of cadaver donors with IGT compared to those with normal glucose tolerance^40^, as well as in fat, LCLs, and skin tissues from women (MuTHER consortium) (“Methods”)^41^.

In whole blood, we observed nominal evidence of sex-dimorphic effects (representing the significance of the effect of sex in the linear regression analysis, where, after accounting for relevant covariates, the average gene expression was predicted by sex) on RNA expression only for *COBLL1*, where expression in women was higher than in men (*P*_sex_ = 0.047, “Methods”). However, we observed no such sex effects for *GRB14* (*P*_sex_ = 0.93), *IRS1* (*P*_sex_ = 0.16), or genes within other explored loci (Supplementary Data 10). The sex-dimorphic effects on gene expression in other tissues were contradictory and might reflect the relatively small sample sizes available. We observed statistically significant higher expression of *COBLL1* in gluteal fat in women, while in liver *COBLL1* had higher expression in men (Supplementary Data 11). *GRB14* was expressed in fat, LCL, and skin tissue in women, but no expression was observed for *COBLL1* in these tissues (Supplementary Data 11). For *IRS1*, the gene with suggestive evidence of heterogeneity in effects between sexes, we observed higher expression in islets for individuals with IGT compared to those with normal glucose tolerance (Supplementary Data 11, “Methods”).

### Sex-specific functional enrichment of the associations

We performed enrichment analysis of the sex-specific FI and FG results using the GARFIELD software, which integrates features extracted from ENCODE, GENCODE, and Roadmap Epigenomics projects (“Methods”). These analyses suggested significant (*P* < 6.2 × 10^−6^, “Methods”) enrichment peaks for FI in fetal membrane in men but not in women (*P* > 0.05). In addition, for FI, the analyses showed multiple significant enrichment peaks in blood in men, whereas those in women were only nominally significant (*P* = 0.01) (Supplementary Fig. 3a). For FG, we observed significant enrichment in the blood vessel footprints (Supplementary Fig. 3b) and in blood (Supplementary Fig. 3c) only in men.

### Putative biological leads at the novel *ZNF12* FI locus

We scrutinized genes at the FI locus (*ZNF12*) to investigate putative biological leads and links with glucose homeostasis. There are scarce data on the function of *ZNF12*, *KDELR2*, and *DAGLB*, the three genes within this region, which are ubiquitously expressed across human tissues (GTEx consortium)^42^. Therefore, we performed quantitative RT-PCR applied to transcripts from sorted beta cells and isolated pancreatic islets from three human donors, in addition to a commercial panel of human tissues. *ZNF12* was most highly expressed in beta cells and pancreatic islets, which are highly relevant to glucose metabolism (Fig. 2d). *KDELR2* and *DAGLB* were also expressed in sorted beta cells and islets, but showed a relatively higher expression in the placenta (Supplementary Fig. 4). In addition, we explored whole blood array RNA expression for *ZNF12* in NTR and NESDA and we observed large differentiation between sexes with stronger expression in women than men (*P*_sex_ = 2.9 × 10^−7^ in linear regression) (Supplementary Data 10), which was consistent with DNA association analyses (Fig. 2c). No such sex effects on RNA expression were detected for *KDELR2*or *DAGLB* (Supplementary Data 10).

### Power of tests for sex-dimorphic effects through simulations

Our meta-analysis highlighted nominal heterogeneity of the effects on glycemic traits between sexes at several established loci. Therefore, we assessed the power of three types of analyses (sex-combined, sex-specific and 2-df sex-dimorphic) to detect any associations with evidence for sex heterogeneity. More specifically, we tested three scenarios of allelic effects on the two sexes: (1) no heterogeneity between the two sexes; (2) effects on both sexes with the presence of heterogeneity between them; and (3) effect specific to one sex only, where we used women as an example. Within each scenario, we evaluated a range of CAF (ranging from 0.05 to 0.5) and effect sizes (ranging from 0 to 0.1 in SD units). In addition, we estimated the power (*P* < 5 × 10^−8^) of the Cochran’s *Q*-test for heterogeneity (implemented in the GWAMA software^14,15^) under these three different models. We performed simulations on 70,000 men and 70,000 women, a sample similar by size and sex ratio to our study (“Methods”), to evaluate the power of our analysis to detect sex dimorphism at established FG (*n* = 36) and FI (*n* = 19) loci after Bonferroni correction for multiple testing (*P*_heterogeneity_ < 0.05/36 or *P*_heterogeneity_ < 0.05/19)^6^.

**Fig. 4:**
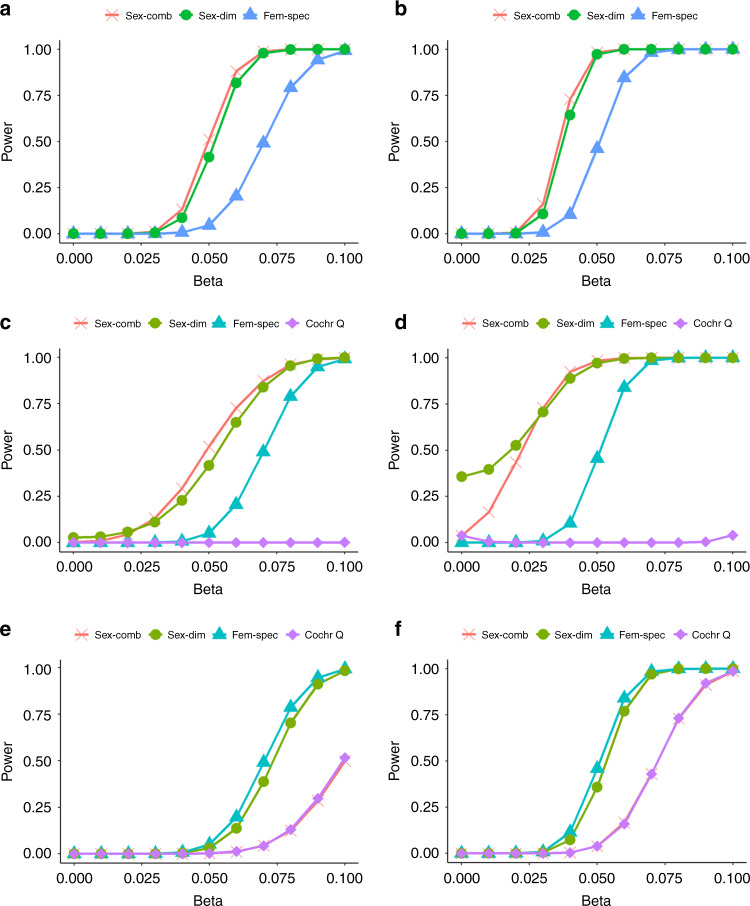
Power of tests for detecting sex heterogeneity through simulations. The power of sex-combined, sex-dimorphic and female-specific analyses, as well as Cochran’s *Q*-test was evaluated under three scenarios of sex-effects: no sex heterogeneity at **a** CAF = 0.05 and **b** CAF = 0.1, effects on both sexes with the presence of heterogeneity between them at **c** CAF = 0.05 and **d** CAF = 0.1, an effect specific to one sex only, e.g., women at **e** CAF = 0.05 and **f** CAF = 0.1. The power at *P* < 5 × 10^−8^ is given for all three tests: sex-combined, sex-dimorphic and female-specific. The power for the heterogeneity test implemented in GWAMA (Cochran’s *Q*-test) is also given. Simulations are based on 70,000 men and 70,000 women. For each parameter setting, 10,000 replicates of data were generated. CAF is the causal variant allele frequency and beta is the effect size in SD units in women. Within each scenario, we considered two CAFs (0.05 and 0.1) and a range of betas (from 0 to 0.1) representing the effect size in SD units in women. For the no sex heterogeneity setting, the beta in men is the same as in women; for the sex-dimorphic setting, the beta in men is fixed at 0.05 SD units; for the female-specific setting, the beta in men is fixed at zero.

For the scenario of homogenous allelic effects between men and women (i.e., no sex dimorphism), the sex-combined test was the most powerful to detect association with the causal variant across the whole range of allele frequencies (Fig. 4 and Supplementary Fig. 5). The 2-df sex-dimorphic analysis showed slightly less power due to the additional degree of freedom. The loss of power in the female-specific analysis occurred because of a reduction in sample size due to stratification by sex.

For the scenario of sex-dimorphic effects (effect size in men, *β*_males_, fixed at 0.05 SD units, and in women, *β*_females,_ variable), the most powerful test varied depending on the strength of the effect in women (Fig. 4, Supplementary Fig. 5). Overall, the 2-df sex-dimorphic test had the greatest power (>92%) across all effect sizes (from 0 to 0.1 in SD units) and for CAF ranging between 0.2 and 0.5, whereas the sex-combined analysis was more powerful when the effects on both sexes were similar (*β*_females_ = 0.04–0.06, *β*_males_ = 0.05) and for CAF ranging between 0.05 and 0.1. The female-specific approach was considerably less powerful than the sex-combined/-dimorphic analyses due to the smaller sample size. Under the same settings, the heterogeneity test was generally very underpowered (power < 34%) with our sample size, except for the situation of the variant being very common (CAF = 0.5) and in the presence of a large difference in effects between the two sexes (*β*_females_ = 0 or 0.10 and β_males_ = 0.05) (power > 81%).

We observed that the female-specific test was the most powerful analysis to detect a single-sex effect (effect only in women with the effect size in men fixed at zero) across all allele frequencies (Fig. 4, Supplementary Fig. 5). The slight loss of power of the 2-df sex-dimorphic test to identify such an effect was due to the additional degree of freedom to allow for heterogeneity in allelic effects between sexes. Furthermore, despite the increase in sample size, the sex-combined analysis was considerably less powerful compared to the other two approaches because of the diluted allelic effect by the inclusion of men. For the heterogeneity test, the power was good (>73%) only in the presence of a relatively strong effect in women (*β*_females_ range: 0.05–0.10), no effect in men, and for CAF range of 0.1–0.5.

Overall, based on simulations, our study had more than 78% power to detect heterogeneity at established loci in the presence of large differences in allelic effects between sexes or a relatively strong effect in a single sex and within the CAF range (i.e. *β* > 0.05 SD units difference for CAF = 0.1, *β* > 0.04 SD units for CAF = 0.2 and *β* > 0.03 SD units for CAF = 0.5) (Supplementary Fig. 5). For CAF = 0.05, this approach had more than 80% power to detect effects specific to one sex (*β*_females_ > 0.06 SD units and *β*_males_ = 0 SD units) but showed generally very low power (power < 45%) for effects larger in one sex than the other, a scenario that was most frequently observed for FG/FI loci.

## Discussion

These GWAS meta-analyses represent the largest effort, to date, to systematically evaluate sex dimorphism in genetic effects on fasting glycemic trait variability in up to 151,188 European ancestry individuals without diabetes. Using specifically developed methods and software tools^14,15^, we performed sex-dimorphic meta-analyses, equivalent to testing for phenotype association with SNPs allowing for heterogeneity in allelic effects between sexes. We demonstrated sex-dimorphic effects on FI at *IRS1* and *ZNF12* loci and evaluated the power of such analyses in a simulation study. We also detected seven novel FG loci with homogeneous effects between sexes. We identified FI sex-dimorphic genetic correlation genome-wide with WHRadjBMI and demonstrated a causal effect of WHRadjBMI on FI levels in women only.

In this large-scale study, we demonstrated a sex-dimorphic effect of *IRS1* on FI that was specific to men, in addition to those previously reported on body fat percentage, high-density lipoprotein cholesterol and triglycerides^10^. These locus-wise effects on other phenotypes were similar to the genome-wide genetic correlations between FI, two blood lipids and a number of obesity traits. For other loci, we have highlighted the cross-trait consistency compared to adiposity-related phenotypes. More specifically, the *COBLL1*/*GRB14* locus with female-specific effects on central obesity^8,11^ and on T2D^12^ showed nominally significant larger effects on FI in women.

The female-specific FI locus is at ubiquitously expressed *ZNF12*, encoding for zinc-finger protein 12, localized in the nucleoplasm of cells and involved in developmental control of gene expression. We provided support for *ZNF12* as a potential candidate in this locus through its expression in human beta cells and pancreatic islets, as well as higher RNA expression levels in women than in men in whole blood. Furthermore, *ZNF12* is a quantitative trait locus for glucose and insulin levels in rats (Rat Genome Database: IDs 1643535, 2303575, 1357337^43^). In humans, the lead SNP rs7798471 overlaps with the DNaseI hypersensitivity site from pancreatic adenocarcinoma (PA-TU-8988T, https://www.encodeproject.org/), which maps near the *ZNF12* alternative transcript start site. Interestingly, the *ZNF12*non-coding variant rs7798471 lies within a conserved DNA region. It is in high LD with a number of Neanderthal methylated variants, and is present in the archaic genome of a Denisova individual, suggesting that this genomic region might have introgressed into modern humans through admixture with Neanderthals and Denisovans^44^. This observation is similar to the T2D-associated variants at *SLC16A11/13* reported by SIGMA consortium^45^, being another example of admixture between archaic genome variants that influence physiology of complex traits today. We did not observe association between this variant and T2D in the sex-combined GWAS meta-analyses in European ancestry individuals^20^ indicating that the effects of this variant are on the reduced insulin sensitivity rather than T2D susceptibility.

Among the FG loci with sex-homogeneous effects, variants at the *MANBA/UBE2D3*, *NFATC3*, and *IGF1R* provided insights into pathways involved in glucose homeostasis and relationships with other complex phenotypes, including neurodegeneration, schizophrenia, and cancer^29,46^.

Genetically underpinned differences in glycemic trait variability by sex could reflect alterations in a variety of processes related to T2D pathophysiology. FG/FI genetic correlations with a range of metabolic traits, detected in our study for either sex, were in accordance with epidemiological observations^4^. For example, suggestively stronger inverse genetic correlation between FI and anorexia nervosa in women, compared to men was in line with observed higher insulin sensitivity in individuals with this disease^47^. Direct genetic correlations between FI and obesity traits are widely supported by epidemiological studies. The genetic correlation between FI and WHR is stronger in women than in men, and the causal relationship between WHR adjusted for BMI and insulin resistance is detected in women only. These observations suggest that central obesity in women is the driving risk factor for many pathologies where insulin resistance is among the symptoms, such as polycystic ovary syndrome and fatty liver disease.

Methods accounting for sex differences and interaction are more powerful in the presence of heterogeneity of allelic effects between men and women^14^. However, only recently, the development of fast-performance software tools for sex-dimorphic analysis enabled the current study^15^. Our simulation study highlighted that, given the current sample size, the 2-df sex-dimorphic test was more powerful, compared to the sex-combined approach, when causal variants had allelic effects specific to one sex and in the presence of heterogeneous allelic effects in men and women. When the allelic effects of the causal variant were similar between men and women, the sex-combined test was only slightly more powerful than the sex-dimorphic approach, especially for CAF ≤ 0.1. However, under the scenarios of effects that were larger in one sex than the other or specific to just one sex, the heterogeneity test was generally very underpowered. Nevertheless, our statistical power to detect sex differences in genetic effects within novel or established glycemic loci was still limited. In fact, at CAF = 0.2 and 0.02 SD units difference in effect estimates between men and women requires information from 125,000/125,000 men/women to achieve 80% power to detect sex-dimorphic effects at a nominal level of significance.

In conclusion, our study shows sex-dimorphic effects on FI at two genetic loci. Sex dimorphism in genetic effects on FI correlates genetically with such effects on WHRadjBMI, which is also causal for FI changes in women. This result is in line with previous epidemiological observations on insulin resistance as the process leading to pathophysiological differences between sexes^48^. Our findings position dissection of sex dimorphism in glycemic health as a steppingstone for understanding sex-heterogeneity in related traits and disease outcomes.

## Methods

### Participating studies

The following collection of studies were used: (1) 38 GWAS, including up to 80,512 individuals genotyped using either Illumina or Affymetrix genome-wide SNP arrays; (2) 27 studies with up to 47,150 individuals genotyped using the iSELECT Metabochip array (~197 K SNPs) designed to support efficient large-scale follow-up of putative associations for glycemic and other metabolic and cardiovascular traits; (3) 8 studies, including up to 21,173 individuals genotyped for custom variant sets; and (4) 4 studies, including up to 13,613 individuals from four family-based studies (sex-combined meta-analyses only, as detailed below). Detailed descriptions on the participating studies are provided in Supplementary Data 1. All participants were of European ancestry, without diabetes and mostly adults, although data from a total of 8,222 adolescents were also included in the meta-analyses (ALSPAC, French Young controls/obese, Leipzig-childhood and NFBC86 studies). All studies were approved by local ethics committees and all participants gave informed consent.

### Traits

Data were collected from participating studies with FG measured in mmol/L (*N*_maxmen_ = 67,506, *N*_maxwomen_ = 73,089) and FI measured in pmol/L (*N*_maxmen_ = 47,806, *N*_maxwomen_ = 50,404). Measures of FG made in whole blood were corrected to plasma level using the correction factor of 1.13^49^. FI was measured in serum. Similar to previous MAGIC efforts^22,50,51^, individuals were excluded from the analysis if they had a physician diagnosis of diabetes, were on diabetes treatment (oral or insulin), or had a fasting plasma glucose equal to or greater than 7 mmol/L. Individual studies applied further sample exclusions, including pregnancy, non-fasting individuals, and type 1 diabetes. Individuals from case-control studies were excluded if they had hospitalization or blood transfusion in the 2–3 months before phenotyping took place. Untransformed FG and natural logarithm transformed FI were analyzed at a study level. Detailed descriptions of study-specific glycemic measurements are given in Supplementary Data 1. Untransformed FG and natural logarithm transformed FI, HOMA-B, and HOMA-IR were analyzed at a study level.

### Genotyping and quality control

Commercial genome-wide arrays, the Metabochip^17^ or platforms with custom variant sets were used by individual studies for genotyping. Studies with genome-wide arrays undertook imputation of missing genotypes using the HapMap II CEU reference panel via MACH^52,53^, IMPUTE^54,55^, or BEAGLE^56^ software (Supplementary Data 1). For each study, samples reflecting duplicates, low call rate, gender mismatch, or population outliers were excluded. Low-quality SNPs were excluded by the following criteria: call rate <0.95, minor allele frequency (MAF) < 0.01, minor allele count < 10, Hardy–Weinberg *P* value < 10^−4^. After imputation, SNPs were also excluded for imputation quality score <0.5.

### Imputation to the 1000G reference panel

We imputed the summary statistics for FG and FI (combined and sex-stratified) to the 1000 Genomes reference panel^57^ using the summary statistics imputation method implemented in the SS-Imp v0.5.5 software^18,58^. We used the all-ancestries reference panel. SNPs with imputation quality score <0.7 were excluded after imputation.

### Statistical analysis

Each study performed single SNP association for men and women separately (sex-specific). The additive genetic effect of each SNP was estimated using a linear regression model adjusting for age (if applicable), study site (if applicable), and principal components. In case-control studies, the cases and controls were analyzed separately. Individual study results were corrected for residual inflation of the test statistics using genomic control (GC)^59^. The GC lambda values were estimated using test statistics from all SNPs for the GWAS. In Metabochip studies, GC values were estimated from test statistics from 5,041 SNPs selected for follow-up of QT-interval associations, as we perceived these to have the lowest likelihood of common architecture of associations with glycemic traits^59^.

SNP effect estimates and their standard errors were combined by a fixed effect model with inverse variance weighting using the GWAMA v2.2.3 software within the following three meta-analysis strategies: (1) sex-specific, where allelic effect estimates were combined separately within each sex (male-specific or female-specific), (2) sex-dimorphic, where male- and female-specific estimates were combined by allowing for heterogeneity in allelic effects between women and men (chi-squared distribution with two-degrees of freedom)^14^ and (3) sex-combined, where allelic effect estimates from men and women were combined. Studies with highly related individuals (Dundee, FamHS, FHS and Sardinia) were included only in the sex-combined meta-analysis (men and women were analyzed together at a study-level and an additional adjustment for sex was made). In addition, the heterogeneity of allelic effects between sexes was assessed using Cochran’s *Q*-test. Cochran’s statistic provides a test of heterogeneity of allelic effects at the *j*th SNP, and has an approximate chi-squared distribution with *N*_*j*-1_ degrees of freedom under the null hypothesis of consistency where *N*_*j*_ denotes the number of studies for which an allelic effect is reported. Both the sex-dimorphic meta-analysis framework and Cochran’s *Q* test for heterogeneity have been implemented in the GWAMA software^15^. The lambda values for FG and FI sex-differentiated and Cochran’s *Q* test were as follows: FG (*λ*_sex-differentiated_test_ = 1.06, *λ*_CochransQ_test_ = 1.01), FI (*λ*_sex-differentiated_test_ = 1.06, *λ*_CochransQ_test_ = 1.00).

### Sex-dimorphic effects at established and novel FG/FI loci

The heterogeneity in allelic effects between sexes was assessed at 36 FG and 19 FI established loci. A locus was considered to have heterogeneous effects between sexes if *P*_heterogeneity_ ≤ 0.0014 for FG and *P*_heterogeneity_ ≤ 0.0026 for FI after using Bonferroni correction for multiple testing within each set of trait independent loci. To identify a novel locus with sex-dimorphic effects (i.e. effect larger in one sex than the other or specific to just one sex), genome-wide significance in the sex-dimorphic meta-analysis (*P*_sex-dimorphic_ < 5 × 10^−8^, 2df) was required. Loci with homogeneous effects in women and men were identified by considering *P*_sex-combined_ < 5 × 10^−8^. SNPs were considered as novel if located more than 500 kb from, and not in LD (HapMap CEU/1000 Genomes EUR: *r*^2^ < 0.01) with any variant already known to be associated with the trait.

### Approximate conditional analysis

We performed approximate conditional analysis by using the Genome-Wide Complex Trait Analysis (GCTA) v1.24.4 tool to assess whether the signals within the *MANBA/UBE2D3* genomic region associated with FG represented independent associations or the same shared signal with multiple sclerosis and ulcerative colitis^33,34^. GCTA implements an approximate conditional analysis of phenotype associations using GWAS summary statistics while incorporating LD information from a reference sample. Here, we used individual level genotype data from the PIVUS study (European ancestry) as the LD reference. The GCTA approach allows the estimation of an adjusted effect size estimate with a corresponding *P* value for the association of a variant with a phenotype, corrected for the effect of another adjacent SNP or a group of SNPs, based on the extent of LD between them.

### Genetic correlation analysis

We assessed the genetic correlations between 201 traits publicly available in the LDHub^60^ and the sex-specific FG and FI using the bivariate LD score regression approach^61^. The bivariate LD score regression only requires GWAS summary statistics of two traits to evaluate their shared genetic components, and can account for confounding like sample overlap^61^. We considered the trait to have a statistically significant genetic correlation with FG/FI if the estimate attained *P* < 0.00012 (after Bonferroni correction for 201 traits and two sexes) in either women or men. Heterogeneity in the estimates between women and men was evaluated using Cochran’s *Q* statistic and *I*^2^ statistic which is independent of the number of studies. We considered evidence for heterogeneity at the nominal level of *P* < 0.05 for the Cochran’s *Q* test.

### Bidirectional two-sample MR analyses

We applied bidirectional MR to investigate the causality between WHRadjBMI and FI. MR provides estimates of the effect of modifiable exposures on disease unaffected by classical confounding or reverse causation, whenever randomized clinical trials are not feasible^62–64^. Genetic and phenotype data were available from the UK Biobank cohort (214,924 women and 183,739 men) for obtaining genetic instruments for WHRadjBMI from the general population. To look at the reverse, i.e., the potential causal effect of FI on WHRadjBMI, we used genetic instruments for FI and genome-wide summary results from the present study (50,404 women and 47,806 men). We used independent (*r*^2^ < 0.001) SNPs that reached genome-wide significance (*P* ≤ 5 × 10^−8^) in the combined (women and men) WHR GWAS as instruments for WHR. We obtained 222 WHRadjBMI SNPs for women and 222 WHRadjBMI SNPs for men. SNP-WHRadjBMI associations were expressed in terms of Z-scores. For FI, we used as instruments the 19 SNPs established for FI by MAGIC (Supplementary Data 3).

The random-effect inverse-variance weighted (IVW) method was used to obtain the combined MR estimate from the causal estimates of each individual variant in the instrument derived by the ratio method^65^. Standard errors were calculated using the Delta method^66^. We employed MR-Egger regression to obtain causal estimates that are more robust to the inclusion of invalid instruments^67^. We tested for the presence of a causal effect of (1) WHRadjBMI on FI in women, (2) FI on WHRadjBMI in women, (3) WHRadjBMI on FI in men, and (4) FI on WHRadjBMI in men. Heterogeneity in the IVW estimates from each individual variant was tested using Cochran’s *Q* test. The presence of directional pleiotropy was tested with the MR-Egger intercept test where a significant non-zero intercept term can be indicative of directional pleiotropy. We have additionally performed analyses of four causal relationships: WC adjBMI on FI in women and men and HCadjBMI on FI in women and men to assess, which fat depot drives the causal relationship between central adiposity and FI. All MR analyses were performed using the R package TwoSampleMR v0.5.4.

### Simulations to assess the power of tests to detect sex-heterogeneity under different scenarios

A range of scenarios of effects on the two sexes were considered and the power of three types of analysis (sex-combined, 2df sex-dimorphic and female-specific) to pick any associations with evidence for sex-heterogeneity was assessed. More specifically, three models were tested: (1) no heterogeneity between the two sexes, (2) effects on both sexes with the presence of heterogeneity between them and (3) an effect specific to one sex only, e.g., women. Within each scenario, a range of causal variant effect allele frequencies (ranging from 0.05 to 0.5) and effect size estimates (ranging from 0 to 0.1) in SD units in women were assessed. In addition, the power of the Cochran’s *Q* test for heterogeneity (implemented in GWAMA) was evaluated under these three different models.

Furthermore, the power of our study to detect sex heterogeneity at established FG (*n* = 36) and FI (*n* = 19) loci was assessed by simulations using the approach that ignores *P*_sex-dimorphic_ and considers only a *P*_heterogeneity_ < 0.05 or *P*_heterogeneity_ adjusted for multiple testing (*P*_heterogeneity_ < 0.05/36 or *P*_heterogeneity_ < 0.05/19).

### Tissue expression of genes within the ZNF12 locus

Expression profiles from fat, LCL, and skin tissues from women for genes within the *ZNF12* region have demonstrated the expression of three genes (*ZNF12*, *KDELR2* and *DAGLB*) in our analyses. Therefore, three genes at this locus were followed-up using quantitative RT-PCR. Commercial cDNAs from the Human MTC panel I (BD Biosciences Clontech) were diluted fivefold. For each sample, 4 µl was used in a 20 µl quantitative RT-PCR reaction including 10 µl of TaqMan gene expression master mix (Applied Biosystems®) and 1 µl of the TaqMan gene expression assay (Applied Biosystems) (TaqMan probes: KDELR2-Hs01061971_m1, ZNF12-Hs00212385_m1, RGS17-Hs00202720_m1, DAGLB-Hs00373700_m1). Islets of Langerhans and flow sorted beta cells were obtained from adult brain-dead donors in accordance with the French regulation and with the local institutional ethical committee^68^. Total RNA was extracted using Nucleospin RNA II kit (Macherey Nagel). For each sample, 1 µg of total RNA was transcribed into cDNA using the cDNA Archive Kit (Applied Biosystems^®^) or random primed first strand synthesis (Applied Biosystems^®^). Resulting cDNAs were diluted ten-fold and 4 µl of each sample were used in a 20 µl quantitative RT-PCR reaction including 10 µl of TaqMan gene expression master mix (Applied Biosystems^®^) and 1 µl of TaqMan gene expression assay (Applied Biosystems). Quantitative RT-PCR analyses were performed using the ABI 7900 HT SDS 2.4, RQ manager v1.2.1, and DataAssist v3.0 software and each sample was run in triplicate. Expression of genes was reported as a relationship to the respective tissue expression of three housekeeping genes (*PPIA*, *B2M* and *HPRT*).

### RNA expression in blood

Look-ups for novel and known genes with evidence of sex heterogeneity were done in the whole blood RNA expression data from NTR and NESDA. For the NTR participants, venous (7–11 a.m) blood samples were drawn after overnight fasting. Within 20 min of sampling, heparinized whole blood was transferred into PAXgene Blood RNA tubes (Qiagen) and stored at −20 °C. The PAXgene tubes were shipped to the Rutgers University Cell and DNA Repository (RUCDR), USA, where RNA was extracted using Qiagen Universal liquid handling system (PAXgene extraction kits as per the manufacturer’s protocol). For the NESDA subjects, venous overnight fasting (8–10 a.m.) blood samples were obtained in one 7-ml heparin-coated tube (Greiner Bio-One, Monroe, NC). Between 10 and 60 min after blood draw, 2.5 ml of blood was transferred into a PAX-gene tube (Qiagen, Valencia, CA). This tube was left at room temperature for a minimum of 2 h and then stored at −20 °C. Total RNA was extracted at the VU University Medical Center (Amsterdam) according to the manufacturer’s protocol (Qiagen).

Gene expression assays were conducted at the Rutgers University Cell and DNA Repository (RUCDR, http://www.rucdr.org) for all samples. RNA quality and quantity were assessed by Caliper AMS90 with HT DNA5K/RNA LabChips. RNA samples with abnormal ribosomal subunits in the electropherograms were removed. NTR and NESDA samples were randomly assigned to plates. For cDNA synthesis, 50 ng of RNA was reverse-transcribed and amplified in a plate format on a Biomek FX liquid handling robot (Beckman Coulter) using Ovation Pico WTA reagents per the manufacturer’s protocol (NuGEN). Products purified from single primer isothermal amplification were then fragmented and labeled with biotin using Encore Biotin Module (NuGEN). Prior to hybridization, the labeled cDNA was analyzed using electrophoresis to verify the appropriate size distribution (Caliper AMS90 with a HT DNA 5 K/RNA LabChip). Samples were hybridized to Affymetrix U219 array plates (GeneTitan). The U219 array contains 530,467 probes for 49,293 transcripts. All probes are 25 bases in length and designed to be “perfect match” complements to a designated transcript. Array hybridization, washing, staining, and scanning were carried out in an Affymetrix GeneTitan System per the manufacturer’s protocol.

Gene expression data were required to pass standard Affymetrix quality control metrics (Affymetrix expression console) before further analysis. Probes were removed when their location was uncertain or intersected a polymorphic SNP. Expression values were obtained using RMA normalization implemented in Affymetrix Power Tools v 1.12.0. Finally, samples with insufficient RNA quality (*D* < 5) or sex mismatch were removed.

Statistical analysis was done with linear mixed modeling for the genes of interest (Supplementary Tables 10 and 11b) where the average gene expression by all probes in the gene was predicted by sex, as well as the following covariates: age, smoking status, RNA quality, hemoglobin, study, time of blood sampling, month of blood sampling, time between blood sampling and RNA extraction, and the time between RNA extraction and RNA amplification. Overall, sex-dimorphic effects in this analysis represented the significance of the effect of sex in the linear regression analysis, where, after accounting for relevant covariates, the average gene expression was predicted by sex. Covariates not included in the model due to lack of significance of their effects were alcohol use, education level, time between RNA amplification and RNA fragmentation, time between RNA fragmentation and RNA hybridization, depression status, psychotropic medication, and white blood cell counts. The random effects included in the model were plate, well, family ID, and zygosity (one factor for each monozygotic twin pair). The total number of samples in the analyses was 3,621 individuals.

### Gene expression in human pancreatic islets and eQTL analyses

The islets from 89 cadaver donors of European ancestry were prepared for gene expression analysis. All procedures were approved by the ethics committee at Lund University. Purity of islets was assessed by dithizone staining, while measurement of DNA content and estimate of the contribution of exocrine and endocrine tissue were assessed by measuring expression of pancreatic lipase, alpha 2 amylase and chymotrypsin 2 (as markers of exocrine) and somatostatin and glucagon (as markers of endocrine tissue)^69^. The islets were cultured in CMRL 1066 (ICN Biomedicals) supplemented with 10 mM HEPES, 2 mM l-glutamine, 50 µg/ml gentamicin, 0.25 µg/ml Fungizone (GIBCO), 20 µg/ml ciprofloxacin (Bayer Healthcare), and 10 mM nicotinamide at 37 °C (5% CO2) for 1–9 days prior to RNA preparation. Total RNA was isolated with the AllPrep DNA/RNA Mini Kit following the manufacturer’s instructions (Qiagen). RNA quality and concentration were measured using an Agilent 2100 bioanalyzer (Bio-Rad) and a Nanodrop ND-1000 (NanoDrop Technologies).

### RNA sequencing and analysis of gene expression

Islet preparation for RNA sequencing was made using Illumina’s TruSeq RNA Sample Preparation Kit according to their recommendations using 1 µg of high quality total RNA. The target insert size was 300 bp and it was sequenced using a paired end 101 bp protocol on the HiSeq2000 platform (Illumina). Quality assessment was made pre- and post-sample preparation on the 2100 Bioanalyzer (Agilent). Illumina Casava v.1.8.2 software was used for base calling. Paired-end 101 bp length output reads were aligned to the human reference genome (hg19) with TopHat v.2.0.2^70^ using Bowtie v.0.12.8^71^. The TopHat parameters explicitly used are tophat -p 30 -G genes.gtf --library-type fr-unstranded -r 100 -F 0.05 --microexon-search. Gene expression was measured as the normalized sum of expression of all exons. Exons were defined as non-overlapping unique exonic units^72^. The dexseq_count python script (“Data availability”) was used by counting uniquely mapped reads in each exon. Gene and exon expression normalizations were then performed using the TMM method^73^, and further normalization was applied by adjusting the expression to gene or exon length, respectively. In addition, only the genes and exons that had reads mapped to them in at least 5% of the samples were kept. The Cufflinks tool v.1.3.0^74^ was used to detect novel genetic loci. Intergenic gene loci were kept if they did not overlap any GENCODE v.12 gene^75^, UCSC and Ensembl gene structures, had exon–exon junction reads mapped to them, had at least two exons with no Ns, and were expressed (non-null read coverage) in at least 5% of the samples. Coding potential of these novel intergenic loci was assessed with the Coding Potential Assessment Tool v1.2.2^76^.

### Gene expression in islet donors

Samples were stratified based upon glucose tolerance estimated from HbA1c, i.e., donors with normal glucose tolerance (HbA1c < 6%, *n* = 51), IGT (6% ≤ HbA1c < 6.5%, *n* = 15), and T2D (HbA1c ≥ 6.5%, *n* = 12). A linear model adjusting for age and sex as implemented in the R Matrix eQTL package^77^ was used to determine the expression of genes associated with T2D status.

Genotyping was performed on the Illumina HumanOmniExpress 12v1 C chips and genotype calling was done with the Illumina Genome studio v2.0 software. All the samples passed standard genotype quality control metrics: sample call rate >98%, only European ancestry assessed by principal component analysis comparisons with HapMap populations, gender matched, no relatedness, and no genome-wide heterozygosity outliers. SNPs were removed if SNP call rate <98% and Hardy–Weinberg equilibrium test *P* values <5.7 × 10^−7^. Individual genotypes were imputed to 1000 Genomes data, using IMPUTE2 and the March 2012 release of the 1000 Genomes Phase I panel. The program SHAPEIT v2^78^ was used for the pre-phasing. Probabilistic genotypes were used for the subsequent analyses and after imputation, SNPs were filtered using a MAF > 5% and an IMPUTE2 info value of >0.8.

eQTL analyses were carried out on samples from 89 individuals. Associations were computed between gene expression levels (eQTL) and top SNPs within 250 kb up- or downstream of each of these genes. We used a linear model adjusting for age and sex as implemented in the R Matrix eQTL v2 package^77^. Results are show with *P* values with age and sex as covariates, after the false discovery rate (FDR) and obtained after doing 10,000 permutations.

### Sex-specific eQTL

Sex-specific eQTL analyses were performed in the MolOBB, MuTHER, Karolinska Institutet, HapMap 2, and NTR/NESDA. Each study performed two types of analysis as described below, unless otherwise stated under the study description. Two models were used.

1. Model with the same slope in each gender:
  1 $$y_i = \mu _{s(i)} + \pi _{p(i)} + \beta \, \times \,g_{(i)} + \varepsilon _i,$$
  where *i* indexes subject, *s(i)* ∈ [Male, Female] maps subject to gender, *g(i)* ∈ [0,1,2] maps subject to a genotype and *p(i)* maps subject to a plate.
2. Model with different association in each gender:

2 $$y_i = \mu _{s(i)} + \pi _{p(i)} + \beta _{s(i)}\, \times \,g_{(i)} + \varepsilon _i.$$

To investigate whether genes are differentially expressed between males and females, each study fitted a linear mixed model using the R package Maanova. Gender and plate were fitted as fixed effects. The *P* values from the Fs test were corrected for multiple testing using the FDR (Benjamini Hochberg) across the tested genes, and probe sets were considered significant if the adjusted P value of the Fs test was <0.01.

### MolOBB cohort data collection and pre-processing

From 73 individuals (recruited on the basis of case/control status for Metabolic Syndrome), a gluteal fat sample and an abdominal fat sample were extracted at the Oxford Centre for Diabetes, Endocrinology and Metabolism as part of the MolOBB study. A total of 143 samples were obtained, with 71 subjects successfully donating both tissue types, and one individual donating only gluteal fat. Subcutaneous adipose tissue from the abdominal wall was taken at the level of the umbilicus; gluteal tissue was taken from the upper outer quadrant of the buttock. Total RNA was extracted with TRIreagent (SIGMA-ALDRICH) from the fat biopsies. For six of the subjects, twice the amount of RNA was extracted from each sample, and the RNA was split into two aliquots before labeling (i.e. each of six gluteal, and six abdominal, samples was run in technical replicate). Labeled RNA was hybridized onto Affymetrix Human Genome U133 Plus 2.0 gene-expression microarrays (hgu133plus2 arrays), washed, stained, and scanned for fluorescence intensity indicative of gene expression level. One sample was hybridized to each array. Quality control checks were performed on the basis of signal intensities, background intensity, expression of control genes, and spike-ins, as well as spatial representation of the intensities on each array. After outlying arrays had been removed, there remained data from 54 abdominal fat samples (4 in technical duplicate), and 65 gluteal fat samples (5 in technical duplicate); 49 subjects had both gluteal and abdominal samples remaining in the analysis.

The majority of the probes on the hgu133plus2 arrays were collected into 17,726 non-overlapping probe sets according to ENTREZG annotations^79^. For each datasets all arrays were preprocessed separately using GC robust multi-array procedure. Gene-specific expression summaries were averaged across technical replicates of a sample. We then filtered the data, retaining only those probe sets that were annotated to an autosomal location, and also showed a mean intensity above 4 arbitrary units of log2(intensity) in at least 10% of individuals. After this filtering stage, there remained 8,941 probe sets.

### MuTHER data collection and pre-processing

The MuTHER (Multiple Tissue Human Expression Resource) collection^41^ includes LCLs, skin and adipose tissue derived simultaneously from a set of well-phenotyped healthy female twins. Whole-genome expression profiling of the samples, each with either two or three technical replicates, were performed using the Illumina Human HT-12 V3 BeadChips (Illumina Inc) according to the protocol supplied by the manufacturer. Log2-transformed expression signals were normalized separately per tissue as follows: quantile normalization was performed across technical replicates of each individual followed by quantile normalization across all individuals.

Genotyping was done with a combination of Illumina arrays (HumanHap300, HumanHap610Q, 1 M‐Duo and 1.2MDuo 1 M). Untyped HapMap2 SNPs were imputed using the IMPUTE v2.0 software. The number of samples with genotypes and expression values per tissue was 778 LCL, 667 skin, and 776 adipose, respectively. Association between all SNPs (MAF > 5%, IMPUTE info >0.8) within a gene or within 1MB of the gene transcription start or end site and normalized expression values were performed with the GenABEL/ProbABEL packages using the polygenic linear model incorporating a kinship matrix in GenABEL followed by the ProbABEL mmscore score test with imputed genotypes. Age and experimental batch were included as cofactors in the adipose and LCL analysis, while age, experimental batch and concentration were included as cofactors in the skin analysis.

### Karolinska Institutet data collection and pre-processing

The genotypes of the ASAP dataset were measured on Illumina 610wQuad arrays and the expression was measured on Affymetrix ST 1.0 exon arrays. In this analysis, five tissue types have been included in a total of 699 samples: 89 mammary artery intima-media (ASAP_MMed), 212 liver (ASAP_L), 138 aorta intima-media (ASAP_AMed), 133 aorta adventitia (ASAP_AAdv), 127 heart (ASAP_H). All SNP positions are from dbSNP 132 through biomaRt. All gene positions are from ensembl GRCh37.p3 through biomaRt. All genes within 500 kb were included.

### Karolinska Institutet data statistical analysis

For the analysis of eQTLs and gender the given formula

3 $$y_i = \mu _{s(i)} + \pi _{p(i)} + \beta \, \times \,g_{(i)} + \varepsilon _i$$

was modified as follows: The p(i)-component was omitted as each sample is run on an individual plate (on batch effects and normalizations issues of expression arrays can be found in^80^). A total of 3,203 association tests were performed during these calculations: 5 tissues, 33 SNPs, 181 genes within 500 kb, and 3 different tests for each (all, male-only and female-only). Multiple correcting thresholds were therefore calculated, both using the Bonferroni method (*P* < 1.5 × 10^−5^) and the two-stage Benjamini–Hochberg FDR-5% method (*P* < 1.8 × 10^−5^) as implemented in the R-package multtest.

Student’s *T* test was used for the differential expression analysis. As there are no plate effects to take into account, this will largely provide the same results. Of the 135 genes selected for the look-up, the following 23 were not found on the microarray: *AK055550, ATP5EP2, BTF3P7, C7orf28B, CENTD2, CR593175, DQ485453, FBXL10, GRID21P, GRP85, LOC389436, LOC441376, MIR139, MIR32, MIR583, MIR597, NCRNA00261, PMS2CL, RPS3P4, RSPH10B2, ZNF853, ZNHHC4, Zep-1*. In most cases, this was because the HGCN gene symbol for that gene did not exist.

The *P* values and fold-change of expression changes between male and female samples were calculated for the remaining 112 genes. A total of 560 association tests were performed during these calculations: 5 tissues and 112 probe sets. Multiple correcting thresholds were therefore calculated, both using the Bonferroni method (*P* < 4.96e−05) and the two-stage Benjamini–Hochberg FDR-5% method (*P* < 0.0015) as implemented in the R-package multtest. A FDR-1% corresponds to *P* < 9.0 × 10^−5^.

### Functional and regulatory elements enrichment analysis

We used the GARFIELD v2 tool^81,82^ on the sex-specific meta-analysis results to assess enrichment of the FG/FI associated variants within functional and regulatory features. GARFIELD integrates data for genic annotations, chromatin states, DNaseI hypersensitive sites, transcription factor binding sites, FAIRE-seq elements, and histone modifications, among others, from a number of publicly available cell lines. We considered enrichment to be statistically significant if the FG/FI sex-specific GWAS *P* value reached *P* = 1 × 10^−8^ and the enrichment analysis *P* value was <6.2 × 10^−6^ (Bonferroni corrected for 2,040 annotations and two sexes).

### Reporting summary

Further information on research design is available in the Nature Research Reporting Summary linked to this article.

## Supplementary information

Supplementary Information

Peer Review File

Reporting Summary

Description of Additional Supplementary Files

Supplementary Data 1

Supplementary Data 2

Supplementary Data 3

Supplementary Data 4

Supplementary Data 5

Supplementary Data 6

Supplementary Data 7

Supplementary Data 8

Supplementary Data 9

Supplementary Data 10

Supplementary Data 11

## Data Availability

GWAS summary statistics for FG/FI analyses presented in this manuscript are deposited on https://www.magicinvestigators.org/downloads/ and will be also be available through the NHGRI-EBI GWAS Catalog https://www.ebi.ac.uk/gwas/downloads/summary-statistics. Raw files for RNA-seq mRNA expression in islet donors have been deposited in NCBI GEO database with the accession code GSE50398. Summary-level GWAS results for genetic correlation analysis with glycemic traits were downloaded from the LDHub database (http://ldsc.broadinstitute.org/ldhub/). Islets from 89 cadaver donors were provided by the Nordic Islet Transplantation Programme (http://www.medscinet.com/nordicislets/). The dexseq_count python script for RNA sequencing analysis in human pancreatic islets was downloaded from http://www-huber.embl.de/pub/DEXSeq/analysis/scripts/. Raw files for RNA-seq mRNA expression in islet donors have been deposited in NCBI GEO database with the accession code GSE50398.
