# Publisher Correction: Sex-dimorphic genetic effects and novel loci for fasting glucose and insulin variability

**DOI:** 10.1038/s41467-021-21276-3

**Published:** 2021-02-08

**Authors:** Vasiliki Lagou, Reedik Mägi, Jouke- Jan Hottenga, Harald Grallert, John R. B. Perry, Nabila Bouatia-Naji, Letizia Marullo, Denis Rybin, Rick Jansen, Josine L. Min, Antigone S. Dimas, Anna Ulrich, Liudmila Zudina, Jesper R. Gådin, Longda Jiang, Alessia Faggian, Amélie Bonnefond, Joao Fadista, Maria G. Stathopoulou, Aaron Isaacs, Sara M. Willems, Pau Navarro, Toshiko Tanaka, Anne U. Jackson, May E. Montasser, Jeff R. O’Connell, Lawrence F. Bielak, Rebecca J. Webster, Richa Saxena, Jeanette M. Stafford, Beate St Pourcain, Nicholas J. Timpson, Perttu Salo, So-Youn Shin, Najaf Amin, Albert V. Smith, Guo Li, Niek Verweij, Anuj Goel, Ian Ford, Paul C. D. Johnson, Toby Johnson, Karen Kapur, Gudmar Thorleifsson, Rona J. Strawbridge, Laura J. Rasmussen-Torvik, Tõnu Esko, Evelin Mihailov, Tove Fall, Ross M. Fraser, Anubha Mahajan, Stavroula Kanoni, Vilmantas Giedraitis, Marcus E. Kleber, Günther Silbernagel, Julia Meyer, Martina Müller-Nurasyid, Andrea Ganna, Antti-Pekka Sarin, Loic Yengo, Dmitry Shungin, Jian’an Luan, Momoko Horikoshi, Ping An, Serena Sanna, Yvonne Boettcher, N. William Rayner, Ilja M. Nolte, Tatijana Zemunik, Erik van Iperen, Peter Kovacs, Nicholas D. Hastie, Sarah H. Wild, Stela McLachlan, Susan Campbell, Ozren Polasek, Olga Carlson, Josephine Egan, Wieland Kiess, Gonneke Willemsen, Johanna Kuusisto, Markku Laakso, Maria Dimitriou, Andrew A. Hicks, Rainer Rauramaa, Stefania Bandinelli, Barbara Thorand, Yongmei Liu, Iva Miljkovic, Lars Lind, Alex Doney, Markus Perola, Aroon Hingorani, Mika Kivimaki, Meena Kumari, Amanda J. Bennett, Christopher J. Groves, Christian Herder, Heikki A. Koistinen, Leena Kinnunen, Ulf de Faire, Stephan J. L. Bakker, Matti Uusitupa, Colin N. A. Palmer, J. Wouter Jukema, Naveed Sattar, Anneli Pouta, Harold Snieder, Eric Boerwinkle, James S. Pankow, Patrik K. Magnusson, Ulrika Krus, Chiara Scapoli, Eco J. C. N. de Geus, Matthias Blüher, Bruce H. R. Wolffenbuttel, Michael A. Province, Goncalo R. Abecasis, James B. Meigs, G. Kees Hovingh, Jaana Lindström, James F. Wilson, Alan F. Wright, George V. Dedoussis, Stefan R. Bornstein, Peter E. H. Schwarz, Anke Tönjes, Bernhard R. Winkelmann, Bernhard O. Boehm, Winfried März, Andres Metspalu, Jackie F. Price, Panos Deloukas, Antje Körner, Timo A. Lakka, Sirkka M. Keinanen-Kiukaanniemi, Timo E. Saaristo, Richard N. Bergman, Jaakko Tuomilehto, Nicholas J. Wareham, Claudia Langenberg, Satu Männistö, Paul W. Franks, Caroline Hayward, Veronique Vitart, Jaakko Kaprio, Sophie Visvikis-Siest, Beverley Balkau, David Altshuler, Igor Rudan, Michael Stumvoll, Harry Campbell, Cornelia M. van Duijn, Christian Gieger, Thomas Illig, Luigi Ferrucci, Nancy L. Pedersen, Peter P. Pramstaller, Michael Boehnke, Timothy M. Frayling, Alan R. Shuldiner, Patricia A. Peyser, Sharon L. R. Kardia, Lyle J. Palmer, Brenda W. Penninx, Pierre Meneton, Tamara B. Harris, Gerjan Navis, Pim van der Harst, George Davey Smith, Nita G. Forouhi, Ruth J. F. Loos, Veikko Salomaa, Nicole Soranzo, Dorret I. Boomsma, Leif Groop, Tiinamaija Tuomi, Albert Hofman, Patricia B. Munroe, Vilmundur Gudnason, David S. Siscovick, Hugh Watkins, Cecile Lecoeur, Peter Vollenweider, Anders Franco-Cereceda, Per Eriksson, Marjo-Riitta Jarvelin, Kari Stefansson, Anders Hamsten, George Nicholson, Fredrik Karpe, Emmanouil T. Dermitzakis, Cecilia M. Lindgren, Mark I. McCarthy, Philippe Froguel, Marika A. Kaakinen, Valeriya Lyssenko, Richard M. Watanabe, Erik Ingelsson, Jose C. Florez, Josée Dupuis, Inês Barroso, Andrew P. Morris, Inga Prokopenko, Meta-Analyses of Glucose and Insulin-related traits Consortium (MAGIC)

**Affiliations:** 1 Wellcome Centre for Human Genetics, University of Oxford, Oxford, UK; 2 Department of Microbiology and Immunology, Laboratory of Adaptive Immunity, KU Leuven Leuven, Belgium; 3 VIB-KU Leuven Center for Brain and Disease Research, Leuven, Belgium; 4 Estonian Genome Centre, Institute of Genomics, University of Tartu, Tartu, Estonia; 5 Department of Biological Psychology, Vrije Universiteit, Amsterdam, The Netherlands; 6 Amsterdam Public Health Research Institute, VU University medical center, Amsterdam, The Netherlands; 7 Research Unit of Molecular Epidemiology, Institute of Epidemiology, Helmholtz Zentrum München Research Center for Environmental Health, Neuherberg, Germany; 8 German Center for Diabetes Research (DZD, München-Neuherberg, Germany; 9 MRC Epidemiology Unit, University of Cambridge School of Clinical Medicine, Cambridge, UK; 10 University of Lille Nord de France, Lille, France; 11 CNRS UMR8199, Institut Pasteur de Lille, Lille, France; 12 INSERM U970, Paris Cardiovascular Research Center PARCC, 75006 Paris, France; 13 Department of Life Sciences and Biotechnology, University of Ferrara, Ferrara, Italy; 14 Boston University Data Coordinating Center, Boston, MA USA; 15 Department of Psychiatry, VU University Medical Center, Amsterdam, The Netherlands; 16 MRC Integrative Epidemiology Unit, University of Bristol, Bristol, UK; 17 Bristol Medical School, University of Bristol, Bristol, UK; 18 Institute for Bioinnovation, Biomedical Sciences Research Center Al. Fleming, Vari, Greece; 19 Department of Medicine, Imperial College London, London, UK; 20 Cardiovascular Medicine Unit, Center for Molecular Medicine, Department of Medicine, Karolinska Institutet, Stockholm, Karolinska University Hospital, Solna, Sweden; 21 Institute for Molecular Bioscience, The University of Queensland, Brisbane, QLD 4072 Australia; 22 Department of Epidemiology Research, Statens Serum Institut, Copenhagen, Denmark; 23 Université de Lorraine, IGE-PCV, F-54000 Nancy, France; 24 Genetic Epidemiology Unit, Department of Epidemiology, Erasmus Medical Center, Rotterdam, the Netherlands; 25 CARIM School for Cardiovascular Diseases and Maastricht Centre for Systems Biology (MaCSBio, Maastricht University, Maastricht, The Netherlands; 26 Department of Physiology, Maastricht University, Maastricht, The Netherlands; 27 MRC Human Genetics Unit, MRC Institute of Genetics and Molecular Medicine, University of Edinburgh, Western General Hospital, Edinburgh, UK; 28 Translational Gerontology Branch, Longitudinal Study Section, National Institute on Aging, Baltimore, MD USA; 29 Department of Biostatistics and Center for Statistical Genetics, University of Michigan, Ann Arbor, MI USA; 30 Division of Endocrinology, Diabetes, and Nutrition, Department of Medicine, University of Maryland, School of Medicine, Baltimore, MD USA; 31 Department of Epidemiology, School of Public Health, University of Michigan, Ann Arbor, MI USA; 32 Laboratory for Cancer Medicine, Harry Perkins Institute of Medical Research, University of Western Australia Centre for Medical Research, Nedlands, WA Australia; 33 Broad Institute of Harvard and Massachusetts Institute of Technology (MIT), Cambridge, MA USA; 34 Center for Human Genetic Research, Massachusetts General Hospital, Boston, MA USA; 35 Department of Genetics, Harvard Medical School, Boston, MA USA; 36 Departmentartment of Anesthesia, Critical Care and Pain Medicine, MGH, Boston, MA USA; 37 Department of Biostatistics and Data Science, Division of Public Health Sciences, Wake Forest University School of Medicine, Winston-Salem, NC USA; 38 Public Health Genomics Unit, Department of Chronic Disease Prevention, Finnish Institute for Health and Welfare, Helsinki, Finland; 39 Wellcome Trust Sanger Institute, Wellcome Trust Genome Campus, Hinxton, UK; 40 Department of Epidemiology Erasmus MC, Rotterdam, the Netherlands; 41 Icelandic Heart Association, Kopavogur, Iceland; 42 Faculty of Medicine, University of Iceland, Reykjavik, Iceland; 43 Cardiovascular Health Research Unit, University of Washington, Seattle, WA USA; 44 Department of Medicine, University of Washington, Seattle, WA USA; 45 Department of Cardiology, University of Groningen, University Medical Center Groningen, Groningen, The Netherlands; 46 Cardiovascular Medicine, Radcliffe Department of Medicine, University of Oxford, Oxford, UK; 47 Robertson Centre for Biostatistics, University of Glasgow, Glasgow, UK; 48 Institute of Biodiversity, Animal Health & Comparative Medicine, University of Glasgow, Glasgow, UK; 49 William Harvey Research Institute, Barts and The London School of Medicine and Dentistry, Queen Mary University of London, London, UK; 50 NIHR Barts Cardiovascular Biomedical Research Unit, Barts and The London School of Medicine and Dentistry, Queen Mary University of London, London, UK; 51 Department of Medical Genetics, University of Lausanne, Lausanne, Switzerland; 52 deCODE Genetics, Reykjavik, Iceland; 53 Cardiovascular Medicine Unit, Department of Medicine, Solna, Karolinska Institutet, Stockholm, Sweden; 54 Center for Molecular Medicine, Karolinska University Hospital Solna, Stockholm, Sweden; 55 Institute of Health and Wellbeing, University of Glasgow, Glasgow, UK; 56 Department of Preventive Medicine, Northwestern University Feinberg School of Medicine, Chicago, IL USA; 57 Department of Medical Sciences, Molecular Epidemiology and Science for Life Laboratory, Uppsala University, Uppsala, Sweden; 58 Usher Institute, University of Edinburgh, Edinburgh, UK; 59 Synpromics Ltd, Roslin Innovation Centre, Easter Bush Campus, Edinburgh, EH25 9RG UK; 60 Wellcome Trust Sanger Institute, Hinxton, UK; 61 Department of Public Health and Caring Sciences, Uppsala Universitet, Uppsala, Sweden; 62 Vth Department of Medicine, Medical Faculty Mannheim, Heidelberg University, Mannheim, Germany; 63 Division of Angiology, Department of Internal Medicine, Medical University of Graz, Graz, Austria; 64 Institute of Genetic Epidemiology,Helmholtz Zentrum München, German Research Center for Environmental Health, Neuherberg, Germany; 65 Institute of Medical Informatics, Biometry and Epidemiology, Chair of Epidemiology and Chair of Genetic Epidemiology, Ludwig-Maximilians-Universität, Munich, Germany; 66 Department of Medicine I, University Hospital Grosshadern, Ludwig-Maximilians-University, Munich, Germany; 67 Institute of Medical Biostatistics, Epidemiology and Informatics (IMBEI, University Medical Center, Johannes Gutenberg University, 55101 Mainz, Germany; 68 Analytic and Translational Genetics Unit, Massachusetts General Hospital, Boston, MA USA; 69 Program in Medical and Population Genetics, Broad Institute of MIT and Harvard, Cambridge, MA USA; 70 Stanley Center for Psychiatric Research, Broad Institute of MIT and Harvard, Cambridge, MA USA; 71 Institute for Molecular Medicine Finland, FIMM, University of Helsinki, Helsinki, Finland; 72 Public Health Genomics Unit, Finnish Institute for Health and Welfare, Helsinki, Finland; 73 Department of Public Health & Clinical Medicine, Umeå University, Umeå, Sweden; 74 Department of Clinical Sciences, Genetic and Molecular Epidemiology Unit, Skåne University Hospital Malmö, Malmö, Sweden; 75 Department of Odontology, Umeå University, Umeå, Sweden; 76 Oxford Centre for Diabetes, Endocrinology and Metabolism, University of Oxford, Oxford, UK; 77 RIKEN, Center for Integrative Medical Sciences, Laboratory for Endocrinology, Metabolism and Kidney Disease, Yokohama, Japan; 78 Division of Statistical Genomics, Washington University School of Medicine, St. Louis, MO USA; 79 Istituto di Ricerca Genetica e Biomedica, CNR, Monserrato, Italy; 80 Department of Genetics, University Medical Center Groningen, University of Groningen, Groningen, the Netherlands; 81 Department of Medicine, University of Leipzig, Leipzig, Germany; 82 IFB AdiposityDiseases, University of Leipzig, Leipzig, Germany; 83 Department of Epidemiology, University of Groningen, University Medical Center Groningen, Groningen, the Netherlands; 84 Faculty of Medicine, University of Split, Split, Croatia; 85 Department of Clinical Epidemiology and Biostatistics, Academic Medical Center, University of Amsterdam, Amsterdam, The Netherlands; 86 Laboratory of Clinical Investigation, National Institute of Aging, Baltimore, MD USA; 87 Pediatric Research Center, Department of Women’s & Child Health, University of Leipzig, Leipzig, Germany; 88 Department of Medicine, University of Eastern Finland and Kuopio University Hospital, Kuopio, Finland; 89 Department of Dietetics-Nutrition, Harokopio University, Athens, Greece; 90 Center for Biomedicine, European Academy Bozen/Bolzano (EURAC) (Affiliated Institute of the University of LübeckLübeckGermany), Bolzano, Italy; 91 Kuopio Research Institute of Exercise Medicine, Kuopio, Finland; 92 Department of Clinical Physiology and Nuclear Medicine, Kuopio University Hospital, Kuopio, Finland; 93 Geriatric Unit, Azienda Sanitaria Firenze (ASF), Florence, Italy; 94 Institute of Epidemiology, Helmholtz Zentrum München, German Research Center for Environmental Health, Neuherberg, Germany; 95 Department of Epidemiology and Prevention, Division of Public Health Sciences, Wake Forest University School of Medicine, Winston-Salem, NC USA; 96 Department of Epidemiology, Center for Aging and Population Health, University of Pittsburgh, Pittsburgh, PA USA; 97 Department of Medical Sciences, Uppsala University, Akademiska sjukhuset, Uppsala, Sweden; 98 Pat McPherson Centre for Pharmacogenetics and Pharmacogenomics, Division of Molecular and Clinical Medicine, Ninewells Hospital and Medical School, University of Dundee, Dundee, UK; 99 Estonian Genome Center, University of Tartu, Tartu, Estonia; 100 Department of Epidemiology and Public Health, University College London, London, UK; 101 University of Essex, Wivenhoe Park, Colchester, Essex UK; 102 Institute of Clinical Diabetology, German Diabetes Center, Leibniz Center for Diabetes Research at Heinrich Heine University Düsseldorf, Düsseldorf, Germany; 103 Division of Endocrinology and Diabetology, Medical Faculty, Heinrich Heine University Düsseldorf, Düsseldorf, Germany; 104 Department of Public Health Solutions, Finnish Institute for Health and Welfare, P.O. Box 30, Helsinki, FI-00271 Finland; 105 Department of Medicine, University of Helsinki and Helsinki University Central Hospital, P.O. Box 340, Haartmaninkatu 4, Helsinki, FI-00029 Finland; 106 Minerva Foundation Institute for Medical Research, Biomedicum 2U, Tukholmankatu 8, Helsinki, FI-00290 Finland; 107 Division of Cardiovascular Epidemiology, Institute of Environmental Medicine, Karolinska Institutet, Stockholm, Sweden; 108 Department of Internal Medicine, University of Groningen, University Medical Center Groningen, Groningen, The Netherlands; 109 Institute of Public Health and Clinical Nutrition, University of Eastern Finland, Kuopio, Finland; 110 Dept of Cardiology, Leiden University Medical Center, Leiden, The Netherlands; 111 Netherlands Heart Institute, Utrecht, The Netherlands; 112 Institute of Cardiovascular and Medical Sciences, University of Glasgow, Glasgow, UK; 113 Department of Government Services, Finnish Institute for Health and Welfare, Helsinki, Finland; 114 PEDEGO Research Unit, Medical Research Center Oulu, Oulu University Hospital and University of Oulu, Oulu, Finland; 115 IMM Center for Human Genetics, University of Texas Health Science Center at Houston, Houston, TX USA; 116 Division of Epidemiology, School of Public Health, University of Texas Health Science Center at Houston, Houston, TX USA; 117 Division of Epidemiology and Community Health, School of Public Health, University of Minnesota, Minneapolis, MiI USA; 118 Department of Medical Epidemiology and Biostatistics, Karolinska Institutet, Stockholm, Sweden; 119 Department of Clinical Sciences, Diabetes and Endocrinology Research Unit, University Hospital Malmö, Lund University, Malmö, Sweden; 120 Department of Endocrinology, University of Groningen, University Medical Center Groningen, Groningen, The Netherlands; 121 General Medicine Division, Massachusetts General Hospital, Boston, MA USA; 122 Department of Medicine, Harvard Medical School, Boston, MA USA; 123 Department of Vascular Medicine, Amsterdam UMC, Amsterdam, The Netherlands; 124 Novo Nordisk A/S, Copenhagen, Denmark; 125 Finnish Institute for Health and Welfare, Diabetes Prevention Unit, Helsinki, Finland; 126 Department of Medicine, Division for Prevention and Care of Diabetes, Faculty of Medicine Carl Gustav Carus, Technische Universität Dresden, Dresden, Germany; 127 Department for Prevention and Care of Diabetes, Faculty of Medicine Carl Gustav Carus, Technische Universität Dresden, Dresden, Germany; 128 Paul Langerhans Institute Dresden of the Helmholtz Center Munich at University Hospital and Faculty of Medicine, TU Dresden, Dresden, Germany; 129 German Center for Diabetes Research (DZD e.V.), Neuherberg, Germany; 130 Cardiology Group, Frankfurt-Sachsenhausen, Germany; 131 Lee Kong Chian School of Medicine, Nanyang Technological University Singapore and Imperial College London, Singapore, Singapore; 132 Synlab Academy, Synlab Holding Deutschland GmbH, Mannheim, Germany; 133 Princess Al-Jawhara Al-Brahim Centre of Excellence in Research of Hereditary Disorders (PACER-HD, King Abdulaziz University, Jeddah, Saudi Arabia; 134 Institute of Biomedicine/Physiology, University of Eastern Finland, Kuopio Campus, Kuopio, Finland; 135 Faculty of Medicine, Center for Life Course Health Research, University of Oulu, Oulu, Finland; 136 Unit of General Practice, Oulu University Hospital, Oulu, Finland; 137 Finnish Diabetes Association, Tampere, Finland; 138 Pirkanmaa Hospital District, Tampere, Finland; 139 Diabetes and Obesity Research Institute, Cedars-Sinai Medical Center, Los Angeles, CA USA; 140 Department of Chronic Disease Prevention, Finnish Institute for Health and Welfare, Helsinki, Finland; 141 Department of Public Health, University of Helsinki, Helsinki, Finland; 142 Centre for Vascular Prevention, Danube-University Krems, Krems, Austria; 143 Diabetes Research Group, King Abdulaziz University, Jeddah, Saudi Arabia; 144 Department of Nutrition, Harvard School of Public Health, Boston, MA USA; 145 Department of Public Health & Clinical Medicine, Units of Medicine and Nutritional Research, Umeå University, Umeå, Sweden; 146 Inserm, CESP Center for Research in Epidemiology and Public Health, U1018 Villejuif, France; 147 Univ Paris-Saclay, Univ Paris Sud, UVSQ, UMRS 1018, UMRS 1018, Villejuif, France; 148 Centre for Medical Systems Biology, Leiden, The Netherlands; 149 Hannover Unified Biobank, Hannover Medical School, Hannover, Germany; 150 Institute of Human Genetics, Hannover Medical School, Hannover, Germany; 151 Clinical Research Branch, National Institute on Aging, Baltimore, MD USA; 152 Department of Neurology, General Central Hospital, Bolzano, Italy; 153 Department of Neurology, University of Lübeck, Lübeck, Germany; 154 Genetics of Complex Traits, Peninsula Medical School, University of Exeter, Exeter, UK; 155 The Regeneron Genetics Center, Regeneron Pharmaceuticals, Tarrytown, NY USA; 156 School of Public Health, University of Adelaide, Adelaide, Australia; 157 U872 Institut National de la Santé et de la Recherche Médicale, Centre de Recherche des Cordeliers, 75006 Paris, France; 158 Geriatric Epidemiology Section, Laboratory of Epidemiology, Demography, and Biometry, National Institute on Aging, Bethesda, MD USA; 159 Department of Genetics, University Medical Center Groningen, University of Groningen, Groningen, The Netherlands; 160 MRC Integrative Epidemiology Unit (IEU), University of Bristol, Bristol, UK; 161 The Charles Bronfman Institute for Personalized Medicine, Icahn School of Medicine at Mount Sinai, New York, NY USA; 162 Finnish Institute for Health and Welfare, Helsinki, Finland; 163 Endocrinology, Abdominal Centre, University of Helsinki and Helsinki University Hospital, Helsinki, Finland; 164 Research Program for Clinical and Molecular Metabolism, University of Helsinki and Folkhälsan Research Center, Helsinki, Finland; 165 Netherlands Consortium for healthy ageing, the Hague, The Netherlands; 166 Department of Epidemiology, Harvard T.H. Chan School of Public Health, Boston, USA; 167 Faculty of Medicine University of Iceland, Reykjavik, Iceland; 168 Department of Epidemiology, University of Washington, Seattle, WA USA; 169 Department of Medicine, University Hospital Lausanne, Lausanne, Switzerland; 170 Cardiothoracic Surgery Unit, Department of Molecular Medicine and Surgery, Karolinska Institutet, Stockholm, Sweden; 171 Department of Epidemiology and Biostatistics and HPA-MRC Center, School of Public Health, Imperial College London, London, UK; 172 Institue of Health Sciences, University of Oulu, Oulu, Finland; 173 Faculty of Medicine, University of Iceland, Reykjavík, Iceland; 174 Department of Cardiology, Karolinska University Hospital Solna, Stockholm, Sweden; 175 Department of Statistics, University of Oxford, Oxford, UK; 176 Oxford National Institute for Health Research Biomedical Research Centre, Churchill Hospital, Oxford, UK; 177 Department of Genetic Medicine and Development, University of Geneva Medical School, Geneva, Switzerland; 178 Big Data Institute, Li Ka Shing Centre for Health Information and Discovery, University of Oxford, Oxford, UK; 179 School of Biosciences and Medicine, Department of Clinical and Experimental Medicine, University of Surrey, Guildford, UK; 180 Department of Clinical Science, University of Bergen, Bergen, Norway; 181 Department of Preventive Medicine, Keck School of Medicine of USC, Los Angeles, CA USA; 182 Department of Physiology & Neuroscience, Keck School of Medicine of USC, Los Angeles, CA USA; 183 USC Diabetes and Obesity Research Institute, Los Angeles, CA USA; 184 Department of Medicine, Division of Cardiovascular Medicine, Stanford University School of Medicine, Stanford, CA USA; 185 Stanford Cardiovascular Institute, Stanford University, Stanford, CA 94305 USA; 186 Diabetes Research Center, Diabetes Unit, Massachusetts General Hospital, Boston, MA USA; 187 Department of Biostatistics, Boston University School of Public Health, Boston, MA USA; 188 University of Cambridge Metabolic Research Laboratories and NIHR Cambridge Biomedical Research Centre, Wellcome Trust-MRC Institute of Metabolic Science, Cambridge, UK; 189 Department of Biostatistics, University of Liverpool, Liverpool, UK; 190 Centre for Genetics and Genomics Versus Arthritis, Centre for Musculoskeletal Research, The University of Manchester, Manchester, UK; 191 Institute of Biochemistry and Genetics, Ufa Federal Research Centre Russian Academy of Sciences, Ufa, Russian Federation; 192 Present Address: Genentech, 340 Point San Bruno Boulevard, South San Francisco, CA 94080 USA; 193 Present Address: Exeter Centre of ExcEllence in Diabetes (ExCEED), University of Exeter Medical School, Exeter, UK

**Keywords:** Genome-wide association studies, Quantitative trait, Diagnostic markers, Pre-diabetes, Type 2 diabetes

Correction to: *Nature Communications* 10.1038/s41467-020-19366-9, published online 5 January 2021.

The original version of this Article contained an error in Fig. 2, in which panels a and b were inadvertently swapped.

This has now been corrected in the PDF and HTML versions of the Article.

