# Supplementary material for: Sex-dimorphic genetic effects and novel loci for fasting glucose and insulin variability
Source: Nat Commun. 2021 Jan 5;12:24. doi: 10.1038/s41467-020-19366-9 (PMC7785747; doi:10.1038/s41467-020-19366-9)
Supplement: Supplementary file 1 — Supplementary Information [file 41467_2020_19366_MOESM1_ESM.pdf]

Supplementary Information

# Sex-dimorphic genetic effects and novel loci for fasting glucose and insulin variability.

*Lagou, Mägi, Hottenga et al*

## Table of contents

**SUPPLEMENTARY NOTE 1 Additional acknowledgements**

**SUPPLEMENTARY FIGURES**

**Supplementary Figure 1. Regional and tissue expression plots.**

**Supplementary Figure 2. Regional plots for novel loci with sex-combined effects on FG.**

**Supplementary Figure 3. GARFIELD enrichment analysis.**

**Supplementary Figure 4. Power of tests for detecting sex heterogeneity through simulations.**

**Supplementary Figure 5. Tissue expression of genes within the novel *ZNF12* locus.**

## SUPPLEMENTARY NOTE 1 Additional acknowledgements

**Age, Gene Environment Susceptibility Reykjavik Study (AGES)**

This study has been funded by NIH contract N01-AG-1-2100 and HHSN271201200022C, the NIA Intramural Research Program, Hjartavernd (the Icelandic Heart Association), and the Althingi (the Icelandic Parliament). The study is approved by the Icelandic National Bioethics Committee, VSN: 00-063. The researchers are indebted to the participants for their willingness to participate in the study.

**The Avon Longitudinal Study of Parents and Children (ALSPAC)**

We are extremely grateful to all the families who took part in this study, the midwives for their help in recruiting them, and the whole ALSPAC team, which includes interviewers, computer and laboratory technicians, clerical workers, research scientists, volunteers, managers, receptionists and nurses. The UK Medical Research Council and Wellcome (Grant ref: 217065/Z/19/Z) and the University of Bristol provide core support for ALSPAC. This publication is the work of the authors and Beate St Pourcain and Nicholas Timpson will serve as guarantors for the contents of this paper. A comprehensive list of grants funding is available on the ALSPAC website (http://www.bristol.ac.uk/alspac/external/documents/grant-acknowledgements.pdf). GWAS data was generated by Sample Logistics and Genotyping Facilities at Wellcome Sanger Institute and LabCorp (Laboratory Corporation of America) using support from 23andMe.

**AMC-PAS Prospective Cohort Study**

The AMC-PAS study was largely initiated by M.D. Trip MD, PhD and S.Sivapalaratnam MD, PhD, and we are indebted to the participants of this study.

**The Amish Family Osteoporosis Study (HAPI and LS)**

This work was supported by research grants R01 088119, R01 AR046838, U01 HL72515, and R01 AG18728. Additional support was provided by the University of Maryland General Clinical Research Center, Grant M01 RR 16500, Mid-Atlantic Nutrition Obesity Research Center Grant P30 DK072488, General Clinical Research Centers Program, National Center for Research Resources (NCRR), NIH, and the Baltimore Veterans Administration Geriatric Research and Education Clinical Center (GRECC). Dr Montasser was supported by T32 training Grant AG000219. We gratefully acknowledge our Amish liaisons and field workers and the extraordinary cooperation and support of the Amish community without which these studies would not have been possible.

**Atherosclerosis Risk in Communities Study (ARIC)**

The Atherosclerosis Risk in Communities Study is carried out as a collaborative study supported by National Heart, Lung, and Blood Institute contracts (HHSN268201100005C, HHSN268201100006C, HHSN268201100007C, HHSN268201100008C, HHSN268201100009C, HHSN268201100010C, HHSN268201100011C, and HHSN268201100012C), R01HL087641, R01HL59367 and R01HL086694; National Human Genome Research Institute contract U01HG004402; and National Institutes of Health contract HHSN268200625226C. The authors thank the staff and participants of the ARIC study for their important contributions. Infrastructure was partly supported by Grant Number UL1RR025005, a component of the National Institutes of Health and NIH Roadmap for Medical Research.

**The ASCOT Study**

This work was supported by Pfizer, New York, NY, USA, for the ASCOT study and the collection of the ASCOT DNA repository; by Servier Research Group, Paris, France; and by Leo Laboratories, Copenhagen, Denmark. We thank all ASCOT trial participants, physicians, nurses, and practices in the participating countries for their important contribution to the study. In particular we thank Clare Muckian and David Toomey for their help in DNA extraction, storage, and handling. The genotyping was funded by a Wellcome Trust Strategic Award (no. 083948). PBM wishes to acknowledge the support of the NIHR Cardiovascular Biomedical Research Unit at Barts and Queen Mary University of London, UK.

**The Baltimore Longitudinal Study of Aging (BLSA)**

The BLSA was supported in part by the Intramural Research Program of the NIH, National Institute on Aging. A portion of that support was through a R&D contract with MedStar Research Institute.

**The Busselton Health Study (BHS)**

The Busselton Health Study (BHS) acknowledges the generous support for the 1994/5 follow-up study from Healthway, Western Australia and the numerous Busselton community volunteers who assisted with data collection and the study participants from the Shire of Busselton. The Busselton Health Study is supported by The Great Wine Estates of the Margaret River region of Western Australia.

**The Cardiovascular Health Study (CHS)**

The Cardiovascular Health Study research was supported by National Heart, Lung, and Blood Institute contracts N01-HC-85239, N01-HC-85079 through N01-HC-85086; N01-HC-35129, N01 HC-15103, N01 HC-55222, N01-HC-75150, N01-HC-45133 and NHLBI grants HL080295, HL075366, HL087652, HL105756 with additional contribution from the National Institute of Neurological Disorders and Stroke. Additional support was provided through AG-023629, AG-15928, AG-20098, and AG-027058 from the National Institute on Aging. See also http://www.chs-nhlbi.org/pi.htm. DNA handling and genotyping was supported in part by the Clinical Translational Science Institute grant UL1RR033176 to the Cedars-Sinai General Clinical Research Center Genotyping core and National Institute of Diabetes and Digestive and Kidney Diseases grant DK063491 to the Southern California Diabetes Endocrinology Research Center.

**The CoLaus Study**

The CoLaus study was and is supported by research grants from GlaxoSmithKline, the Faculty of Biology and Medicine of Lausanne, and the Swiss National Science Foundation (grants 3200B0–105993, 3200B0-118308, 33CSCO-122661, 33CS30-139468 and 33CS30-148401).

**The Croatia Study**

The work was supported by European Union framework program 6 EUROSPAN project (contract no. LSHG-CT-2006-018947); grant ~216-1080315-0302 (to IR) from the Croatian Ministry of Science, Education and Sport; Studies carried out in the Croatian island of Vis were supported by Medical Research Council UK. The authors collectively thank a large number of individuals for their help in organizing, planning and carrying out the field work related to the project: Professor Pavao Rudan and staff of the Institute for Anthropological Research in Zagreb, Croatia; Professor Stipan Jankovic and staff at the University of Split Medical School; Professor Ariana Vorko-Jovic and staff and medical students of the Andrija Stampar School of Public Health of the Faculty of Medicine, University of Zagreb, Croatia; Dr Branka Salzer from the biochemistry lab "Salzer", Croatia; local general practitioners and nurses; and the employees of several other Croatian institutions who participated in the field work. including but not limited to the University of Rijeka, Croatia; Croatian Institute of Public Health; Institutes of Public Health in Split and Dubrovnik, Croatia. SNP Genotyping of the Vis samples was carried out by the Genetics Core Laboratory at the Clinical Research Facility, University of Edinburgh.

**The FIN-D2D-2007 Study**

The FIN-D2D study has been financially supported by the hospital districts of Pirkanmaa, North Ostrobothnia Hospital District, South Ostrobothnia, and Central Finland, the Finnish National Public Health Institute (current Finnish Institute for Health and Welfare), the Finnish Diabetes Association, the Ministry of Social Affairs and Health in Finland, the Academy of Finland (grant number 129293),Commission of the European Communities, Directorate C-Public Health (grant agreement no. 2004310) and Finland's Slottery Machine Association.

**The Diabetes Prevention Study (DPS)**

The DPS has been financially supported by grants from the Academy of Finland (117844 and 40758, 211497, and 118590), The EVO funding of the Kuopio University Hospital from Ministry of Health and Social Affairs (5254), Finnish Funding Agency for Technology and Innovation (40058/07), Nordic Centre of Excellence on Systems biology in controlled dietary interventions and cohort studies, SYSDIET (070014), The Finnish Diabetes Research Foundation, Yrjö Jahnsson

Foundation (56358), Sigrid Juselius Foundation, Juho Vainio Foundation and TEKES grants 70103/06 and 40058/07.

**The DR's EXTRA Study**

The DR's EXTRA Study was supported by grants from the Ministry of Education and Culture of Finland (627;2004-2011), Academy of Finland (102318; 123885), Kuopio University Hospital, Finnish Diabetes Association, Finnish Heart Association, Päivikki and Sakari Sohlberg Foundation, European Commission FP6 Integrated Project (EXGENESIS), LSHM-CT-2004-005272, City of Kuopio and Social Insurance Institution of Finland (4/26/2010).

**The population based Metabolic Syndrome in Men Study (METSIM)**

The METSIM study was funded by the Academy of Finland (grants no. 77299 and 124243).

**The Diabetes Epidemiology: Collaborative analysis Of Diagnostic criteria in Europe S*tudy* (DECODE)**

We thank the individuals who participated in the study and whose contribution made this work possible. The research performed at deCODE genetics was part funded through the European Community's Seventh Framework Programme (FP7/2007-2013), ENGAGE project, grant agreement HEALTH-F4-2007- 201413.

**The DESIR Study**

We thank all the participants of the D.E.S.I.R study, Elodie Eury and Stéphane Lobbens for technical support for the genotyping, Jean Tichet and Michel Marre from the D.E.S.I.R study. The genotyping was supported by the “Conseil Régional Nord-Pas-de-Calais Fonds européen de développement économique et regional” CPER axe Cartdiodiabète 2010-2011 grant to NB-N.

**The Diabetes Genetics Initiative Study (DGI)**

We thank the study participants who made this research possible. We thank colleagues in the Broad Genetic Analysis and Biological Samples Platforms for their expertise and contributions to genotyping, data and sample management, and analysis. The initial GWAS genotyping was supported by Novartis (to D.A.); support for additional analysis and genotyping in this report was provided by funding from the Broad Institute of Harvard and MIT, by the Richard and Susan Smith Family Foundation/American Diabetes Association Pinnacle Program Project Award (to D.A.), and by a Freedom to Discovery award of the Foundation of Bristol Myers Squibb (to D.A.). M.J.D. and D.A. acknowledge support from US National Institutes of Health/National Heart, Lung, and Blood Institute grant (U01 HG004171). D.A. was a Burroughs Wellcome Fund Clinical Scholar in Translational Research and is a Distinguished Clinical Scholar of the Doris Duke Charitable Foundation. D.A. and B.F.V. acknowledges sponsored research funding from Pfizer, Inc. S.R. is supported by an NIH Career Development Award (1K08AR055688). A.V.S acknowledges support from the American Diabetes Association (Award No.: 7-08-MN-OK) A.L.E. was supported as a research fellow by the Sarnoff Cardiovascular Research Fellowship. L.G., T.T., B.I. and the Botnia Study are principally supported by the Sigrid Juselius Foundation, the Finnish Diabetes Research Foundation, The Folkhalsan Research Foundation and Clinical Research Institute HUCH Ltd; work in Malmö, Sweden was also funded by a Linne grant from the Swedish Research Council (349 2006-237P) and the Skaraborg Institute, Skövde. Additional genotyping for stage 2 samples in Malmö was supported by grants from the Swedish Research Council (LG) and the Wallenberg Foundation. We thank the Botnia and Skara research teams for clinical contributions.

**The DIAGEN Study**

The presented study was supported by the Commission of the European Communities, Directorate C - Public Health and Risk Assessment, Health & Consumer Protection, Grant Agreement number - 2004310 and by the Dresden University of Technology Funding Grant, Med Drive. We are grateful to all of the patients who cooperated in this study and to their referring physicians and diabetologists in Saxony.

**The DIetary, Lifestyle, and Genetic determinants of Obesity and Metabolic syndrome Study (DILGOM)**

Etelä-Pohjanmaa Hospital District, Pohjois-Pohjanmaa Hospital District, Keski-Suomi Hospital District, Pirkanmaa Hospital District, Pohjois-Savo Hospital District. S.M. Was supported by Academy of Finland (# 136895 and 141005).

**The Genetics of Diabetes Audit and Research in Tayside Scotland Study (DUNDEE/GODARTS, REPLICATION_DUNDEE)**

This study was funded by the Wellcome Trust (084727/Z/08/Z, 085475/Z/08/Z, 085475/B/08/Z) and as part of the EU IMI-SUMMIT program. We acknowledge the support of the Health Informatics Centre, University of Dundee for managing and supplying the anonymised data and NHS Tayside, the original data owner. We are grateful to all the participants who took part in the Go-DARTS study, to the general practitioners, to the Scottish School of Primary Care for their help in recruiting the participants, and to the whole team, which includes interviewers, computer and laboratory technicians, clerical workers, research scientists, volunteers, managers, receptionists, and nurses. The replication study was funded by the Wellcome Trust, Tenovus Tayside and the Medical Research Council UK. Research was supported by the Wellcome Trust funding grants 090532, 098381; and by FP7 ENGAGE (HEALTH-F4-2007- 201413).

**The Edinburgh Artery Study (EAS)**

EAS was supported by the British Heart Foundation. Genotyping was supported by a grant from the Chief Scientist Office, Scotland and performed at the Wellcome Trust Clinical Research Facility in Edinburgh.

**The Estonian Genome Center of the University of Tartu Study (EGCUT)**

EGCUT received financing by FP7 grants (201413, 245536), also received targeted financing from Estonian Research Council [IUT20-60, IUT24-6, PUT1660 to T.E.]; European Union Horizon 2020 [692145]; European Union through the European Regional Development Fund [2014-2020.4.01.15-0012 GENTRANSMED];National Institute of Health R01 [MP1GV17428]. We acknowledge EGCUT and Estonian Biocentre personnel, especially Ms. S. Smith and Mr V. Soo.

**The Ely and Fenland Studies**

The Fenland Study is funded by the Wellcome Trust and the Medical Research Council. We are grateful to all the volunteers for their time and help, and to the General Practitioners and practice staff for help with recruitment. We thank the Fenland Study. Genotyping in the Fenland and Ely studies was supported in part by an MRC-GSK pilot programme grant (ID 85374).

**The Erasmus Rucphen Family Study (ERF)**

The ERF study was supported by grants from the Netherlands Organization for Scientific Research (NWO; Pioneergrant), Erasmus Medical Center, the Centre for Medical Systems Biology (CMSB),

and the Netherlands Kidney Foundation. We are grateful to all patients and their relatives, general practitioners and specialists for their contributions and to P. Veraart for her help in genealogy, Jeannette Vergeer for her supervision of the laboratory work and P. Snijders for his help in data collection.

**The Family Heart Study (FamHS)**

The Family Heart Study (FamHS) work was supported in part by NIH's NHLBI and NIDDK grants 5R01HL08770003, 5R01HL08821502, 5R01DK07568102 and 5R01DK06833603.

**The Framingham Heart Study (FHS)**

This research was conducted in part using data and resources from the Framingham Heart Study of the National Heart Lung and Blood Institute of the National Institutes of Health and Boston University School of Medicine. The analyses reflect intellectual input and resource development from the Framingham Heart Study investigators participating in the SNP Health Association Resource (SHARe) project. This work was partially supported by the National Heart, Lung and Blood Institute's Framingham Heart Study (Contract No. N01-HC-25195 and HHSN268201500001I) and its contract with Affymetrix, Inc for genotyping services (Contract No. N02-HL-6-4278). Also supported by National Institute for Diabetes and Digestive and Kidney Diseases (NIDDK) R01 DK078616 to Drs. Meigs, Dupuis and Florez, and NIDDK K24 DK080140 to Dr. Meigs.

**The STANISLAS study (FrenchAdultControl, FrenchAdultObese, FrenchYoungControl, FrenchYoungObese)**

The authors acknowledge all investigators for the recruitment and examinations of the STANISLAS Family Study, supported by the “Caisse Nationale d’Assurance Maladie des Travailleurs Salariés” (CNAM), the “Institut National de la Santé et de la Recherche Médicale” (INSERM), the “Région Lorraine”, the “Communauté Urbaine du Grand Nancy”, the Henri Poincaré University, Nancy 1, and the “BioIntelligence” project. We thank Jean Tichet and Michel Marre from the D.E.S.I.R study. We thank Jacques Weill for recruitement of obesity families at Jeanne de Flandres Hospital, Marianne Deweirder and Frédéric Allegaert for DNA management and Stefan Gaget for database managements. We thank Jacques Weill for recruitement of obesity families at Jeanne de Flandres Hospital, Marianne Deweirder and Frédéric Allegaert for DNA management and Stefan Gaget for database managements.

**The Finland-United States Investigation of NIDDM Genetics Study (FUSION)**

This work was funded by NIH grants U01 DK062370, R01-HG000376, R01-DK072193, and NIH intramural project number ZIA HG000024. Genome-wide genotyping was conducted by the Johns Hopkins University Genetic Resources Core. Facility SNP Center at the Center for Inherited Disease Research (CIDR), with support from CIDR NIH contract number N01-HG-65403.

**Genetic Epidemiology Network of Arteriopathy study (GENOA)**

Support for GENOA was provided by the National Heart, Lung and Blood Institute (HL054457, HL054464, HL054481, HL119443, and HL087660) of the National Institutes of Health. We thank Eric Boerwinkle, PhD from the Human Genetics Center and Institute of Molecular Medicine and Division of Epidemiology, University of Texas Health Science Center, Houston, Texas, USA and Julie Cunningham, PhD from the Department of Health Sciences Research, Mayo Clinic College of Medicine, Rochester, MN, USA for their help with genotyping.

**The GenomEUtwin Study**

We acknowledge support from the European Commission under 'Quality of Life and Management of the Living Resources' of the Fifth Framework Program (GenomEUtwin QLG2-CT-2002-01254). Supported by "ENGAGE – European Network for Genetic and Genomic Epidemiology, FP7-HEALTH-F4-2007, grant agreement number 201413".

**The GLACIER Study**

The GLACIER Study was funded by grants from the Swedish Diabetes Association, Swedish Heart-Lung Foundation, Swedish Research Council, Medical Research Foundation of Umeå University, and Novo Nordisk (all to PWF). We thank the participants for their outstanding contributions to the GLACIER Study. We also thank the staff of the Umeå Medical Biobank, especially Göran Hallmans, Åsa Agren, John Hutilainen, and Ann-Marie Ahren for data retrieval and organisation and Kerstin Enqusit and Tore Johansson for expert assistance with DNA extraction and plating. The GLACIER Study is nested within the Västerbottens Intervention Project (VIP); we thank the staff of the VIP Study for phenotype data collection, particularly Lars Wennehall who leads the VIP Study. Inês Barroso acknowledges funding from the Wellcome Trust grant WT098051, United Kingdom NIHR Cambridge Biomedical Research Centre and the MRC Centre for Obesity and Related Metabolic Diseases. We would like to thank Emma Gray, Douglas Simpkin, Sarah Hunt, Sarah Edkins and staff of the WTSI Sample Logistics, Genotyping and Variation Informatics Facilities.

**The Health, Aging and Body Composition Study (HABC)**

The Health, Aging and Body Composition Study was supported by National Institute on Aging (NIA) contracts N01AG62101, N01AG62103, and N01AG62106, and in part by the Intramural Research Program of the NIH, NIA. The genome-wide association study was funded by NIA grant 1R01AG032098-01A1 to Wake Forest University Health Sciences and genotyping services were provided by the Center for Inherited Disease Research (CIDR). CIDR is fully funded through a federal contract from the National Institutes of Health to The Johns Hopkins University, contract number HHSN268200782096C.

**The Health2000 Study**

MP was supported by Sigrid Juselius Foundation, Finnish Foundation for Cardiovascular Research and Academy of Finland, grant number 129322. VS was supported by the Finnish Foundation for Cardiovascular research.

**The InChianti Study**

JRBP is funded by a Sir Henry Wellcome Postdoctoral Research Fellowship (092447/Z/10/Z). The InCHIANTI study baseline (1998-2000) was supported as a "targeted project" (ICS110.1/RF97.71) by the Italian Ministry of Health and in part by the U.S. National Institute on Aging (Contracts: 263 MD 9164 and 263 MD 821336); the InCHIANTI Follow-up 1 (2001-2003) was funded by the U.S. National Institute on Aging (Contracts: N.1-AG-1-1 and N.1-AG-1-2111); the InCHIANTI Follow-ups 2 and 3 studies (2004-2010) were financed by the U.S. National Institute on Aging (Contract: N01-AG-5-0002);supported in part by the Intramural research program of the National Institute on Aging, National Institutes of Health, Baltimore, Maryland.

**KORA F4**

The KORA study was initiated and financed by the Helmholtz Zentrum München—German Research Center for Environmental Health, which is funded by the German Federal Ministry of

Education and Research (BMBF) and by the State of Bavaria. Furthermore, KORA research was supported within the Munich Center of Health Sciences (MC-Health), Ludwig-Maximilians-Universität, as part of LMUinnovativ. This study was supported in part by a grant from the German Federal Ministry of Education and Research (BMBF) to the German Center for Diabetes Research (DZD). The German Diabetes Center is funded by the German Federal Ministry of Health (BMG) and the Ministry of Culture and Science of the State North Rhine-Westphalia. We gratefully acknowledge the contribution of all members of field staff conducting the KORA F4 study.

**The KORCULA Study**

The work is supported by European Union framework program 6 EUROSPAN project (contract no. LSHG-CT-2006-018947); grant ~216-1080315-0302 (to IR) from the Croatian Ministry of Science, Education and Sport; Studies carried out in the Croatian island of Korcula were supported by Medical Research Council UK. The authors collectively thank a large number of individuals for their help in organizing, planning and carrying out the field work related to the project: Professor Stipan Jankovic and staff at the University of Split Medical School; Dr Branka Salzer from the biochemistry lab "Salzer", Croatia; local general practitioners and nurses; and the employees of several other Croatian institutions who participated in the field work. including but not limited to the University of Rijeka, Croatia; Croatian Institute of Public Health; Institutes of Public Health in Split and Dubrovnik, Croatia. SNP Genotyping of the Korcula samples was carried out by Helmholtz Zentrum München, GmbH, Neuherberg, Germany.

**Integrated Research and Treatment Center Leipzig (IFB) study (LEIPZIG_ADULT_IBF, LEIPZIG_CHILHOOD_IBF, REPLICATION_LEIPZIG)**

This work was supported by grants from Integrated Research and Treatment Centre (IFB) Adiposity Diseases (K7-36 to MS and AK), from the German Research Foundation (SFB-1052 ''Obesity mechanisms'' B01, C05). We are grateful to all the patients and families for contributing to the study. We highly appreciate the support of the Obesity Team and Auxo Team of the Leipzig University Children´s Hospital for management of the patients and to the Pediatric Research Center Lab Team for support with DNA banking.

**The Lifelines Study (REPLICATION_LIFELINES)**

The Lifelines Cohort Study, and generation and management of GWAS genotype data for the Lifelines Cohort Study is supported by the Netherlands Organization of Scientific Research NWO (grant 175.010.2007.006), the Economic Structure Enhancing Fund (FES) of the Dutch government, the Ministry of Economic Affairs, the Ministry of Education, Culture and Science, the Ministry for Health, Welfare and Sports, the Northern Netherlands Collaboration of Provinces (SNN), the Province of Groningen, University Medical Center Groningen, the University of Groningen, Dutch Kidney Foundation and Dutch Diabetes Research Foundation. The authors wish to acknowledge the services of the Lifelines Cohort Study, the contributing research centers delivering data to Lifelines, and all the study participants.

**The Ludwigshafen Risk and Cardiovascular Health Study (LURIC)**

LURIC received funding through the 6th Framework Program (integrated project Bloodomics, grant LSHM-CT-2004-503485) and 7th of Framework Program (integrated project AtheroRemo, Grant Agreement number 201668) of the European Union. The authors extend appreciation to the participants of the LURIC study without their collaboration this article would not have been written. We thank the LURIC study team either temporarily or permanently involved in patient

recruitment, sample and data handling, and the laboratory staff at the Ludwigshafen General Hospital and the Universities of Freiburg and Ulm, Germany.

**The Northern Finland Birth Cohort Studies (NFBC66/NFBC86)**

NFBC1986(1966) received financial support from the Academy of Finland (project grants 104781, 120315, 129269, 1114194, Center of Excellence in Complex Disease Genetics and SALVE), University Hospital Oulu, Biocenter, University of Oulu, Finland (75617), the European Commission (EURO-BLCS, Framework 5 award QLG1-CT-2000-01643), NHLBI grant 5R01HL087679-02 through the STAMPEED program (1RL1MH083268-01), NIH/NIMH (5R01MH63706:02), ENGAGE project and grant agreement HEALTH-F4-2007-201413, the Medical Research Council, UK (G0500539, G0600705, PrevMetSyn/SALVE) and the Wellcome Trust (project grant GR069224), UK. The DNA extractions, sample quality controls, biobank up-keeping and aliquotting was performed in the National Public Health Institute, Biomedicum Helsinki, Finland and supported financially by the Academy of Finland and Biocentrum Helsinki. We thank Professor (emeriti) Paula Rantakallio (launch of NFBC1966 and 1986), and Ms Outi Tornwall and Ms Minttu Jussila (DNA biobanking).

**The Netherlands Twin Register and Netherlands Study of Depression And Anxiety (NTRNESDA and REPLICATION_NTR2)**

Funding was obtained from the Netherlands Organization for Scientific Research (NWO) and The Netherlands Organisation for Health Research and Development (ZonMW) grants 904-61-090, 985-10-002, 912-10-020, 904-61-193,480-04-004, 463-06-001, 451-04-034, 400-05-717, Addiction-31160008, 016-115-035, 481-08-011, 400-07-080, 056-32-010, Middelgroot-911-09-032, OCW_NWO Gravity program –024.001.003, NWO-Groot 480-15-001/674, Center for Medical Systems Biology (CSMB, NWO Genomics), NBIC/BioAssist/RK(2008.024), Biobanking and Biomolecular Resources Research Infrastructure (BBMRI –NL, 184.021.007 and 184.033.111); Spinozapremie (NWO- 56-464-14192), KNAW Academy Professor Award (PAH/6635) and University Research Fellow grant (URF) to DIB; Amsterdam Public Health research institute (former EMGO+), Neuroscience Amsterdam research institute (former NCA) ; the European Science Foundation (ESF, EU/QLRT-2001-01254), the European Community's Seventh Framework Program (FP7- HEALTH-F4-2007-2013, grant 01413: ENGAGE and grant 602768: ACTION); the European Research Council (ERC Starting 284167, ERC Consolidator 771057, ERC Advanced 230374), Rutgers University Cell and DNA Repository (NIMH U24 MH068457-06), the National Institutes of Health (NIH, R01D0042157-01A1, R01MH58799-03, MH081802, DA018673, R01 DK092127-04, Grand Opportunity grants 1RC2 MH089951, and 1RC2 MH089995); the Avera Institute for Human Genetics, Sioux Falls, South Dakota (USA), the Genetic Association Information Network (GAIN) of the Foundation for the National Institutes of Health, the Geestkracht program of the Netherlands Organization for Health Research and Development (Zon-MW, grant number 10-000-1002), the Swedish Research Council, the Swedish Heart-Lung Foundation, the Swedish Foundation for Strategic Research, the Royal Swedish Academy of Sciences, the Swedish Diabetes Foundation, the Swedish Society of Medicine, and Novo Nordisk Fonden. Computing was supported by NWO through grant 2018/EW/00408559, BiG Grid, the Dutch e-Science Grid and SURFSARA. All participants signed informed consent and the study was approved by the Medical Ethics Committee of the participating universities.

**The Orkney Complex Disease Study (ORCADES / ORKNEY)**

ORCADES was supported by the Scottish Executive Health Department and the Royal Society. and the European Union framework program 6 EUROSPAN project (contract no. LSHG-CT-2006-018947). DNA extractions were performed at the Wellcome Trust Clinical Research Facility in Edinburgh. We would like to acknowledge the invaluable contributions of Lorraine Anderson, the research nurses in Orkney, and the administrative team in Edinburgh.

**The Prospective Investigation of the Vasculature in Uppsala Seniors Study (PIVUS, REPLICATION_PIVUS)**

Genotyping was performed by the SNP&SEQ Technology Platform in Uppsala (www.genotyping.se). We thank Tomas Axelsson, Ann-Christine Wiman and Caisa Pöntinen for their excellent assistance with genotyping. The SNP Technology Platform is supported by Uppsala University, Uppsala University Hospital and the Swedish Research Council for Infrastructures. This project was supported by grants from the Swedish Research Council, the Swedish Heart-Lung Foundation, the Swedish Foundation for Strategic Research, the Royal Swedish Academy of Sciences, Swedish Diabetes Foundation, Swedish Society of Medicine, and Novo Nordisk Fonden.

**The Prevention of REnal and Vascular ENd-stage Disease Study (PREVEND)**

PREVEND genetics is supported by the Dutch Kidney Foundation (Grant E033), the EU project grant GENECURE (FP-6 LSHM CT 2006 037697), the National Institutes of Health (grant LM010098), The Netherlands organisation for health research and development (NWO VENI grant 916.761.70), and the Dutch Inter University Cardiology Institute Netherlands (ICIN).

**The PROCARDIS study**

PROCARDIS was supported by the European Community Sixth Framework Program (LSHM-CT-2007-037273), AstraZeneca, the Swedish Research Council, the Knut and Alice Wallenberg Foundation, the Swedish Heart-Lung Foundation, the Torsten and Ragnar Soderberg Foundation, the Strategic Cardiovascular Program of Karolinska Institutet and Stockholm County Council, the Foundation for Strategic Research and the Stockholm County Council (560283). HW is supported by the British Heart Foundation Centre for Research Excellence, Wellcome Trust core award (090532/Z/09/Z, 203141/Z/16/Z); AG and HW acknowledge support from the Wellcome Trust (201543/B/16/Z), European Union Seventh Framework Programme FP7/2007-2013 under grant agreement no. HEALTH-F2-2013-601456 (CVGenes@Target) & the TriPartite Immunometabolism Consortium [TrIC]-Novo Nordisk Foundation's Grant number NNF15CC0018486.

**The PROspective study of pravastatin in the elderly at risk Study (PROSPER)**

The research leading to PROSPER results has received funding from the European Union's Seventh Framework Programme (FP7/2007-2013) under grant agreement n° HEALTH-F2-2009-223004. The original PROSPER study was supported by an unrestricted, investigator initiated grant from Bristol-Myers Squibb, USA.

**The PPP Botnia Trio-studien (REPLICATION_BOTNIA.PPP)**

The PPP-Botnia study was supported by grants from the Sigrid Juselius Foundation, the Finnish Diabetes Research Society, the Signe and Ane Gyllenberg Foundation, Swedish Cultural Foundation in Finland, Ollqvist Foundation, Foundation for Life and Health in Finland, Jakobstad Hospital, Medical Society of Finland, Närpes Research Foundation and the Vasa and Närpes Health centers. LG project grants from the Swedish Research Council (Dnr 521-2010-3490, Dnr 521-2010-

3490, Dnr 521-2010-3490, Dnr 521-2007-4037, Dnr 521-2008-2974), the Knut & Alice Wallenberg foundation (KAW 2009.0243), the Torsten och Ragnar Söderbergs Stiftelser (MT33/09), the IngaBritt och Arne Lundberg's Research Foundation (grant nr. 359) and the Heart and Lung Foundation.

**The Rotterdam Study**

The Rotterdam Study is supported by the Erasmus Medical Center and Erasmus University Rotterdam, The Netherlands Organization for Scientific Research (NWO), The Netherlands Organization for Health Research and Development (ZonMw), The Research Institute for Diseases in the Elderly (RIDE), The Netherlands Genomics Initiative, the Ministry of Education, Culture and Science, the Ministry of Health, Welfare and Sports, the European Commission (DG XII), and the Municipality of Rotterdam. The contribution of inhabitants, general practitioners and pharmacists of the Ommoord district to the Rotterdam Study is gratefully acknowledged.

**The National Institute on Aging (NIA) SardiNIA Study**

The authors would like to thank all the volunteers and the major of the four towns involved. This work was supported in part by the Intramural Research Program of the National Institute on Aging (NIA), National Institutes of Health (NIH), and by contract NO1-AG-1-2109, from the NIA, to the SardiNIA ("ProgeNIA") team.

**The SCARFSHEEP study**

The SCARFSHEEP study was supported by the Swedish Heart-Lung Foundation, the Swedish Research Council (projects 8691 and 0593), the Knut and Alice Wallenberg Foundation, the Torsten and Ragnar Söderberg Foundation, the Foundation for Strategic Research, the Stockholm County Council (project 592229) and the Strategic Cardiovascular and Diabetes Programmes of Karolinska Institutet and Stockholm County Council.

**The SORBS Study**

We thank Knut Krohn and Beate Gutsmann for conducting microarray experiments of the Sorbs sample at the Faculty of Medicine of the University of Leipzig. This work was supported by grants from the German Research Foundation (SFB-1052 ''Obesity mechanisms'' A01, B03, SPP 1629 TO 718/2- 1), from the German Diabetes Association, from the DHFD (Diabetes Hilfs- und Forschungsfonds Deutschland) and from IFB Adiposity Diseases (AD2-060E, AD2-06E95, AD2-06E99). IFB Adiposity Diseases is supported by the Federal Ministry of Education and Research (BMBF), Germany, FKZ: 01EO1501.

**The SPLIT Study**

The work is supported by grant ~216-1080315-0302 (to IR) from the Croatian Ministry of Science, Education and Sport; Studies carried out in the Croatian City of Split were supported by Medical Research Council UK. The authors collectively thank a large number of individuals for their help in organizing, planning and carrying out the field work related to the project: Professor Stipan Jankovic and staff at the University of Split Medical School; Dr Branka Salzer from the biochemistry lab "Salzer", Croatia; local general practitioners and nurses; and the employees of several other Croatian institutions who participated in the field work. including but not limited to the University of Rijeka, Croatia; Croatian Institute of Public Health; Institutes of Public Health in Split and Dubrovnik, Croatia. SNP Genotyping of the Split samples was carried out by AROS Applied Biotechnology AS,Aarhus N, Denmark.

**The SUVIMAX Study**

This work was supported by the Institut National de la Santé et de la Recherche Médicale, the Institut National de la Recherche Agronomique, the Université Paris 13, the Centre National de Génotypage and the Commissariat à L'Energie Atomique.

**The Swedish Twins Registry Study (SWEDISHTWINS, REPLICATION_STR)**

This work was supported by grants from the US National Institutes of Health (AG028555, AG08724, AG04563, AG10175, AG08861), the Swedish Research Council, the Swedish Heart-Lung Foundation, the Swedish Foundation for Strategic Research, the Royal Swedish Academy of Science, and ENGAGE (within the European Union Seventh Framework Programme, HEALTH-F4-2007-201413). Genotyping was performed by the SNP&SEQ Technology Platform in Uppsala (www.genotyping.se). We thank Tomas Axelsson, Ann-Christine Wiman and Caisa Pöntinen for their excellent assistance with genotyping. The SNP Technology Platform is supported by Uppsala University, Uppsala University Hospital and the Swedish Research Council for Infrastructures.

**The THISEAS Study (THISEAScases, THISEAScontrols)**

This work was funded by the Wellcome Trust. We thank Cordelia Langford and Sarah Edkins in the genotyping facilities of the Wellcome Trust Sanger Institute. Recruitment for THISEAS was partially funded by a research grant (PENED 2003) from the Greek General Secretary of Research and Technology; we thank all the dieticians and clinicians for their contribution to the project.

**The TWINGENE Study**

This work was supported by grants from the Ministry for Higher Education, the Swedish Research Council (M-2005-1112 and 2009-2298), GenomEUtwin (EU/QLRT-2001-01254; QLG2-CT-2002-01254), NIH grant DK U01-066134, The Swedish Foundation for Strategic Research (SSF; ICA08-0047).

**The TwinsUK Study**

The study was funded by the Wellcome Trust; European Community's Seventh Framework Programme (FP7/2007-2013)/grant agreement HEALTH-F2-2008-201865-GEFOS and (FP7/2007-2013), ENGAGE project grant agreement HEALTH-F4-2007-201413 and the FP-5 GenomEUtwin Project (QLG2-CT-2002-01254). The study also receives support from the Dept. of Health via the National Institute for Health Research (NIHR) comprehensive Biomedical Research Centre award to Guy's & St Thomas' NHS Foundation Trust in partnership with King's College London. TDS is an NIHR senior Investigator. The project also received support from a Biotechnology and Biological Sciences Research Council (BBSRC) project grant. (G20234) .The authors acknowledge the funding and support of the National Eye Institute via an NIH/CIDR genotyping project (PI: Terri Young)".

**The Microisolates in South Tyrol Study (MICROS)**

For the MICROS study, we thank the primary care practitioners Raffaela Stocker, Stefan Waldner, Toni Pizzecco, Josef Plangger, Ugo Marcadent, and the personnel of the Hospital of Silandro (Department of Laboratory Medicine) for their participation and collaboration in the research project. In South Tyrol, the study was supported by the Ministry of Health and Department of Educational Assistance, University and Research of the Autonomous Province of Bolzano, and the South Tyrolean Sparkasse Foundation.

**The Uppsala Longitudinal Study of Adult Men (ULSAM, REPLICATION_ULSAM)**

Genotyping was performed by the SNP&SEQ Technology Platform in Uppsala (www.genotyping.se). We thank Tomas Axelsson, Ann-Christine Wiman and Caisa Pöntinen for their excellent assistance with genotyping. The SNP Technology Platform is supported by Uppsala University, Uppsala University Hospital and the Swedish Research Council for Infrastructures. This project was supported by grants from the Swedish Research Council, the Swedish Heart-Lung Foundation, the Swedish Foundation for Strategic Research, the Royal Swedish Academy of Sciences, Swedish Diabetes Foundation, Swedish Society of Medicine, and Novo Nordisk Fonden.

**The Whitehall Study**

The WHII study has been supported by grants from the Medical Research Council; Economic and Social Research Council; BHF; Health and Safety Executive; Department of Health; National Heart Lung and Blood Institute (HL36310), US, NIH: National Institute on Aging (AG13196), US, NIH; Agency for Health Care Policy Research (HS06516); and the John D and Catherine T MacArthur Foundation Research Networks on Successful Midlife Development and Socioeconomic Status and Health. Genotyping in WHII was supported by BHF grant PG/07/133/24260 and an MRC-GSK pilot programme grant (ID 85374).

**The MolOBB study**

This work was supported by funding from the European Commission to the MolPAGE Consortium (LSHGCT-2004-512066).

**Human islets gene expression**

This work was supported by grants from the Swedish Research Council (including project grants Dnr. 521-2010-2633 to Va.Ly. and 521-2010-3490, strategic research area grant EXODIAB Dnr. 2009-1039, and Linnaeus grant Dnr. 349-2006-237), as well as equipment grants from Wallenberg (KAW 2009-0243) and Lundberg Foundation (grant number 359). L.G. is supported from an Advanced Research Grant from the European Research Council (GENETARGET-T2D, GA 269045) and grants from Pfizer and Va.Ly. from the Novo Nordisk Foundation. Human pancreatic islets were provided by the Nordic Network for Clinical Islet Transplantation by the courtesy of O. Korsgren, Uppsala, Sweden with financial support from EXODIAB and JDRF. Furthermore, this research was supported by Fondazione CARIPARO ("RNA sequencing for quantitative transcriptomics" PhD Program), PRAT 2010 CPDA101217 ("Models of RNA sequencing data variability for quantitative transcriptomics"). We thank Britt-Marie Nilsson and Anna-Maria Veijanovska-Ramsay at Lund University for their technical assistance.

**The ASAP study**

Swedish Research Council [12660], The Swedish Heart-Lung Foundation [201202729].

## SUPPLEMENTARY FIGURES

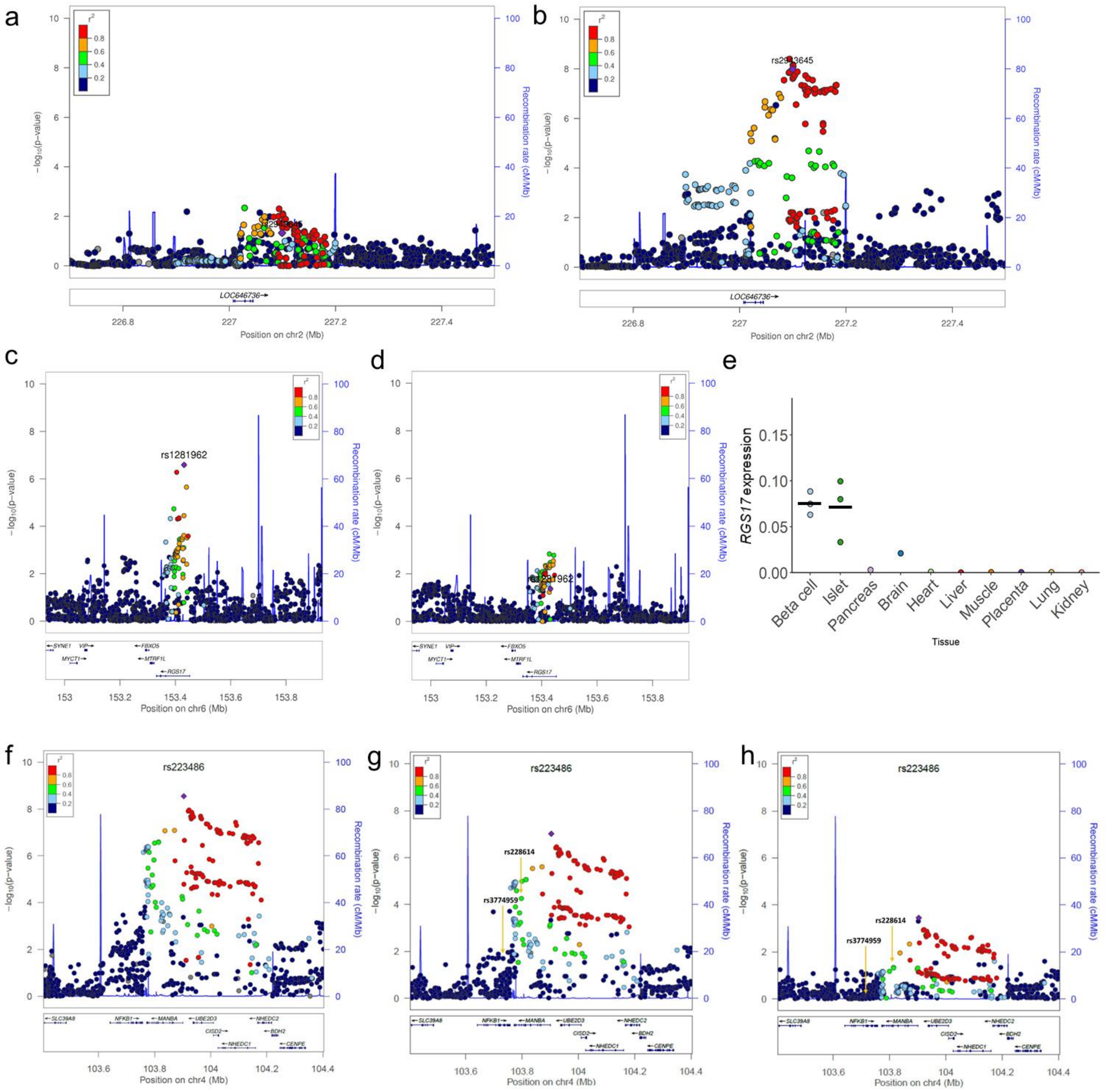


**Supplementary Figure 1. Regional and tissue expression plots.**
**(a)** female-specific and **(b)** male-specific for established *IRS1* locus with sex-dimorphic effects on FI, **(c)** female-specific and **(d)** male-specific on FG for novel *RGS17* locus; e) *RGS17* tissue expression relative to three housekeeping genes (PPIA, B2M and HPRT). For beta cell (n=3) and islets (n=3) data, lines are means. Quantitative RT-PCR was carried out using cDNAs from three human donors (beta-cells and islets). The other tissues were commercial cDNAs (one point observation); Regional plots for **(f)** novel *MANBA/UBE2D3* locus with homogeneous effects between men and women (no conditioning), **(g)** association analysis of *MANBA/UBE2D3* signal conditioned on ulcerative colitis (rs3774959) established variant and **(h)** association analysis of *MANBA/UBE2D3* signal conditioned on multiple sclerosis (rs228614) established variant.

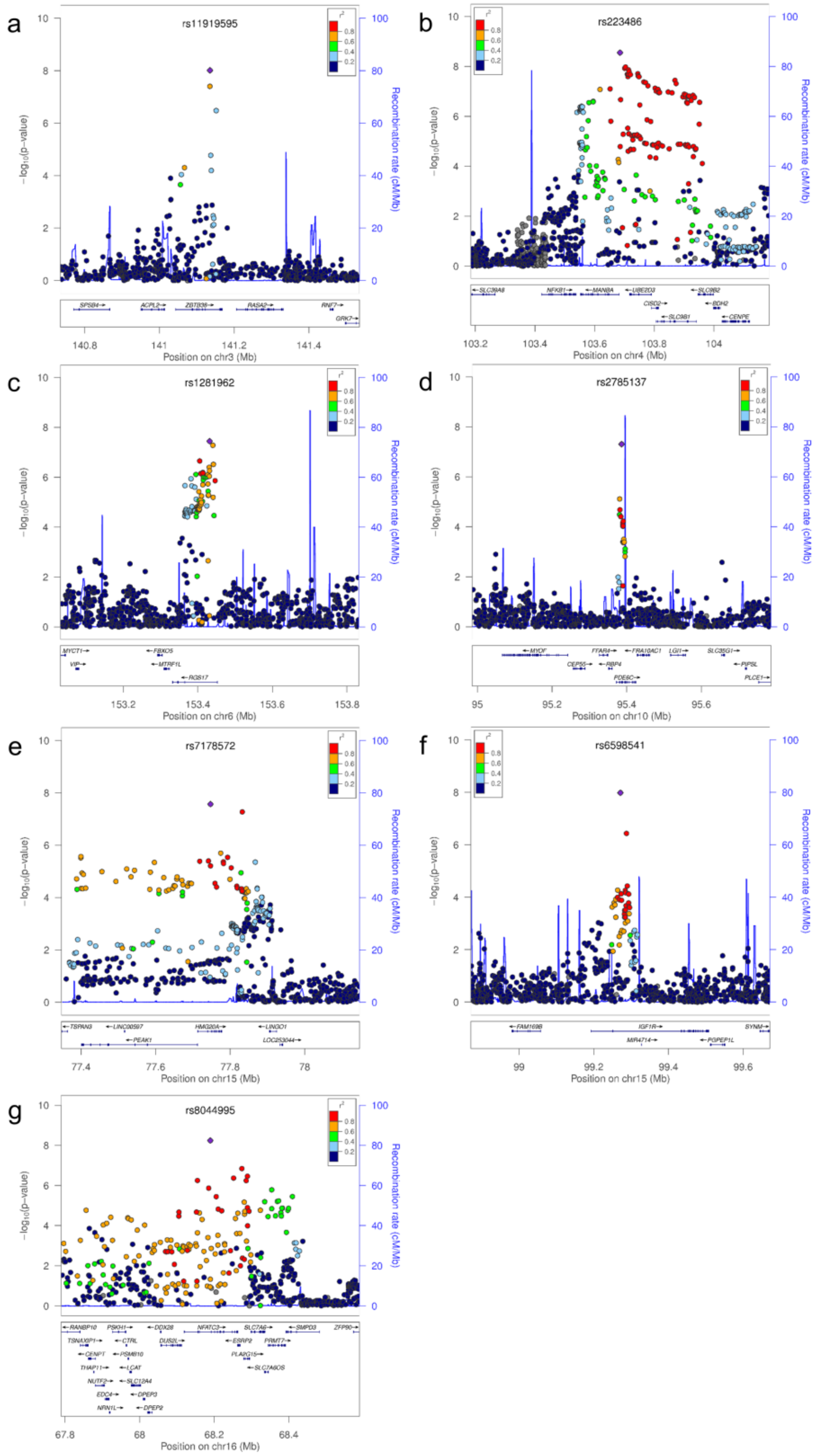

**Supplementary Figure 2. Regional plots for novel loci with sex-combined effects on FG.** **(a)** *ZBTB38*, **(b)** *MANBA/UBE2D3*, **(c)** *RGS17*, **(d)** *PDE6C*, **(e)** *HMG20A*, **(f)** *IGF1R* and (g) *NFATC3*.

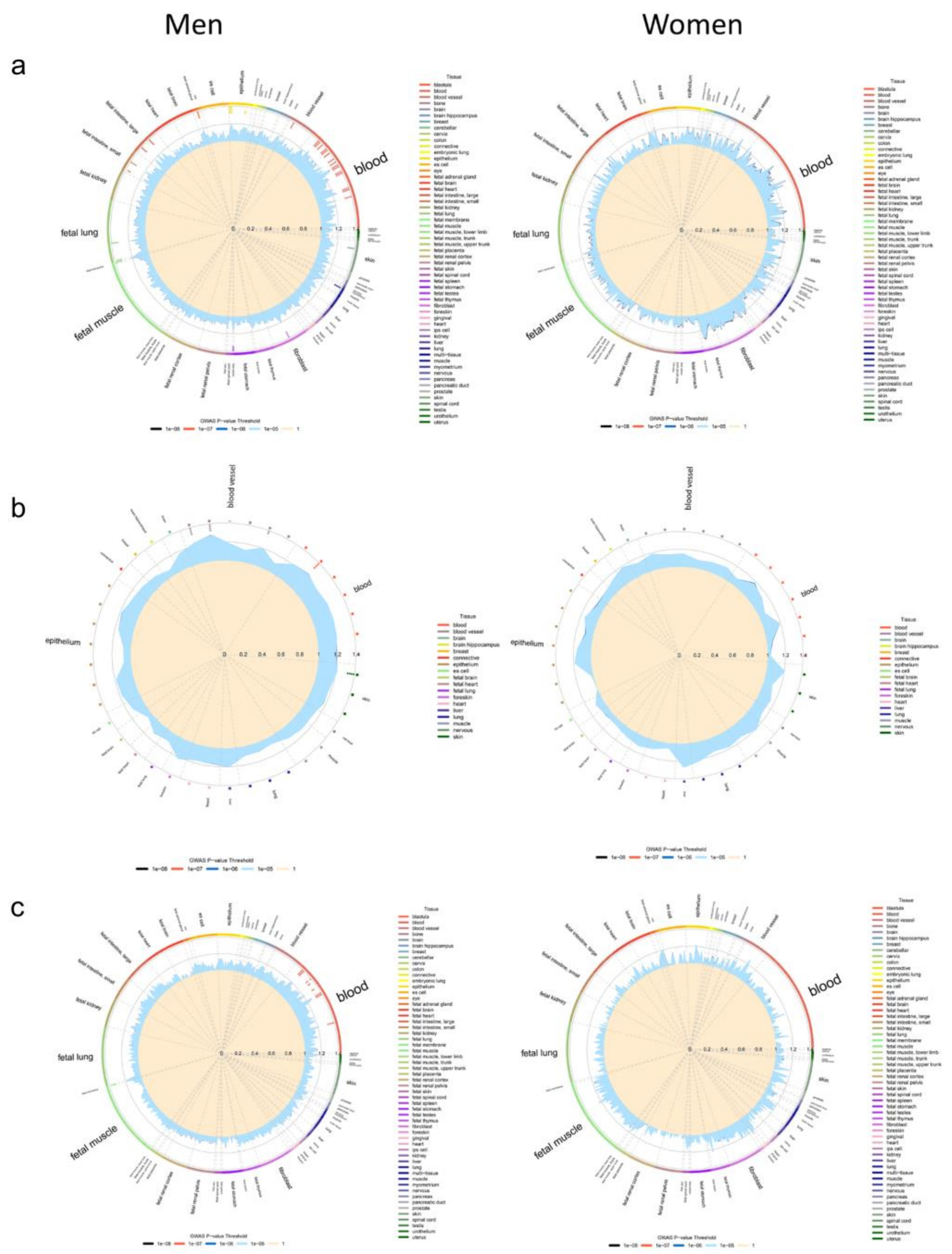


**Supplementary Figure 3**. **GARFIELD enrichment analysis.** Enrichment results for the sex-specific FG meta-analysis summary statistics: **(a)** peaks, **(b)** histone modifications and **(c)** chromatin states.

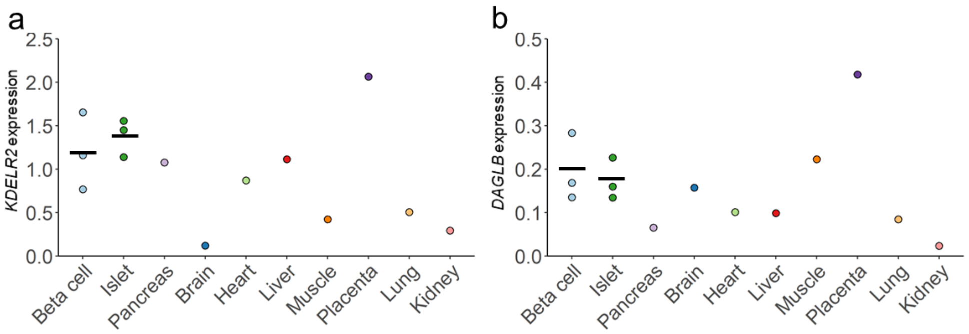


**Supplementary Figure 4. Tissue expression of genes within the novel *ZNF12* locus.**
**(a)** *KDELR2* and **(b)** *DAGLB*. Expression is relative to three housekeeping genes (*PPIA*, *B2M* and *HPRT*). For beta cell (*n*=3) and islets (*n*=3) data, lines are means. Quantitative RT-PCR was carried out using cDNAs from three human donors (beta-cells and islets). The other tissues were commercial cDNAs (one point observation).

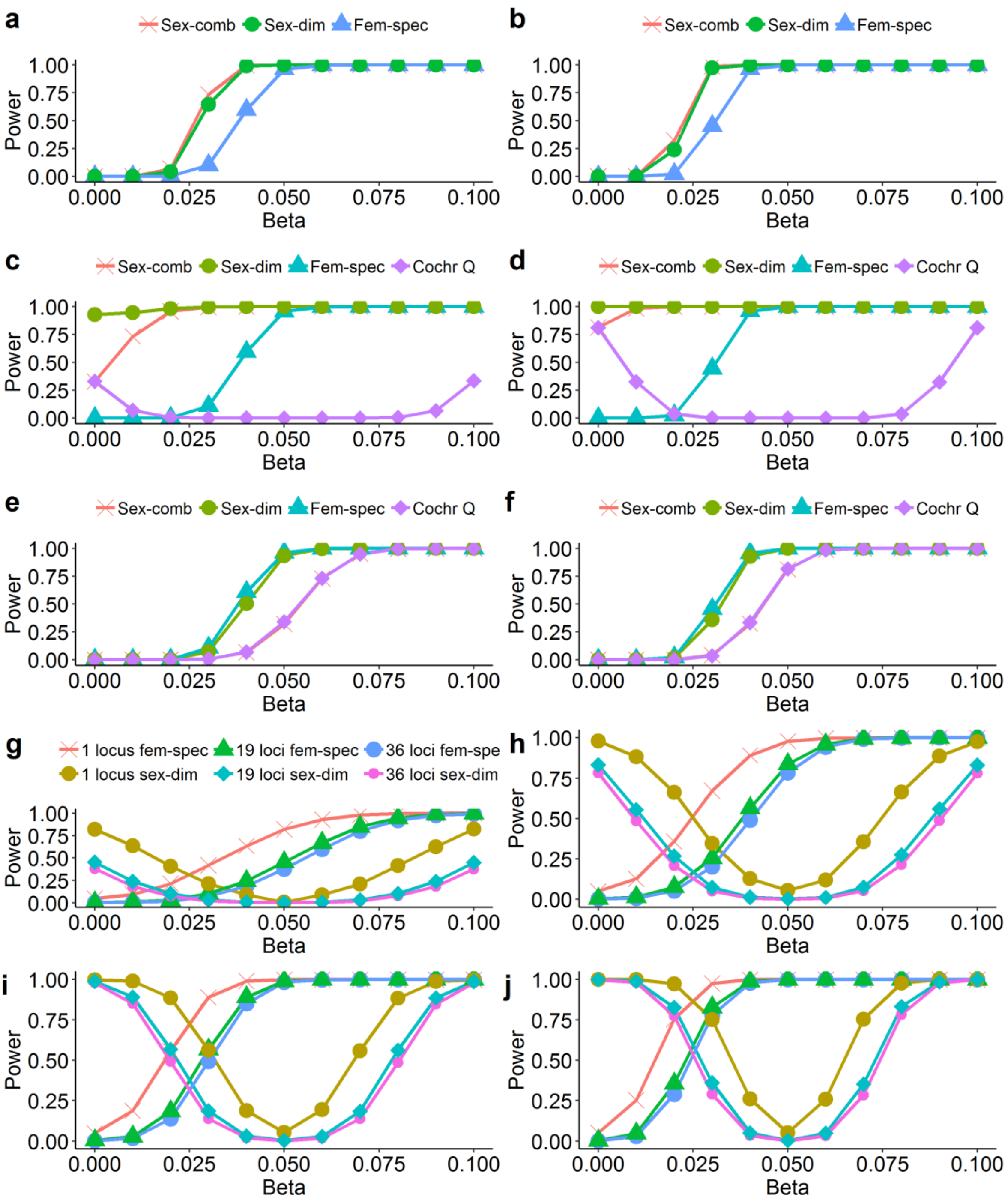


**Supplementary Figure 5. Power of tests for detecting sex heterogeneity through simulations.**

Power of sex-combined, sex-dimorphic and female-specific analyses, as well as Cochran's Q-test to detect associations for evidence of sex heterogeneity under three scenarios of sex-effects: no sex heterogeneity at **(a)** CAF=0.2, and **(b)** CAF=0.5, effects on both sexes with the presence of heterogeneity between them at **(c)** CAF=0.2 and **(d)** CAF=0.5, an effect specific to one sex only, e.g. women at **(e)** CAF=0.2 and **(f)** CAF=0.5. Power of the current sample size to detect sex heterogeneity at established FG (n=36) and FI (n=19) loci using the approach that ignores $P_{\text{sex-dimorphic}}$ and considers only $P_{\text{heterogeneity}}<0.05$ or

$P_{heterogeneity}$ adjusted for multiple testing ($P_{heterogeneity}$<0.05/36 or $P_{heterogeneity}$<0.05/19) under two scenarios of sex-effects: an effect specific to one sex only, e.g. women and effects on both sexes with the presence of heterogeneity between them considering four CAFs: **(g)** CAF=0.05, **(h)** CAF=0.1, **(i)** CAF=0.2, (j) CAF=0.5. The power at $P<5x10^{-8}$ is given for all three tests: sex-combined, sex-dimorphic and female-specific. Colour coding for panels (g-j) is given on panel (g). The power for the heterogeneity test implemented in GWAMA (Cochran's Q-test) is also given. Simulations were based on 70,000 men and 70,000 women. For each parameter setting, 10,000 replicates of data were generated. CAF is the causal variant allele frequency and beta is the effect size in SD units in women. Within each scenario, we considered two CAFs (0.2 and 0.5) and a range of betas (from 0 to 0.1) representing the effect size in SD units in women. For the no sex-heterogeneity setting, the beta in men is the same as in women; for the sex-dimorphic setting, the beta in men is fixed at 0.05 SD units; for the female-specific setting, the beta in men is fixed at zero.
