# Supplementary material for: Sex-dimorphic genetic effects and novel loci for fasting glucose and insulin variability
Source: Nat Commun. 2021 Jan 5;12:24. doi: 10.1038/s41467-020-19366-9 (PMC7785747; doi:10.1038/s41467-020-19366-9)
Supplement: Supplementary file 3 — Reporting Summary [file 41467_2020_19366_MOESM3_ESM.pdf]

# nature research

Corresponding author(s): Prof Inga Prokopenko
Last updated by author(s): Jul 29, 2020

# Reporting Summary

Nature Research wishes to improve the reproducibility of the work that we publish. This form provides structure for consistency and transparency in reporting. For further information on Nature Research policies, see Authors & Referees and the Editorial Policy Checklist .

## Statistics

For all statistical analyses, confirm that the following items are present in the figure legend, table legend, main text, or Methods section.

| n/a | Confirmed | |
|---|---|---|
| ☐ | ☒ | The exact sample size (*n*) for each experimental group/condition, given as a discrete number and unit of measurement |
| ☐ | ☒ | A statement on whether measurements were taken from distinct samples or whether the same sample was measured repeatedly |
| ☐ | ☒ | The statistical test(s) used AND whether they are one- or two-sided<br>*Only common tests should be described solely by name; describe more complex techniques in the Methods section.* |
| ☐ | ☒ | A description of all covariates tested |
| ☐ | ☒ | A description of any assumptions or corrections, such as tests of normality and adjustment for multiple comparisons |
| ☐ | ☒ | A full description of the statistical parameters including central tendency (e.g. means) or other basic estimates (e.g. regression coefficient) AND variation (e.g. standard deviation) or associated estimates of uncertainty (e.g. confidence intervals) |
| ☐ | ☒ | For null hypothesis testing, the test statistic (e.g. *F*, *t*, *r*) with confidence intervals, effect sizes, degrees of freedom and *P* value noted<br>*Give P values as exact values whenever suitable.* |
| ☒ | ☐ | For Bayesian analysis, information on the choice of priors and Markov chain Monte Carlo settings |
| ☒ | ☐ | For hierarchical and complex designs, identification of the appropriate level for tests and full reporting of outcomes |
| ☐ | ☒ | Estimates of effect sizes (e.g. Cohen's *d*, Pearson's *r*), indicating how they were calculated |

*Our web collection on statistics for biologists contains articles on many of the points above.*

## Software and code

Policy information about availability of computer code

| | |
|---|---|
| Data collection | No software was used for data collection. |
| Data analysis | The software used have been described in detail in the Methods section and Supplementary Data 1 with characteristics of participating studies. Software with different versions used by individual GWAS for imputation and association analyses were: MACH, BIMBAM, IMPUTE, BEAGLE, GenABEL/ProbABEL, MACH2QTL, MMAP, MERLIN, QUICKTEST, SNPTEST, PLINK, EMMAX, SPSS, STATA and R. Other software used for subsequent analyses included: SS-Imp v0.5.5, GWAMA v2.2.3, GCTA v1.24.4, TwoSampleMR v0.5.4 (R package), RQ manager v1.2.1, DataAssist v3.0, Affymetrix Power Tools v1.12.0, Illumina Casava v.1.8.2, TopHat v.2.0.2, Bowtie v.0.12.8, Cufflinks tool v.1.3.0, GENCODE v.12, Coding Potential Assessment Tool v1.2.2, Illumina Genome studio v2.0, SHAPEIT v2, Matrix eQTL v2 (R package), GARFIELD v2. |

For manuscripts utilizing custom algorithms or software that are central to the research but not yet described in published literature, software must be made available to editors/reviewers. We strongly encourage code deposition in a community repository (e.g. GitHub). See the Nature Research guidelines for submitting code & software for further information.

## Data

Policy information about availability of data

All manuscripts must include a data availability statement. This statement should provide the following information, where applicable:

- Accession codes, unique identifiers, or web links for publicly available datasets
- A list of figures that have associated raw data
- A description of any restrictions on data availability

GWAS summary statistics for FG/FI analyses presented in this manuscript are deposited on https://www.magicinvestigators.org/downloads/ and will be also be available through the NHGRI-EBI GWAS Catalog https://www.ebi.ac.uk/gwas/downloads/summary-statistics. Summary-level GWAS results for genetic correlation analysis with glycemic traits were downloaded from the LDHub database (http://ldsc.broadinstitute.org/ldhub/). Islets from 89 cadaver donors were provided by the Nordic Islet Transplantation Programme (http://www.medscinet.com/nordicislets/). The dexseq_count python script for RNA sequencing analysis in human pancreatic islets was downloaded from http://www-huber.embl.de/pub/DEXSeq/analysis/scripts/. Raw files for RNA-seq mRNA expression in islet donors have been

deposited in NCBI GEO database with the accession code GSE50398 [https://www.ncbi.nlm.nih.gov/geo/query/acc.cgi?acc=GSE50398].

# Field-specific reporting

Please select the one below that is the best fit for your research. If you are not sure, read the appropriate sections before making your selection.

[x] Life sciences  [ ] Behavioural & social sciences  [ ] Ecological, evolutionary & environmental sciences

For a reference copy of the document with all sections, see nature.com/documents/nr-reporting-summary-flat.pdf

# Life sciences study design

All studies must disclose on these points even when the disclosure is negative.

| | |
|---|---|
| Sample size | No sample size calculation was made. We aimed to bring together the largest possible sample size with the following collection of samples: 38 GWAS, including up to 80,512 individuals genotyped using either Illumina or Affymetrix genome-wide SNP arrays; ii) 27 studies with up to 47,150 individuals genotyped using the iSELECT Metabochip array (~197K SNPs) designed to support efficient large-scale follow-up of putative associations for glycemic and other metabolic and cardiovascular traits; iii) 8 studies, including up to 21,173 individuals genotyped for custom variant sets; and iv) 4 studies, including up to 13,613 individuals from four family-based studies. We obtained FG/FI sex-specific results for up to 73,089/50,404 women and 67,506/47,806 men and from population-based studies; sex-combined meta-analyses for these traits additionally included 13,613 individuals from four family-based studies. |
| Data exclusions | Individuals were excluded from the analysis if they had a physician diagnosis of diabetes, were on diabetes treatment (oral or insulin), or had a fasting plasma glucose equal to or greater than 7 mmol/L. Individual studies applied further sample exclusions, including pregnancy, non-fasting individuals and type 1 diabetes. Individuals from case-control studies were excluded if they had hospitalization or blood transfusion in the 2-3 months before phenotyping took place. For each study, samples reflecting duplicates, low call rate, gender mismatch, or population outliers were excluded. Low-quality SNPs were excluded by the following criteria: call rate <0.95, minor allele frequency <0.01, minor allele count <10, Hardy-Weinberg P-value <10−4, or imputation quality score <0.5. Detailed descriptions of study-specific glycemic measurements are given in Supplementary Data 1 with characteristics of participating studies. SNPs imputed to the 1000 Genomes reference panel with imputation quality score <0.7 were excluded after imputation. |
| Replication | This was an observational study - analyses were based on all available data. |
| Randomization | Not relevant because the study is not experimental. |
| Blinding | Not relevant because the study is not experimental. |

# Reporting for specific materials, systems and methods

We require information from authors about some types of materials, experimental systems and methods used in many studies. Here, indicate whether each material, system or method listed is relevant to your study. If you are not sure if a list item applies to your research, read the appropriate section before selecting a response.

## Materials & experimental systems

| n/a | Involved in the study | |
|---|---|---|
| [x] | [ ] | Antibodies |
| [x] | [ ] | Eukaryotic cell lines |
| [x] | [ ] | Palaeontology |
| [x] | [ ] | Animals and other organisms |
| [ ] | [x] | Human research participants |
| [x] | [ ] | Clinical data |

## Methods

| n/a | Involved in the study | |
|---|---|---|
| [x] | [ ] | ChIP-seq |
| [x] | [ ] | Flow cytometry |
| [x] | [ ] | MRI-based neuroimaging |

## Human research participants

Policy information about studies involving human research participants

| | |
|---|---|
| Population characteristics | Analyses were conducted on non-diabetic individuals of European ancestry (men, age range:11.54-76.65; women, age range:11.53-76.29). At study-level, the association between each variant and untransformed fasting glucose or natural logarithm transformed fasting insulin was adjusted by age, sex, and study-specific parameters with or without body-mass index. |
| Recruitment | The majority of studies are population-based cohorts, case-control or family-based studies with related individuals. Subjects included are men and women of European ancestry with no diagnosed diabetes. |
| Ethics oversight | All participating studies were approved by their appropriate ethics review board and all subjects provided informed consent. |

Note that full information on the approval of the study protocol must also be provided in the manuscript.
