# Supplementary material for: Sex-dimorphic genetic effects and novel loci for fasting glucose and insulin variability
Source: Nat Commun. 2021 Jan 5;12:24. doi: 10.1038/s41467-020-19366-9 (PMC7785747; doi:10.1038/s41467-020-19366-9)
Supplement: Supplementary file 4 — Description of Additional Supplementary Files [file 41467_2020_19366_MOESM4_ESM.pdf]

Description of Additional Supplementary Files

Supplementary Data 1
Study design, sample size, and data quality control, phenotype descriptive statistics, genotyping methods, quality control of SNPs, imputation, and statistical analysis for the contributing studies.

Supplementary Data 2
Sex-combined, sex-specific and sex-dimorphic meta-analysis results for 36 established FG variants. The -log10 P values obtained from Cochran's Q-test are also shown.

Supplementary Data 3
Sex-combined, sex-specific and sex-dimorphic meta-analysis results for 19 established FI variants. The -log10 P values obtained from Cochran's Q-test are also shown.

Supplementary Data 4
Look-up of novel SNPs (lead or proxy) from sex-combined meta-analysis in a previous MAGIC meta-analysis.

Supplementary Data 5
Genetic correlations between FI and 201 traits available in the LDhub.

Supplementary Data 6
Genetic correlations between FG and 201 traits available in the LDhub.

Supplementary Data 7
SNPs used for the WHR instrument in the Mendelian Randomization analysis of WHR-FI relationship in women and men.

Supplementary Data 8
Results of the bi-directional, sex-specific MR analyses.

Supplementary Data 9
Whole blood RNA expression data for known and novel genes with evidence of sex heterogeneity.

Supplementary Data 10A
Variants at FG/FI loci used to investigate on sex-specific/-dimorphic gene expression in a range of tissues.

Supplementary Data 10B
Effects at selected FG/FI loci on sex-dimorphic gene expression in RNA expression in whole blood from 3,621 Netherlands Twin Register (NTR)/ Netherlands Study of Anxiety and Depression (NESDA) study individuals with the Affymetrix U219 array data.

Supplementary Data 10C
Effects at FG/FI loci on sex-dimorphic gene expression in gluteal and abdominal fat from MolOBB study.

Supplementary Data 10D
Effects at FG/FI loci on gene expression in lymphoblastoid cell lines (LCL) from HapMap 2 individuals.

Supplementary Data 10E
Effects at FG/FI loci on gene expression in liver, heart, aorta adventitia/intima media and mammary artery intima-media from the Advanced Study of Aortic Pathology (ASAP) dataset.

Supplementary Data 10F
Effects at FG/FI loci on gene expression ($P<0.05$) in islets of individuals with IGT compared to those with normal glucose tolerance.

Supplementary Data 10G

Effects at FG/FI loci on gene expression in fat, LCLs, and skin tissues from women analysed on MuTHER consortium dataset.

.
