# Supplementary material for: Sex-dimorphic genetic effects and novel loci for fasting glucose and insulin variability
Source: Nat Commun. 2021 Jan 5;12:24. doi: 10.1038/s41467-020-19366-9 (PMC7785747; doi:10.1038/s41467-020-19366-9)
Supplement: Supplementary file 2 — Peer Review File [file 41467_2020_19366_MOESM2_ESM.pdf]

Reviewers' comments:

Reviewer #1 (Remarks to the Author):

Lagou et al. report on the sex-dimorphic and sex-combined fasting glucose (FG) and fasting insulin (FI) genetic effects from genome-wide association studies of European descent individuals without diabetes. This is a comprehensive manuscript on investigating the sex-dimorphic effects of FI and FG. The authors found only two sexually dimorphic effects on FI, suggesting limited sex-dimorphic effects on FG and FI.

Specific comments:
1. The abstract highlights two sex-heterogeneous effect on FI and 7 sex-homogeneous effects on FG, but it is unclear why those particular results are highlights. The authors should also take care in making sure they specify the reference group when performing comparisons.
2. Are there differences in heritability in men and women for FI and FG?
3. There are many characteristics that differ among the contributing studies. Could the sex-specific or sex-dimorphic effects be due to differences in the contributing studies?
4. Since a two-df test was used for the sex-dimorphic analysis, this test can also pick up overall effects of the genotypes, so the authors require nominal sex heterogeneity (p.het <0.05). Why is a nominal threshold sufficient here?
5. To test the heterogeneity of allelic effects, the same variance of effect sizes were assumed in men and women. Is this what is observed? Is it a reasonable assumption here?
6. Page 8, lines 340-341 say that lead variants that were significant at 5x10-8 in the sex-dimorphic meta-analysis were considered for subsequent evaluation, but then the authors go on to report on the nominal evidence for heterogeneity at rs10195252 that doesn't meet their specified sex-dimorphic nor heterogeneity p-value thresholds. If the authors are not finding results that meet their pre-specified thresholds, this should first be stated and then authors can report the marginal results of interest. The authors should also limit the use of the word "significant" throughout the manuscript.

Minor
1. Where all the studies of self-reported European ancestry or was this based on genetic ancestry?
2. pg 20, line 708 – what is the p.het-ty? Is this a typo or something else?
3. Pg 9, line 346 – wrong table is referenced
4. Why are the p-value thresholds for heterogeneity in the methods (pg 20 lines 707-708) different from the thresholds in the results (pg 9, line 343)?
5. In the selection of WHR SNPs for the bidirectional two-sample Mendelian Randomization, why was one SNP missing in men? Was a proxy SNP available instead?
6. Methods for the approximate conditional analyses (referred to on pg 10-11, lines 405-406), seems to be missing from the Online methods.
7. Figure 4 – different symbols or lines should be used to distinguish the three tests.

Reviewer #2 (Remarks to the Author):

Epidemiologic studies have revealed men are more insulin resistant and have higher fasting glucose levels than women, but women are more likely to have elevated 2-hr glucose levels (i.e., IGT). The goal of this study is to evaluate if genetic basis for these differences by evaluating sex differences in GWA results from the MAGIC Consortium for fasting glucose and fasting insulin. A strength of this study is its systematic evaluation of sexual dimorphism in genetic effects on such a large data set (151,151 European ancestry individuals),

The authors test for sexually dimorphic effects in 2 ways, first by performing sex-specific analyses and comparing effect sizes between men and women, and second by performing a sex-combined analysis

that includes a SNP*sex interaction term. The novel findings reported from this study are: evidence for sexual dimorphism for FI at one established locus (IRS1) and one novel locus (ZNF12) and evidence for significant association with FG in sex-combined analysis at 6 novel loci. Statistical evidence for the loci exhibiting loci is not strong: The IRS1 association modestly surpasses Bonferroni-corrected significance thresholds on the sex-stratified analysis, but the ZNF12 locus does not (Phet=0.005). There are several issues that should be addressed:

1. The way the 2 sets of analyses are presented is confusing to me. The authors state that the interaction analysis has more power than the sex-specific analyses, and this is certainly consistent with the results shown for the analyses of established FI/FG loci, where the lead SNPs at 27 FG and 9 FI loci reached genome-wide significance, yet only one of these reached Bonferroni-corrected significance in the sex-stratified analysis. For these established FI/FG loci, the authors give precedence to the results from the (more conservative) sex-stratified analysis, highlighting that they detected evidence for sexual dimorphism at only one of the established FG/FI loci. Yet for detection of novel loci, the requirement for statistically significant detection of sexual dimorphism is genome-wide significance in the (more liberal) SNP*sex analysis and only nominal (i.e., uncorrected for multiple comparisons) significance in sex-stratified analysis.

2. Figure 4 c,d shows nicely that power is somewhat similar between Cochran's Q test and the 2-degree of freedom interaction test. How do the false positive rates compare? Related to this, it would be useful to provide the lambdas (GCs) for these 2 tests based on the GWAS.

3. For previously established FG/FI loci, was the lead SNP analyzed taken the same as the published lead SNP based on a sex-combined analysis, or did you pull out from this region the SNPs showing the greatest evidence for sexual dimorphic effects?

4. Please provide a short description of Cochran's Q-test for heterogeneity.

5. In the analyses of established FI/FG loci: No loci showed significance evidence for heterogeneity in allelic effects on FG, and only 1 locus showing significant evidence for heterogeneity in allelic effects on FI. The FI-associated locus was significant at p=0.005, only slightly under the Bonferroni corrected threshold of p 0.002. This variant was significant in men only; but of interest, the variant in IRS1, a locus where sex-specific effects have been previously reported for % body fat and lipids. Is this the same SNP? In some regards seems like the same result being reported, albeit with a highly correlated trait.

6. Page 8, line 323: What is the rationale for obtaining only the sex-combined analyses from the 4 family-based studies? That is, couldn't sex-specific and sex-dimorphic analyses be obtained from these family designs also (e.g., as for the Amish)? As written, the implication is that it was because they were family studies, but perhaps the reason is these were the only results available?

7. Minor:

8. Page 7, line 280-281: replace "…whereas more women than men have elevated…" with "…whereas women [are more likely] than men to have elevated…"

Reviewer #3 (Remarks to the Author):

The authors have performed a genome-wide association study of up to 73,089 men and 67,506 women, to examine sex-dimorphism and sex-combined genetic effects on fasting glucose (FG) and

fasting insulin (FI). They identify sex-heterogeneity in the effect of the previously identified IRS1 locus on FI, and identify a novel sex-dimorphic FI locus at ZNF12. Seven novel loci are reported for FG, none of which show sex-dimorphic effects. A causal effect of waist-hip ratio on FI in women but not in men is shown using a mendelian randomization analysis. Overall, the manuscript is well-written and includes several novel findings. I have the following remarks:

1. Lines 337-357: To study sex-dimorphic effects for the already established FG/FI loci, the authors use a two-step approach where they first, among all established FG/FI loci, identify the ones that show genome-wide significance in the 2 df joint model of the SNP main effect and interaction with sex, and in the second step, examine the 1 df SNP*sex interaction term with Bonferroni correction for the number of loci taken forward from the first step. I do not see this approach fully justified, in particular as the SNP*sex interaction term is part of the 2df model that is studied in the first step, and thus the second step in this approach is not completely independent from the first step. Thus, instead of looking at the 2df model at all, I suggest a simpler one-step approach where the authors examine the significance of the SNP*sex interaction term for all the previously known 36 FG and 19 FI loci, and run Bonferroni correction for the number of independent loci tested.

2. Lines 412-424: In the genetic correlation analyses, it seems that the sex-specific results for FG and FI were correlated with sex-combined results for other GWAS traits, which makes the interpretation of the results tricky. It would be worthwhile to run additional analyses for the GWAS traits where sex-specific results are available (e.g. WHR, BMI), to study correlations between sex-specific results for FG and FI, and sex-specific results for the other GWAS traits.

3. Lines 426-439: Was there evidence of horizontal pleiotropy in the WHR vs. FI mendelian randomization analysis, looking at the intercept from MR-Egger? If there was, it would be worthwhile to perform random effect analyses as well, and perhaps also MR-PRESSO analysis, to correct for horizontal pleiotropy.

4. Lines 426-439: The weakness of using WHRadjBMI is that it does not allow to tell whether the causal effect on FI is driven by waist circumference (abdominal fat) or hip circumference (gluteofemoral fat), which is highly relevant when talking about sexually dimorphic effects. Thus, it would be greatly helpful to see additional analyses for waist circumference adjusted for BMI and hip circumference adjusted for BMI, to tell which fat depot drives the causal relationship with FI in men and women.

5. Lines 487-488, 577-579, and 588-589: It is true that beta cells are highly relevant to glucose metabolism, but I find it surprising that a locus associated with insulin resistance in non-diabetic individuals is linked to beta-cells. How would the authors interpret this intriguing finding?

6. Figure 3: As leptin is produced directly in proportion to total body fat mass, leptin levels and BMI are generally very strongly correlated. The genetic correlation analysis was performed for leptin not adjusted BMI. A more interesting question is, was there a correlation between FI and leptin levels adjusted for BMI?

We much appreciate the time and effort of the reviewers, as well as the consideration of our research and findings by Nature Communications. The reviewers evaluated our report as a comprehensive dissection of sex-dimorphic genetic effects on fasting glycaemic traits in individuals without diabetes, a well-written manuscript reporting novel findings. We have put our major effort into carefully addressing the comments from all three reviewers, which we found useful and constructive. We performed specific additional analyses and revised sustantially the manuscript according to the suggestions from the reviewers. This effort brought some confirmatory and additional crisp observations. We are thankful to the reviewers for their suggestions.

Reviewers' comments:

Reviewer #1 (Remarks to the Author):

Lagou et al. report on the sex-dimorphic and sex-combined fasting glucose (FG) and fasting insulin (FI) genetic effects from genome-wide association studies of European descent individuals without diabetes. This is a comprehensive manuscript on investigating the sex-dimorphic effects of FI and FG. The authors found only two sexually dimorphic effects on FI, suggesting limited sex-dimorphic effects on FG and FI.

Specific comments:

1. The abstract highlights two sex-heterogeneous effect on FI and 7 sex-homogeneous effects on FG, but it is unclear why those particular results are highlights. The authors should also take care in making sure they specify the reference group when performing comparisons.

> We are thankful to the Reviewer for this observation. Thus far, our study is the largest study examining genetic variability effects on the levels of glucose and insulin in fasting state. This discovery effort is expanding our knowledge about genomic loci for these traits compared to the latest study published yet in 2012 led by the same group of investigators within the MAGIC. To our belief, for GWAS efforts, it is important to highlight the specific genetic loci discovered within them and therefore, bring biological and new pathophysiological understanding to the traits we studied. As we state in the aims of this manuscript and summarise similarly in the abstract, we undertook several strains of investigation, including sex-combined and sex-dimorphic effects dissection on fasting glycaemic traits, and the loci highlighted are the new discoveries from our study.
> We also agree with the Reviewer about the need to specify the reference group for all comparisons, and this is now included in the abstract, copied here below:

"We identified sex dimorphism in allelic effects on FI at IRS1 and ZNF12 loci, the latter showing higher RNA expression in whole blood in women compared to men. We also observed sex-homogeneous effects on FG at seven novel loci. FI in women showed stronger genetic correlations than in men with waist-to-hip ratio and anorexia nervosa."

2. Are there differences in heritability in men and women for FI and FG?

> To our knowledge, only one study to date (van Dongen et al., J Lipid Res 2013) has reported heritability estimates for FG and FI by age and sex in a large population-based sample of twin families, including young and older adults. In this paper, significant qualitative sex differences were observed only for glucose in the older adults where the narrow-sense heritability (i.e. due to additive effects) was found lower in men than women at this age with a larger environmental variance. However, no differences in heritability estimates by sex were observed for insulin.

3. There are many characteristics that differ among the contributing studies. Could the sex-specific or sex-dimorphic effects be due to differences in the contributing studies?

We provide Supplementary Table 1 with descriptive information by gender and for additional traits related to FG and FI, such as age and BMI. In previous meta-analyses within the MAGIC consortium with several studies also included in the present analysis, some heterogeneity was detectable but this was not affecting the association between traits and common variants, which is also the focus of the current study. Furthermore, for each lead SNP variant, we added to the **Table 1** reporting novel loci and **Supplementary Tables 2 and 3** reporting established loci, the estimates of heterogeneity index I^2 (Higgins et al., BMJ 2003), which describes the percentage of total variation across studies that is due to heterogeneity rather than chance. No heterogeneity across studies was observed for the novel loci. Among established FG/FI loci, some heterogeneity was observed for some of the loci with predominantly the largest effects on the respective trait variability. For example, nominal heterogeneity in the effects on FG was observed for *G6PC2, SLC30A8, MTNR1B* and *FAM148B/VPS13C/C2CD4A/B* and in the effects on FI for *YSK4* and *TCF7L2*. This result is not surprising since such loci exhibit relatively strong effects for common variants in large meta-analyses, while in individual studies, such effects might somehow fluctuate, since many contributing studies have sample sizes of a couple of thousands and can still reflect sampling fluctuations compared to the real populations they have been drawn from. The nominal heterogeneity in the estimates of sex-dimorphic effects repeats the same trend of the sex-combined effects in both, FG and FI.

4. Since a two-df test was used for the sex-dimorphic analysis, this test can also pick up overall effects of the genotypes, so the authors require nominal sex heterogeneity (p.het <0.05). Why is a nominal threshold sufficient here?

We clarified our presentation of results regarding statistical significance throughout the manuscript. For clarity and consistence, we removed the notation of nominal sex-heterogeneity. The corrected text on p. 9, lines 360-363 now read as follows:

"To identify novel loci based on modelling heterogeneity and through sex-combined analyses, we required that the lead SNP was genome-wide significant in the 2df sex-dimorphic or in the 1df sex-combined test of association ($P\leq5\times10^{-8}$)."

5. To test the heterogeneity of allelic effects, the same variance of effect sizes were assumed in men and women. Is this what is observed? Is it a reasonable assumption here?

We used two tests to assess the evidence of heterogeneity in effects between women and men. Our primary test was Cochran's Q statistic, which does not assume the same variance in effect sizes between the two sexes. The second test was a t-test, which does assume equal variances in effect sizes. We have dropped the t-test from our results.

6. Page 8, lines 340-341 say that lead variants that were significant at 5x10-8 in the sex-dimorphic meta-analysis were considered for subsequent evaluation, but then the authors go on to report on the nominal evidence for heterogeneity at rs10195252 that doesn't meet their specified sex-dimorphic nor heterogeneity p-value thresholds. If the authors are not finding results that meet their pre-specified thresholds, this should first be stated and then authors can report the marginal results of interest. The authors should also limit the use of the word "significant" throughout the manuscript.

We acknowledge that the reporting was confusing and unified our definitions for statistical significance (see also our response for point 4). We evaluated all established FG and FI for evidence of sex-dimorphic effects. We applied Bonferroni correction for the 36/19 FG/FI loci, and although none of the variants reached the Bonferroni corrected significance levels, we comment on the results with suggestive evidence for sex heterogeneity. We also limited the usage of word "significant" throughout the text. The revised paragraph on p. 8-9 now reads as follows:

"Sex-dimorphic effects at established FG/FI loci
To define the extent of sex-dimorphic effects, we evaluated sex heterogeneity at 36/19 established FG/FI loci[6] (**Suppl. Tables 2/3**). Although not reaching the statistical significance after Bonferroni correction for multiple testing ($P_{heterogeneity}$≤0.0014 for FG with 36 variants and $P_{heterogeneity}$≤0.0026 for FI with 19 variants), we observed suggestive evidence for heterogeneity at *IRS1,* where variant rs2943645 was associated with FI in men only (β_{male}=0.022, $P_{male}=1.0\times10^{-8}$, $P_{sex\text{-}dimorphic}=1.0\times10^{-8}$) with differences in allelic effects by sex ($\Delta\beta_{(\beta male-\beta female)}$=0.015, $P_{heterogeneity}$=0.0053) (**Suppl. Table 3**, **Suppl. Figure 1a/b**). The male-specific effects on FI variability were consistent with previously reported effects specific to men on percentage of body fat and lipids at the *IRS1* locus[10]. Additionally, we observed nominal evidence for heterogeneity at *COBLL1*/*GRB14* (rs10195252, $P_{heterogeneity}$=0.039) with more pronounced effects on FI in women (β_{female}=0.018, $P_{female}=1.2\times10^{-6}$, $P_{sex\text{-}dimorphic}=1.5\times10^{-6}$) than men ($\beta_{male}$=0.007, P_{male}=0.073) (**Suppl. Table 2**). Our observations were consistent with previous reports of effects at *COBLL1*/*GRB14* specific to women on waist-to-hip ratio (WHR) and triglycerides. Four established FG loci, *PROX1*, *ADCY5*, *PCSK1* and *SLC30A8*, showed larger effects in women with nominal evidence for sex heterogeneity (**Suppl. Table 3**). We did not observe association at the previously reported female-specific FG locus *COL26A1* (*EMID2*) (rs6961305, r^2_{EUR}=0.89 with reported SNP rs6947345, $P_{sex\text{-}combined}$=0.199, $P_{sex\text{-}dimorphic}$=0.035).

Minor

1. Where all the studies of self-reported European ancestry or was this based on genetic ancestry?

Examining population stratification is a standard procedure during GWAS quality control. Participating studies have used multidimensional scaling and clustering to identify ethnic outliers. Such analyses for defining ethnic outliers and non-European individuals, were performed with inclusion of HapMap or 1000 Genome project reference samples, which specifically include individuals of European descent.

2. pg 20, line 708 – what is the p.het-ty? Is this a typo or something else?

This is a typo and has been corrected.

3. Pg 9, line 346 – wrong table is referenced

The referenced table has been corrected.

4. Why are the p-value thresholds for heterogeneity in the methods (pg 20 lines 707-708) different from the thresholds in the results (pg 9, line 343)?

We thank the Reviewer for noticing this. The *P*-value thresholds in the Methods are indeed those that use correction for multiple testing for all established FG and FI variants. Since in the revised version of manuscript we evaluate all established signals (36/19 for FG/FI), regardless of whether the lead SNP variants at the established FG and FI loci reached genome-wide significance in the sex-dimorphic meta-analysis (see main point 4), the thresholds reported in

the Methods are the correct ones and now correspond to the ones reported in the revised Results.

5. In the selection of WHR SNPs for the bidirectional two-sample Mendelian Randomization, why was one SNP missing in men? Was a proxy SNP available instead?

This is a typo and has been corrected in the manuscript.

6. Methods for the approximate conditional analyses (referred to on pg 10-11, lines 405-406), seems to be missing from the Online methods.

We thank the Reviewer for their careful observation and apologise for missing text. We have added to the main text the respective phrase and expanded the methods to include the description of approximate conditional analysis as below:

*"**Approximate conditional analysis***
We performed approximate conditional analysis by using the Genome-Wide Complex Trait Analysis (GCTA) tool to assess whether the signals within the MANBA/UBE2D3 genomic region associated with FG represented independent associations or the same shared signal with multiple sclerosis and ulcerative colitis. GCTA implements an approximate conditional analysis of phenotype associations using GWAS summary statistics while incorporating LD information from a reference sample. Here, we used individual level genotype data from the PIVUS study (European ancestry) as the LD reference. The GCTA approach allows the estimation of an adjusted effect size estimate with a corresponding p-value for the association of a variant with a phenotype, corrected for the effect of another adjacent SNP or a group of SNPs, based on the extent of LD between them."

7. Figure 4 – different symbols or lines should be used to distinguish the three tests.

Figure 4 has been modified as the Reviewer suggested.

Reviewer #2 (Remarks to the Author):

Epidemiologic studies have revealed men are more insulin resistant and have higher fasting glucose levels than women, but women are more likely to have elevated 2-hr glucose levels (i.e., IGT). The goal of this study is to evaluate if genetic basis for these differences by evaluating sex differences in GWA results from the MAGIC Consortium for fasting glucose and fasting insulin. A strength of this study is its systematic evaluation of sexual dimorphism in genetic effects on such a large data set (151,151 European ancestry individuals).

The authors test for sexually dimorphic effects in 2 ways, first by performing sex-specific analyses and comparing effect sizes between men and women, and second by performing a sex-combined analysis that includes a SNP*sex interaction term. The novel findings reported from this study are: evidence for sexual dimorphism for FI at one established locus (IRS1) and one novel locus (ZNF12) and evidence for significant association with FG in sex-combined analysis at 6 novel loci. Statistical evidence for the loci exhibiting loci is not strong: The IRS1 association modestly surpasses Bonferroni-corrected significance thresholds on the sex-stratified analysis, but the ZNF12 locus does not (Phet=0.005). There are several issues that should be addressed:

1. The way the 2 sets of analyses are presented is confusing to me. The authors state that the interaction analysis has more power than the sex-specific analyses, and this is certainly consistent with the results shown for the analyses of established FI/FG loci, where the lead SNPs at 27 FG and

9 FI loci reached genome-wide significance, yet only one of these reached Bonferroni-corrected significance in the sex-stratified analysis. For these established FI/FG loci, the authors give precedence to the results from the (more conservative) sex-stratified analysis, highlighting that they detected evidence for sexual dimorphism at only one of the established FG/FI loci. Yet for detection of novel loci, the requirement for statistically significant detection of sexual dimorphism is genome-wide significance in the (more liberal) SNP*sex analysis and only nominal (i.e., uncorrected for multiple comparisons) significance in sex-stratified analysis.

The Reviewer is not entirely correct about the power of the different approaches. The interaction analysis does not have more power than the sex-specific analyses; rather it is the sex-dimorphic (2df) test that has more power (Magi et al., Genet Epidemiol 2010). Nevertheless, we agree with the Reviewer that the presentation of results for established loci was somewhat confusing, and we have revised our reporting of these findings. Please see our responses to Reviewer #1, points 4 and 6.

2. Figure 4 c,d shows nicely that power is somewhat similar between Cochran's Q test and the 2-degree of freedom interaction test. How do the false positive rates compare? Related to this, it would be useful to provide the lambdas (GCs) for these 2 tests based on the GWAS.

We are confused by the comment of the Reviewer since the power of Cochran's Q test and the 2df association test are not similar. The two tests are actually testing different null hypotheses: Cochran's Q test is testing the null of no heterogeneity in effects between women and men, whilst the 2df association test is testing the null of no association with the trait, whilst allowing for heterogeneity in effects between men and women. Consequently it is not appropriate to compare the false positive error rates. We have now added to the Statistical analysis section of the Methods GCs values for the 2 tests on the GWAS:

"The lambda values for FG and FI sex-dimorphic and Cochran's Q test were as follows: FG ($\lambda_{sex\text{-}dimorphic\_test}$=1.06, $\lambda_{CochransQ\_test}$=1.01), FI ($\lambda_{sex\text{-}dimorphic\_test}$= 1.06, $\lambda_{CochransQ\_test}$=1.00)."

3. For previously established FG/FI loci, was the lead SNP analyzed taken the same as the published lead SNP based on a sex-combined analysis, or did you pull out from this region the SNPs showing the greatest evidence for sexual dimorphic effects?

We have analysed the previously published lead SNPs from sex-combined analyses (Scott et al., Nat Genet 2012), so they are established variants as referred in the text. We also had the reference to the above-mentioned paper in the manuscript for the respective phrases.

4. Please provide a short description of Cochran's Q-test for heterogeneity.

We have added to the Statistical analysis section of the Methods the following text to give more details on the Cochran's statistic, as the Reviewer suggested:

"Cochran's statistic provides a test of heterogeneity of allelic effects at the jth SNP, and has an approximate chi-squared distribution with N_{j-1} degrees of freedom under the null hypothesis of consistency where N_j denotes the number of studies for which an allelic effect is reported."

5. In the analyses of established FI/FG loci: No loci showed significance evidence for heterogeneity in allelic effects on FG, and only 1 locus showing significant evidence for heterogeneity in allelic effects on FI. The FI-associated locus was significant at p=0.005, only slightly under the Bonferroni corrected threshold of p 0.002. This variant was significant in men only; but of interest, the variant

in IRS1, a locus where sex-specific effects have been previously reported for % body fat and lipids. Is this the same SNP? In some regards seems like the same result being reported, albeit with a highly correlated trait.

The lead variant reported by us is not the same SNP. Our SNP (rs2943645) is in LD (CEU r^2= 0.9778) with the previously reported SNP (rs2943650). In Kilpeläinen et al. study (Nat Genet 2011), rs2943650 near *IRS1* was associated with a 0.16% lower body fat percentage per copy of the major allele. The effect was stronger in men than in women (β=0.20% and β=0.06% per allele, respectively).
Consistent with the sex difference observed for the association of rs2943650 with body fat percentage, the associations with HDL cholesterol and triglyceride levels were more pronounced in men (n=9,937 and n=10,659, respectively) than in women (n=10,659 and n=10,848, respectively) ($P_{sexdifference}$=0.027 and P=0.025, respectively), whereas associations with indices of insulin resistance were similar in both sexes, most probably due to insufficient power. We appreciate Reviewer's observation about the effects observed for this locus in men for related phenotypes. This is not a unique feature of phenotypes that define so-called Metabolic syndrome, including lipids and obesity among others. For example, *FADS1* is an established FG and lipid locus and this locus has been reported for each trait association. Careful reporting of trait-specific associations is very important for dissection of pathophysiological changes in T2D and metabolic syndrome and for causality of relationships, which we indeed explore later in the manuscript.

6. Page 8, line 323: What is the rationale for obtaining only the sex-combined analyses from the 4 family-based studies? That is, couldn't sex-specific and sex-dimorphic analyses be obtained from these family designs also (e.g., as for the Amish)? As written, the implication is that it was because they were family studies, but perhaps the reason is these were the only results available?

Our investigation is a comprehensive discovery of loci with sex-combined and sex-dimorphic genetic effects on fasting glycaemic traits. It was therefore important to include as many studies in our analysis as possible to dissect sex-combined effects on FG and FI. The reason for obtaining only sex-combined analyses for the participating family-based studies was coming from the non-independence of parameter estimates in sex-specific/-dimorphic analyses because of relatives within families: by running male- and female-specific analyses, and then performing meta-analysis, we can't adequately account for the relatedness between males and females from the same family, violating the assumption of independence of results necessary for a valid meta-analysis.

7. Minor:
Page 7, line 280-281: replace "…whereas more women than men have elevated…" with "…whereas women [are more likely] than men to have elevated…"

We amended this in the manuscript following the Reviewer's suggestion.

Reviewer #3 (Remarks to the Author):
The authors have performed a genome-wide association study of up to 73,089 men and 67,506 women, to examine sex-dimorphism and sex-combined genetic effects on fasting glucose (FG) and fasting insulin (FI). They identify sex-heterogeneity in the effect of the previously identified IRS1 locus on FI, and identify a novel sex-dimorphic FI locus at ZNF12. Seven novel loci are reported for FG, none of which show sex-dimorphic effects. A causal effect of waist-hip ratio on FI in women but not in men is shown using a mendelian randomization analysis. Overall, the manuscript is well-written and includes several novel findings. I have the following remarks:

1. Lines 337-357: To study sex-dimorphic effects for the already established FG/FI loci, the authors use a two-step approach where they first, among all established FG/FI loci, identify the ones that show genome-wide significance in the 2 df joint model of the SNP main effect and interaction with sex, and in the second step, examine the 1 df SNP*sex interaction term with Bonferroni correction for the number of loci taken forward from the first step. I do not see this approach fully justified, in particular as the SNP*sex interaction term is part of the 2df model that is studied in the first step, and thus the second step in this approach is not completely independent from the first step. Thus, instead of looking at the 2df model at all, I suggest a simpler one-step approach where the authors examine the significance of the SNP*sex interaction term for all the previously known 36 FG and 19 FI loci, and run Bonferroni correction for the number of independent loci tested.

We acknowledge that the different approaches for defining statistical significance were confusing and we have, therefore, unified our approaches across the manuscript. Please see our responses to Reviewer #1, points 4 and 6.

2. Lines 412-424: In the genetic correlation analyses, it seems that the sex-specific results for FG and FI were correlated with sex-combined results for other GWAS traits, which makes the interpretation of the results tricky. It would be worthwhile to run additional analyses for the GWAS traits where sex-specific results are available (e.g. WHR, BMI), to study correlations between sex-specific results for FG and FI, and sex-specific results for the other GWAS traits.

We appreciate the suggestion by the Reviewer and have performed the proposed analyses (see Figure 3 below). The LDSC analyses highlighted the shared directionality of the sex-dimorphic effects between BMI and WHR obesity traits and glycaemic FG/FI traits. In all correlation analyses, the correlations were stronger in women than men. We have updated **Figure 3a/3b** by adding these results and updated the main text:

"*We estimated the genetic correlations between FG/FI and 201 traits with sex-combined GWAS summary statistics using the LD score regression (**Online methods, Figure 3a/b**). We detected genetic correlations between FI and 22 other traits (P<0.00012, corrected for multiple testing), including obesity-related phenotypes, leptin levels without adjustment for BMI, T2D, high-density lipoprotein cholesterol and triglycerides. Among those, we observed sex heterogeneity in the genetic correlations between FI and two traits: WHR adjusted for BMI (WHRadjBMI) (r_{Gwomen}=0.38, r_{Gmen}=0.20, $P_{Cochran'sQtest}$=0.015, I^2=83%) and WHRadjBMIfemales (r_{Gwomen}=0.40, r_{Gmen}=0.19, $P_{Cochran'sQtest}$=0.0099, I^2=85%) (**Figure 3a**). Furthermore, estimates for two of these traits were just marginally over the significance threshold for sex heterogeneity in their genetic correlation with FI: anorexia nervosa (r_{Gwomen}=-0.28, r_{Gmen}=-0.09, $P_{Cochran'sQtest}$=0.051, I^2=74%) and HOMA-B levels (r_{Gwomen}=0.67, r_{Gmen}=0.92, $P_{Cochran'sQtest}$=0.069, I^2=70%) (**Suppl. Table 5, Figure 3a**). Analysis of FG yielded statistically significant genetic correlations in both women and men with 13 traits including a number of obesity-related phenotypes, years of schooling, $HbA1_c$, and T2D (**Suppl. Table 6, Figure 3b**).*"

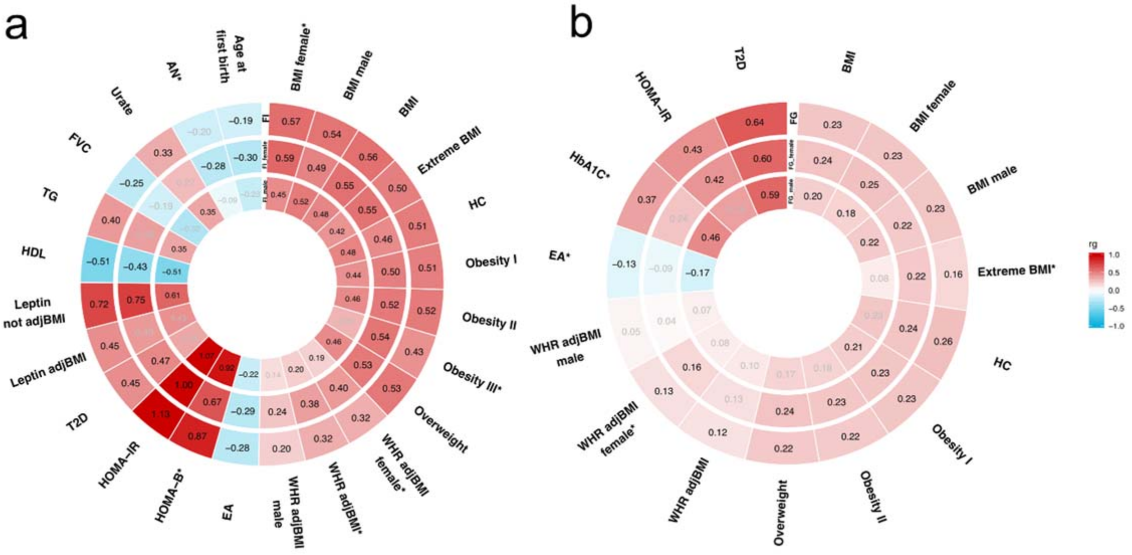

Figure 3a-b. Genetic correlations between fasting glycaemic and other traits by sex.

3. Lines 426-439: Was there evidence of horizontal pleiotropy in the WHR vs. FI mendelian randomization analysis, looking at the intercept from MR-Egger? If there was, it would be worthwhile to perform random effect analyses as well, and perhaps also MR-PRESSO analysis, to correct for horizontal pleiotropy.

The chosen method for the main MR analyses was the inverse variance weighted (IVW) meta-analysis of each instrumental variant using random effects. However this method is robust in scenarios when pleiotropic effects are balanced between the instrumental variants, as the Reviewer points out, the IVW method gives a biased estimate when pleiotropy is unbalanced, i.e. it exists on the level of the whole variant-set. To address this concern, we ran MR-Egger regression and now report both the intercept term and the slope of this sensitivity analysis for testing the presence of directional pleiotropy and providing an unbiased estimate. The positive causal relationship between WHR-FI in women holds in the MR-Egger analysis. We report these results in the main text as follows:

*"We observed a significant ($P_{Bonferroni}$<0.0125, corrected for four tests) causal effect ($\beta_{IV\text{-}WHRadjBMI\_exposure\_women}$=1.86, $P_{IV\text{-}WHRadjBMI\_exposure\_women}$=$1.9\times10^{-13}$) of WHRadjBMI on FI in women, but detected no causal effect in the reverse direction ($\beta_{IV\text{-}FI\_exposure\_women}$=0.55, $P_{IV\text{-}FI\_exposure\_women}$=0.03) nor in men in either direction ($\beta_{IV\text{-}WHRadjBMI\_exposure\_men}$=1.05, $P_{IV\text{-}WHRadjBMI\_exposure\_men}$=0.02; $\beta_{IV\text{-}FI\_exposure\_men}$=-0.01, $P_{IV\text{-}FI\_exposure\_men}$=0.26) (**Figure 3c, Suppl. Table 8**) under a random-effects inverse variance weighted model. To further investigate the robustness of the WHRadjBMI-FI causal relationship in women, we assessed the causal effect estimate from the MR-Egger method, which is less sensitive to pleiotropy. The intercept from the MR-Egger regression was estimated to be non-zero (Intercept=-0.002, $P_{Intercept}$=0.004) for the WHRadjBMI-FI relationship in women, to which a possible explanation is that pleiotropic effects of instrumental variables are not balanced or act randomly. If the non-zero MR-Egger intercept reflects unbalanced pleiotropy and therefore average pleiotropy over all instrumental variants, the slope of the MR-Egger regression provides an unbiased causal estimate. For the WHRadjBMI-FI causal relationship in women, we observed a significant MR-Egger causal estimate ($\beta_{IV\text{-}WHRadjBMI\_exposure\_women}$=3.11, $P_{IV\text{-}WHRadjBMI\_exposure\_women}$=$2.4\times10^{-9}$) robust to the presence of overall pleiotropy (Suppl. Table 8)."*

4. Lines 426-439: The weakness of using WHRadjBMI is that it does not allow to tell whether the causal effect on FI is driven by waist circumference (abdominal fat) or hip circumference (gluteofemoral fat), which is highly relevant when talking about sexually dimorphic effects. Thus, it would be greatly helpful to see additional analyses for waist circumference adjusted for BMI and hip circumference adjusted for BMI, to tell which fat depot drives the causal relationship with FI in men and women.

The Reviewer raised a valuable point, which we address below and include the results from the new analysis to the main text, as well as in supplementary material. Our analysis primarily indicated that there is a causal effect between WHR adjusted for BMI and FI in women only. Further analysis, suggested by the Reviewer, has highlighted that the causal effect of WHR on FI, when split into waist circumference adjusted for BMI and hip circumference adjusted from BMI, is driven by the effect of waist circumference adjusted for BMI on FI. In particular, we have performed analyses of four causal relationships: waist on FI in women and men, hip on FI in women and men (see Figure 1 below). There is also a moderate inverse relationship between hip circumference and FI in women, although that wouldn`t survive correction for multiple testing. Neither waist nor hip circumference show a causal effect on FI in men. We haven`t tested the reverse relationship (FI on either waist or hip circumference), since we didn`t observe an effect in that direction for WHR either. In women, waist circumference has a direct causal effect on FI levels. We therefore conclude that higher abdominal fat has causal effect on insulin resistance in women. We have also updated the main text as below:

"We further observed that abdominal fat (defined through waist circumference with adjustment for BMI [WCadjBMI], 222 independent SNPs in 214,924 UK Biobank individuals) is the driving factor ($\beta_{IV\text{-}WCadjBMI\_exposure\_women}$=0.015, $P_{IV\text{-}WCadjBMI\_exposure\_women}$=$5.3\times10^{-8}$) of the WHR causal effect on FI in women. Gluteofemoral fat (defined as hip circumference with adjustment for BMI [HCadjBMI], 274 independent SNPs in 183,739 UK Biobank individuals) exerted a moderate inverse causal effect on FI in women ($\beta_{IV\text{-}HCadjBMI\_exposure\_women}$=-0.01, $P_{IV\text{-}HCadjBMI\_exposure\_women}$=0.004. There was no detectable causal effect of WCadjBMI or HCadjBMI on FI in men ($\beta_{IV\text{-}WCadjBMI\_exposure\_men}$=0.001, $P_{IV\text{-}WCadjBMI\_exposure\_men}$=0.81; $\beta_{IV\text{-}HCadjBMI\_exposure\_men}$=-0.001, $P_{IV\text{-}HCadjBMI\_exposure\_men}$=072)."

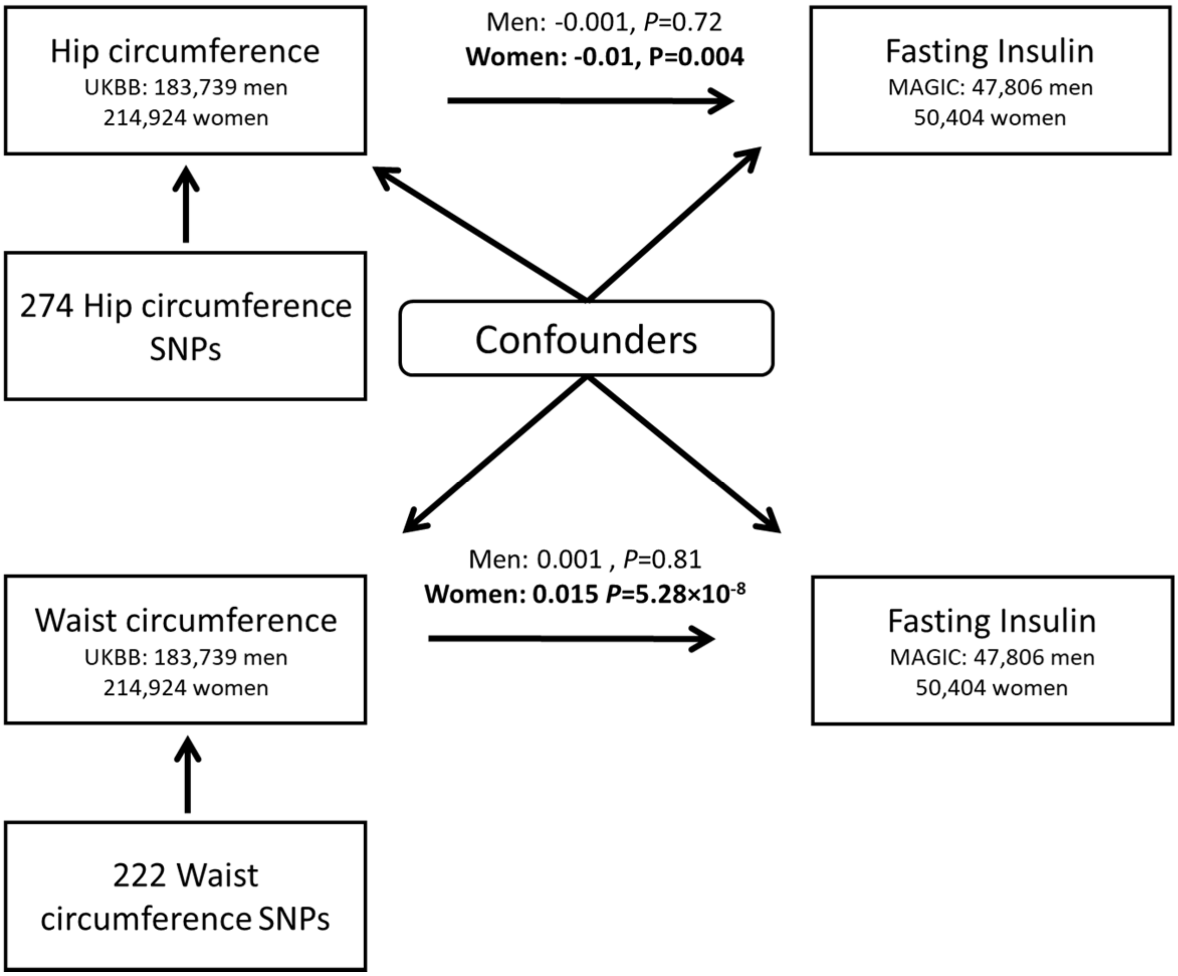

Figure. *Mendelian Randomization analysis between waist and hip circumference and fasting insulin.*

5. Lines 487-488, 577-579, and 588-589: It is true that beta cells are highly relevant to glucose metabolism, but I find it surprising that a locus associated with insulin resistance in non-diabetic individuals is linked to beta-cells. How would the authors interpret this intriguing finding?

We are thankful to the Reviewer for this interesting and relevant observation. It is a common knowledge that insufficient insulin secretion in the face of insulin resistance leads to T2D. As we highlight in the manuscript, studies of glycaemic traits, and those in fasting state specifically in individuals within the range outside of diabetes-defining values of these phenotypes, have been successful in highlighting the genetic variability important for T2D pathogenesis. The so-called pre-diabetes status is developed when fasting glucose levels are 6mmol/l or higher, and 80% of individuals that reach this state will continue into developing the disease with time. The research of this manuscript's authors has clearly shown that over 1/3 of loci associated with fasting glycaemic traits are also associated with susceptibility to T2D (Marullo et al., Curr Diab Reps 2014; Scott et al., Nat Genet 2012; Manning et al., Nat Genet 2012). The above observation also highlights that within normal range there will be individuals on the path of developing the disease. Thus, studying individuals within normal range will present us with loci that are important for T2D susceptibility as well as those that contribute to the maintenance of glucose homeostasis.

To address the importance of relationship between beta-cell function and insulin resistance, we can present two lines of evidence. First, we provide support for a similar FI locus with

effects on beta-cell function detected in experimental studies. A well-established locus at *IGF1* gene is associated with FI and has no nominal effect on (not associated with) FG (Scott et al., Nat Genet 2012). However, Insulin-like growth factor 1 (IGF1) has been implicated in islet development and differentiated function (Bonner-Weir et al., Trends Endocr and Metabolism 1994). Other studies also highlighted that β-cell-specific deletion of the *Igf1* receptor leads to hyperinsulinemia and glucose intolerance through control of differentiated β-cell function (Kulkarni et al., Nat Genet 2002). This example clearly shows the importance of evaluation of the effects of genes located in the loci associated with fasting insulin (as well as those associated with other glycaemic traits) in the pancreatic islets and more specifically the investigation of their expression in this tissue. Additionally, the reference data freely available in public domain does not provide appropriate evaluation of expression patterns, since pancreatic tissue contains several types of cells with diverse expression patterns, that require appropriate extraction and evaluation. In fact, we have done dissection of expression in both beta-cells and islets in our study. Second, the experimental design we took forward within this study, was to obtain new information about expression patterns and function of specific genes in this locus and within other loci, for which almost any information was lacking in the public domain. We, thus, aimed to obtain more insight about their biology. The dissection of such genes expression was done in a range of tissues, as we describe in the manuscript. We conducted the pancreatic islet and flow-sorted beta-cell expression along with the panel of other tissues represented a targeted experiment to add new biological knowledge. For *ZNF12*, we note that its expression is detected in multiple tissues, while in our experiment we detected in pancreatic beta-cells and islets it is stronger than in other tissues studied. The mechanism for such expression pattern might be related to its cellular location in the nucleoplasm (https://www.proteinatlas.org/ENSG00000164631-ZNF12#gene_information) and contribution to intracellular processes. The latter potentially could be important for beta-cells and islets function with subsequent effects on glucose homeostasis through insulin resistance. We amended the main text phrase as shown below:

"The novel female-specific FI locus is at ubiquitously expressed ZNF12, encoding for zinc-finger protein 12, localised in nucleoplasm of cells and involved in developmental control of gene expression."

We have previously omitted by mistake a figure with similar expression pattern for the novel FG locus detected in sex-combined analysis at *RGS17* and the expression pattern (as reported below). We have now added this figure to the supplementary materials. We also note the *RGS17* shows expression in beta cells and pancreatic islets.

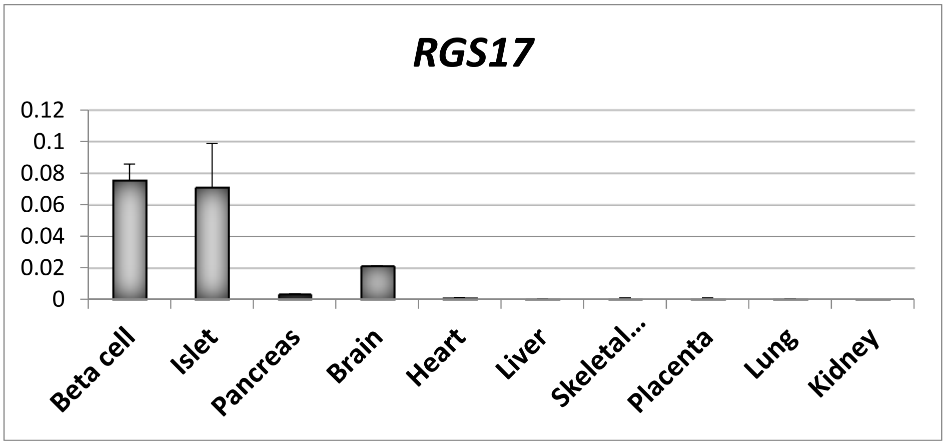

Supplementary Figure 1. *Legend:* ***e) RGS17 tissue expression*** *relative to three housekeeping genes (PPIA, B2M and HPRT). Beta cell (n=3) and islets (n=3) data are expression means ± standard deviation. Quantitative RT-PCR was carried out using cDNAs from three human donors (beta-cells and*

islets). The error bar is only presented for the two first tissues (beta cells and islets, three individuals tested). The other tissues were commercial cDNAs (one point observation).

6. Figure 3: As leptin is produced directly in proportion to total body fat mass, leptin levels and BMI are generally very strongly correlated. The genetic correlation analysis was performed for leptin not adjusted BMI. A more interesting question is, was there a correlation between FI and leptin levels adjusted for BMI?

The Reviewer has highlighted a very valuable point. Since leptin is, in fact, an adipocyte-secreted hormone that influences long-term regulation of energy homeostasis by informing the brain about the amount of stored body fat, it is natural to explore, how leptin levels, independent of BMI are genetically correlated with fasting insulin, where the r_g values from LDSC analysis survived the correction for multiple testing and were therefore included in the manuscript (Figure 3a). The same study (Kilpeläinen et al., Nat Commun 2016) results contained an additionally GWAS for leptin adjusted for BMI. We performed the LDSC analysis for this phenotype and fasting insulin and show below the figure demonstrating the results of genome-wide genetic correlation for both leptin traits (**Figure 2**). This additional analysis suggests less strong genetic correlation between leptin adjusted for BMI and fasting insulin and the difference between sexes is indistinguishable. However, the sex-combined correlation still survived multiple testing correction ($P<1.25\times10^{-4}$) highlighting leptin among the traits genetically correlated with fasting insulin levels. We have updated the main text Figure 3a to add this track and amended the main text to mention leptin adjusted for BMI data – see below the phrase.

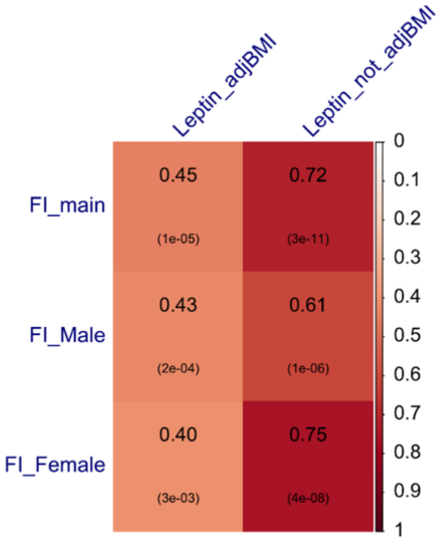

Figure 2. *Genetic correlations genome-wide between fasting insulin and leptin levels with/without adjustment for BMI. The top track shows the estimate for all individuals together, followed by the estimates for males and females, respectively.*

Main text:

"We detected genetic correlations between FI and 22 other traits (P<0.00012, corrected for multiple testing), including obesity-related phenotypes, leptin levels without adjustment for BMI, T2D, high-density lipoprotein cholesterol and triglycerides."

Figure 3. Genetic correlations and causality: a) genetic correlation for FI, b) genetic correlations for FG, c) bi-directional MR analysis between WHRadjBMI and FI. *Phenotypes with statistically significant (P<0.00012) genetic correlations with FI/FG in either women or men are plotted. The outer track shows estimates for all together, followed by those for women and men. Traits with I^2 (sex heterogeneity) ≥50% are labelled with asterisks. Grey colour indicates traits that do not show significant genetic correlation with the given glycemic trait. Estimates in black colour indicate statistically significant associations. AN, anorexia nervosa; BMI, body-mass index; EA, educational attainment as of years of schooling 2016; FVC, forced vital capacity; HbA1c, glycated haemoglobin; HC, hip circumference; HDL, high-density lipoprotein cholesterol; HOMA-B, homeostatic model assessment of beta cell function; HOMA-IR, homeostatic model assessment of insulin resistance; Leptin adjBMI, leptin adjusted for BMI; Leptin not adjBMI, leptin not adjusted for BMI; Obesity 1, obesity class 1; Obesity II, obesity class II; Obesity III, obesity class 3; T2D, type 2 diabetes; TG, triglycerides; WC, waist circumference; WHR adjBMI, waist-to-hip ratio adjusted for BMI; UKBB, UK Biobank.*

REVIEWERS' COMMENTS:

Reviewer #1 (Remarks to the Author):

The authors adequately addressed my previous concerns and I have no further comments.

Reviewer #2 (Remarks to the Author):

no add'l comments

Reviewer #3 (Remarks to the Author):

The authors have addressed all my comments and questions appropriately. Thus I am pleased to recommend the paper to be accepted for publication.
